# Hexahalodiborate Dianions: A New Family of Binary Boron Halides

**DOI:** 10.1002/anie.201906666

**Published:** 2019-08-23

**Authors:** Guillaume Bélanger‐Chabot, Holger Braunschweig

**Affiliations:** ^1^ Institut für Anorganische Chemie Julius-Maximilians-Universität Würzburg Am Hubland 97074 Würzburg Germany; ^2^ Institute for Sustainable Chemistry & Catalysis with Boron Julius-Maximilians-Universität Würzburg Am Hubland 97074 Würzburg Germany

**Keywords:** binary species, boron, electron-precise diborates, halogens

## Abstract

The electron‐precise binary boron subhalide species [B_2_X_6_]^2−^ X=F, Br, I) were synthesized and their structures confirmed by X‐ray crystallography. The existence of the previously claimed [B_2_Cl_6_]^2−^, which had been questioned, was also confirmed by X‐ray crystallography. The dianions are isoelectronic to hexahaloethanes, are subhalide analogues of the well‐known tetrahaloborate anions (BX_4_^−^), and are rare examples of molecular electron‐precise binary boron species beyond B_2_X_4_, BX_3_, and [BX_4_]^−^.

## Graphical abstract

**New classics**! The first electron‐precise haloborate anions beyond [BX_4_]^−^ are structurally characterized. [B_2_X_6_]^2−^ (X=F, Cl, Br, I) species were fully characterized as their organoammonium or ‐phosphonium salts and insight into their formation and thermodynamic stability is provided by quantum chemical calculations.

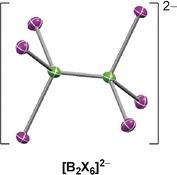

Binary species are fundamental to the systematic understanding of an element and its chemistry. Binary halogen species,^1^ in particular, are among the most important, not only because, owing to their high electronegativity, they can form compounds with most elements and are as such well‐represented across the periodic table, but also because they are typically either very reactive (ClF_3_, SbCl_5_, NCl_3_, etc.) or very stable ([PF_6_]^−^, [BF_4_]^−^, etc.) Boron halides are, along with hydrides, arguably the most important class of binary boron species, and include a wide variety of cluster‐type species such as B_4_Cl_4_, B_9_X_9_, [B_9_X_9_]^2−^, etc.^1^ Electron‐precise boron halides (i.e., that possess only classical two‐electron bonds), however, are limited to haloboranes (BX_3_), tetrahaloborates (BX_4_^−^), the four diboron subhalides B_2_X_4_,^2^ the transient species BX, and the claimed^3^ subhalide [B_2_Cl_6_]^2−^, for which structural evidence is lacking (Figure 1). [B_2_F_6_]^2−^ is also mentioned in the patent literature with little data available to support its existence.^4^

**Figure 1:**
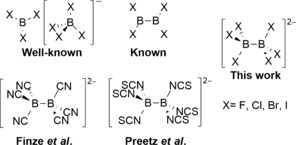
[B_2_X_6_]^2−^ species in the context of the other known electron‐precise binary halogen compounds (top left) and electron‐precise homoleptic diborate(6) pseudohalides (bottom left).

Because of the instructional importance of the tetracoordinate borate anions [BX_4_]^−^, which demonstrate the Lewis acidic character of the corresponding boranes BX_3_, we wondered whether the analogous subhalide species [B_2_X_6_]^2−^ could3a, 3c exist. Indeed, their Lewis acidic B_2_X_4_ counterparts are all highly reactive and most of them are rather unstable and decompose to BX_3_ and to larger boron subhalides at various rates at room temperature. The fact that the two charges in [B_2_X_6_]^2−^ are delocalized over only eight atoms (in contrast to the known, closely related pseudohalide derivatives [B_2_(CN)_6_]^2−^ and [B_2_(NCS)_6_]^2−^, which both bear polyatomic ligands)^5^ could potentially make them prohibitively unstable. We herein report on the successful isolation and full characterization of the four hexahalodiborate anions as organophosphonium and/or ‐ammonium salts.

Given the convenient synthetic route to all four B_2_X_4_ precursors from B_2_Br_4_, published recently by our group,^6^ the direct nucleophilic addition of the X^−^ group to the appropriate B_2_X_4_ was open for exploration. When B_2_Cl_4_ and B_2_Br_4_ were treated with tetraphenylphosphonium chloride and bromide, respectively, in dichloromethane solutions, white crystalline material precipitated after a few minutes at room temperature. X‐ray diffraction experiments showed the material to be [PPh_4_]_2_[B_2_Cl_6_]⋅2 CH_2_Cl_2_ ([PPh_4_]_2_[**2**]⋅2 CH_2_Cl_2_) and [PPh_4_]_2_[B_2_Br_6_]⋅2 CH_2_Cl_2_ ([PPh_4_]_2_[**3**]⋅2 CH_2_Cl_2_), respectively (Scheme 1). Both compounds exhibited little to no solubility in dichloromethane, as judged by ^11^B NMR spectroscopy.

**Scheme 1:**
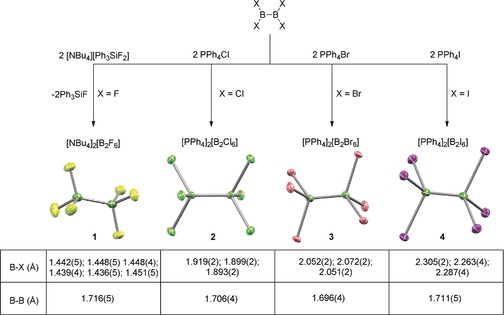
Synthesis of salts of the hexahalodiborates [B_2_X_6_]^2−^ **1**–**4** in dichloromethane (top) and solid‐state structures from single‐crystal X‐ray diffraction with selected structural parameters (bottom). **1** was structurally characterized as [PPh_4_]_2_[B_2_F_6_]⋅[PPh_4_]Br⋅CH_3_CN, **2** and **3** as [PPh_4_]_2_[B_2_X_6_]⋅2 CH_2_Cl_2_, and **4** as [PPh_4_]_2_[B_2_I_6_].

The isolation of the remaining [B_2_F_6_]^2−^ (**1**) and [B_2_I_6_]^2−^ (**4**) salts was less straightforward. Because of the scarcity of anhydrous, soluble fluoride salts, their low stability and/or high reactivity, we chose tetrabutylammonium triphenyldifluorosilicate (TBAT) as a mild, anhydrous source of fluoride anions. Upon treatment of B_2_F_4_ with two equivalents of TBAT in dichloromethane from −196 °C to ambient temperature, a new species with a ^11^B NMR signal at 5.8 ppm and a ^19^F NMR signal at −129.3 ppm was observed, as well as the expected Ph_3_SiF byproduct. Upon washing with diethyl ether and pentane to remove Ph_3_SiF, a somewhat waxy solid was obtained. Attempts at crystallization failed, however, and only prism‐shaped, soft, glassy material was obtained. The assignment of the compound as a [B_2_F_6_]^2−^ salt could be confirmed by the addition of [PPh_4_]Br to acetonitrile solutions of [TBA]_2_[**1**], which yielded crystals of [PPh_4_]_2_[**1**]⋅[PPh_4_]Br⋅CH_3_CN of sufficient quality for X‐ray diffraction, which confirmed the existence of the hexacoordinate dianion **1** (Scheme 1).

The treatment of B_2_I_4_ with TBAI in dichloromethane yielded solutions that displayed ^11^B NMR signals that shifted to higher fields as the iodide content increased, indicative of complex equilibria in solution. Such behavior is also observed for the I^−^/BI_3_/[BI_4_]^−^ system.^7^ At 10 equivalents of iodide, one major signal was observed at *δ*(^11^B)=−29 ppm, which was assigned to **4**. Relying on the expectedly lower solubility of salts of the dianionic **4** compared to that of the putative [B_2_I_5_]^−^, a plausible intermediate, we hoped that **4** would be the first species to crystallize even from equilibrium mixtures. Indeed, a toluene/dichloromethane solution of 2[PPh_4_]I/B_2_I_4_ at −30 °C yielded X‐ray diffraction quality crystals of [PPh_4_]_2_[**4**] (Scheme 1). Depending on the conditions, crystals of [PPh_4_]_2_[**4**]⋅2 CH_2_Cl_2_ could also be obtained (see the Supporting Information). So far, our attempts at identifying the putative [B_2_X_5_]^−^ intermediate species have yielded only the [BX_4_]^−^ decomposition product instead (see the Supporting Information for crystal structures of [PPh_4_][BBr_4_] and [PPh_4_][BI_4_]).

The crystal structures of salts of **1**–**4** (Scheme 1) demonstrate that the B−B bond remains intact in these species. The [B_2_X_6_]^2−^ dianions were found in a staggered conformation of approximate *D*_3*d*_ symmetry. In the solid state, the [B_2_X_6_]^2−^ species in salts of **2**–**4** possess a crystallographic inversion center. B−B bond lengths were mostly unaffected by the halogen substituents and were found to be identical within standard deviations, in the 1.7 Å range (Scheme 1), in agreement with computationally predicted values (Table 1). This contrasts with predicted B−B bond lengths for the parent B_2_X_4_,^8^ which steadily decrease from 1.725 Å (X=F) to 1.654 Å (X=I) (Table 1). The observed B−B bond lengths are also in stark contrast with predicted values for the more weakly bound hypothetical [B_2_X_6_]^.−^ radical anions (>2.11 Å).^9^ The B−X bond lengths in **1**–**4** (Scheme 1) are all significantly larger than those predicted for B_2_X_4_ (Table 1). The B–X bond lengths in **1**–**4** are comparable to, although noticeably longer than, the B−X bond length in the respective [BX_4_]^−^ anion. The B−X bond lengths within the BX_3_ moieties of the asymmetric units of **1**–**4** vary to some degree (Scheme 1), reflecting varying degrees of interactions with the cations or cocrystallized solvent with each halogen substituent in the solid state. Similar variations are observed in [BX_4_]^−^ (Table 1).^10^, ^11^, ^12^

**Table 1:** Comparison of selected structural parameters for **1**–**4** and related boron halide species (all values in Å).

	B_2_X_4_^[a]^		[B_2_X_6_]^2−[b]^		[BX_4_]^−^
X	B−B	B−X	B−B	B−X	B−X
F	1.725	1.330	1.743	1.461	1.383^13^
Cl	1.688	1.756	1.737	1.916	1.844(2), 1.852(2), 1.866(2), 1.864(2)^10^
Br	1.678	1.922	1.743	2.083	2.0179(4)^[c]^
I	1.654	2.137	1.734	2.316	2.2336(3)^[d]^

[a] Predicted values for *D*_2*d*_ geometry.^8^ See also ref. 14. [b] Predicted values (this work) at the ωb97xd/6‐311+g(d,p)‐SMD/PCM level. [c] This work (see the Supporting Information), see also ref. 11. [d] This work (see the Supporting Information), see also ref. 12.

Solid‐state ^11^B NMR spectra for of salts of **2**–**4** were acquired. In addition, solution ^11^B NMR spectra for [TBA]_2_[**1**], in‐situ prepared [PPN][**2**] (PPN=bis(triphenylphosphoranylidene)ammonium) and [TBA]_2_[**3**], and for the [TBA]I/B_2_I_4_ system were acquired. The *δ*(^11^B) values are compiled in Table 2 and are in good agreement with predicted ones (see the Supporting Information). Moreover, values obtained from solution phase are in good agreement with those from the solid state. As expected, the ^11^B NMR shifts for **1**–**4** are all at significantly higher field than those for the parent B_2_X_4_. The ^19^F NMR signal of **1** is also significantly upfield (*δ*(^19^F)=−129.3) compared to B_2_F_4_ (*δ*(^19^F)=−55 ppm).^6^ The ^11^B NMR shifts of **2**–**4** are, however, comparable to that of the hexacoordinate B_2_X_4_ **⋅**2 SMe_2_. The strong shielding effect of Br^−^ and I^−^ found for [BBr_4_]^−^ (*δ*(^11^B)=−24)^6^ and [BI_4_]^−^ (*δ*(^11^B)=−127)^14^ have no equivalent in **3** and **4**.

**Table 2:** Comparison of ^11^B NMR chemical shifts (in ppm) for **1**–**4** and related boron halide species.

X	B_2_X_4_ 2c, 6	[B_2_X_6_]^2−^ (predicted)^[a]^ [solid‐state]	B_2_X_4_⋅2 SMe_2_ 6
F	24	5.9 (4.7)	–
Cl	62	11.7 (8.0) [12.3]	7
Br	70	1.5 [−4.5]	0
I	70	−29.9 [−34.8]	−20

[a] Predicted values at the ωb97xd/6‐311+g(d,p)‐SMD/PCM (GIAO) level (see the Supporting Information).

The vibrational spectra (Raman and IR) of the [B_2_X_6_]^2−^ salts are dominated by the intense and numerous bands belonging to the organic cations. Nevertheless, the observed spectra are consistent with the predicted vibrational spectra of [B_2_X_6_]^2−^ (see the Supporting Information). Moreover, the comparison of spectra with those reported^15^ for known B_n_X_n_ derivatives and [BX_4_]^−^ supports the assignment of the isolated materials as [B_2_X_6_]^2−^ salts and not as decomposition products. Some intense characteristic vibrations for the [B_2_X_6_]^2−^ anions could be unambiguously observed and tentatively assigned with the aid of quantum chemical calculations (see the Supporting Information for a detailed discussion). The bands for the B−X stretching modes are the least ambiguous and are listed in Table 3.

**Table 3:** Comparison of selected vibrational B−X stretching frequencies for **1**–**4** and related boron halide species (all values in cm^−1^). r=Observed only in Raman spectra. All bands for [B_2_X_6_]^2−^ are otherwise exclusively observed in IR spectra.

	B_2_X_4_		[B_2_X_6_]^2−^		[BX_4_]^−^	
X	ν(BX_2_) sym	ν(BX_2_) asym	ν(BX_3_) sym	ν(BX_3_) asym	ν(BX_4_) sym	ν(BX_4_) asym
F	1151^16^; 673r^17^	1375^16^; 1368r^17^	886; 626r	843;(850r)^[a]^	ca. 780r^18^	ca. 1100^18^
Cl	728; 401r^19^	917^19^	554/569; 354r	591/601; 641r	405r15b	67015b
Br	592^20^; 245r	777^20^	498^[b]^; 214r	498^[b]^; (560/576)r	243r15b	60515b
I	4932c; –	710/6802c	464^[b]^; 200r	464^[b]^; (486/517/534)r	–	51715a

[a] Not observed, predicted value (ωb97xd/6‐311+g(d,p)‐SMD/PCM) supplied instead. [b] The broad band observed in the IR spectra likely corresponds to both ν(BX_3_) sym and ν(BX_3_) asym.

The IR band observed for **2** (complex band centered at 588 cm^−1^) is in qualitative agreement with the reported3a IR bands at 694, 665, and 600 cm^−1^. The bands at 665 and 694 cm^−1^ previously reported for **2** are problematic, as they coincide with known bands of [BCl_4_]^−^,3a, 15b a very common side‐product of **2**. However, they are also very close to the Raman‐allowed in‐phase B−Cl asymmetric stretching mode, which might have been IR‐allowed in the solid state due to lower site symmetry. The B−B stretch could not be observed, presumably obscured by the cation bands in the 1000–1200 cm^−1^ region. Expectedly, B−X stretching frequencies decrease as the size of the halogen increases, as is the case for [BX_4_]^−^, and they are at lower frequency than for the parent B_2_X_4_ (Table 3).

We conducted quantum thermochemical calculations to verify the stability of [B_2_X_5_]^−^ and [B_2_X_6_]^2−^ with respect to the loss of a halide ligand. The Gibbs free energy change was estimated in dichloromethane solutions for the single halide addition to B_2_X_4_ and to [B_2_X_5_]^−^, respectively (Scheme 2). Difficulties in correctly accounting for specific solvation effects for our charged species are expected to lead to significant uncertainties. Nevertheless, our computational estimates allow us to identify important qualitative trends. Interestingly, our computed Gibbs free energy change for the first halide addition (Scheme 2, top), yielding [B_2_X_5_]^−^, is comparable to that of the halide addition to the monoboranes to yield [BX_4_]^−^ (Scheme 2, bottom).^21^ Unsurprisingly, the second halide addition to yield [B_2_X_6_]^2−^ is always significantly less favorable than the first, to the point that the formation of [B_2_X_6_]^2−^ from [X]^−^ and [B_2_X_5_]^−^ is predicted to be (somewhat) endergonic for X=Cl, Br and, by extrapolation, I (Scheme 2, center). Qualitatively, this suggests that the isolation of salts of **2**, and in particular **3** and **4**, should be strongly affected by subtle differences between the solvation and the lattice enthalpies of the salts, in agreement with our experimental observations in the case of **4**, which could be isolated because because its salts are less soluble than salts of [B_2_I_5_]^−^. **2** and **3** are more borderline cases and appear to be stable species in solution (see the Supporting Information), in agreement with conductimetric measurements on **2**.3a

**Scheme 2:**
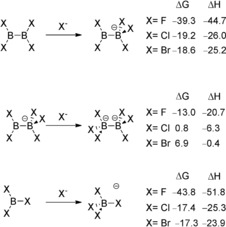
ωb97xd/6‐311+g(d,p)‐SMD/PCM estimates for Gibbs free energy and enthalpy changes for the formation of pentahalodiborate monoanions [B_2_X_5_]^−^ (top), hexahalodiborate dianions [B_2_X_6_]^2−^ (center), and tetrahaloborates [BX_4_]^−^ (bottom) in dichloromethane solution (all values in kcal mol^−1^).

The dianions **2**–**4** proved to be kinetically significantly more stable than their respective tetrahalodiborane counterparts at room temperature. BX_3_ is the most common decomposition product of B_2_X_4_ and the only one that is easily detectable by ^11^B NMR spectroscopy. Consequently, we found [BX_4_]^−^ to be the main detectable decomposition products of [B_2_X_6_]^2−^ and even encountered them in single‐crystal X‐ray characterizations (see the Supporting Information). Upon storage at room temperature, solid samples of [PPh_4_]_2_[**2**] and [PPh_4_]_2_[**3**] still contained X‐ray diffraction quality crystals of the relevant species after months, whereas the parent B_2_X_4_ compounds have half‐lives of days to weeks at the most. In solution, the decomposition of **1** and **4** occurred to a detectable extent within a few days at room temperature. Salts of **4**, in particular, are not stable in dichloromethane solutions and decompose to a significant extent to [BI_4_]^−^ and other unidentified species within days at room temperature. **1**–**4** appear to be indefinitely stable in the solid state when kept at −30 °C. In contrast to [BF_4_]^−^, even the rather unreactive **1** is moisture‐sensitive.

The [B_2_X_6_]^2−^ dianions are members of a rather limited family of negatively charged diborane species,^5^, ^22^ and of an even more limited family of homoleptic dianionic diborane species.3a, 5, 22n–22p, 23 Although conductimetric, IR spectroscopic, and elemental analysis data have been reported for salts of **2**,3a, 3b no further data nor a structural confirmation could be found in the literature. Moreover, another study has cast doubt on the results of the initial report (see the Supporting Information for further discussion).3c The most closely related examples are the homoleptic dianionic species [B_2_(CN)_6_]^2−[5a]^ and [B_2_(NCS)_6_]^2−^.5b These fascinating species have not yet been made by simple reactions between X^−^ and B_2_X_4_ (where X=CN or NCS).

In conclusion, three rare examples of new electron‐precise boron–halogen binary species were isolated and fully characterized, and the existence of the previously claimed [B_2_Cl_6_]^2−^ was confirmed by X‐ray crystallography and NMR and Raman spectroscopy. Species **1**–**4** are isoelectronic to the carbon binary halides C_2_X_6_ and, in analogy to these, could potentially display interesting photodissociative and halogenation behaviors.^24^ In the context of the renewed interest in electron‐precise diborane chemistry^25^ and of the likely involvement of diborane‐based anions in metal‐free borylation reactions,22c, 22d, 22i, 26 species **1**–**4** add fundamental knowledge^27^ to catalytically relevant systems in a rapidly advancing field.^28^

CCDC https://www.ccdc.cam.ac.uk/services/structures?id=doi:10.1002/anie.201906666 contain the supplementary crystallographic data for this paper. These data can be obtained free of charge from http://www.ccdc.cam.ac.uk/.

## Conflict of interest

The authors declare no conflict of interest.

## Supporting information

As a service to our authors and readers, this journal provides supporting information supplied by the authors. Such materials are peer reviewed and may be re‐organized for online delivery, but are not copy‐edited or typeset. Technical support issues arising from supporting information (other than missing files) should be addressed to the authors.

Supplementary
