# Supplementary material for: Hexahalodiborate Dianions: A New Family of Binary Boron Halides
Source: Angew Chem Int Ed Engl. 2019 Aug 23;58(40):14270–4. doi: 10.1002/anie.201906666 (PMC7028027; doi:10.1002/anie.201906666)
Supplement: Supplementary file 1 — Supplementary [file ANIE-58-14270-s001.pdf]

Angewandte Chemie
Eine Zeitschrift der Gesellschaft Deutscher Chemiker

Supporting Information

Hexahalodiborate Dianions: A New Family of Binary Boron Halides

*Guillaume Bélanger-Chabot and Holger Braunschweig**

anie_201906666_sm_miscellaneous_information.pdf

Methods and Materials 2
Synthesis, Characterization, Spectroscopic Details and Further Discussion 3
X-ray Crystallographic Details 52
Cartesian Coordinates of Optimized Structures 70
References 79

Methods and Materials

All manipulations were performed either under an atmosphere of dry argon or *in vacuo* using standard Schlenk, high vacuum line or glovebox techniques. Deuterated solvents were dried over molecular sieves and degassed by three freeze-pump-thaw cycles prior to use. All other solvents were distilled and degassed from appropriate drying agents. Solvents (both deuterated and non-deuterated) were stored under argon over activated 3 or 4 Å molecular sieves. Liquid-phase NMR spectra were acquired on a Bruker Avance 400 MHz NMR spectrometer (^{1}H: 400.1 MHz, ^{11}B: 128.4 MHz, ^{19}F: 470.6 MHz) or on a Bruker AVANCE-I 200 MHz NMR. Chemical shifts (δ) are given in ppm and internally referenced to the solvent signal (^{13}C NMR) or the residual protic signal (^{1}H NMR) of the solvent. ^{11}B and ^{19}F chemical shifts were externally referenced to [BF_3·OEt_2] and $CFCl_3$, respectively. The solid-state magic-angle spinning (MAS) NMR spectra were recorded on a Bruker Avance Neo 400 WB spectrometer operating at 400.13 MHz for ^{1}H, 128.38 MHz for ^{11}B, 100.61 MHz for ^{13}C, and 161.98 MHz for ^{31}P, using a 4 mm (o.d.) ZrO_2 rotor. Chemical shifts were calibrated for all nuclei externally by adjusting the field manually, so that the low-field shift of adamantane appears at 38.48 ppm. The ^{11}B solid-state MAS spectra were acquired by a rotor-synchronized Hahn-Echo (RSHE) at a spinning speed of 14.5 kHz and the second order quadrupolar powder pattern of **2** (Figure S9) was simulated with the software package SOLA[1] within Topspin™ 4.06 by Bruker Biospin. The ^{11}B NMR lineshapes in the solid-state spectra for **3** and **4** displayed only broad unstructured Gaussian signals which could be characterized by the maximum and the FWHH. The ^{13}C and ^{31}P solid-state NMR spectra were obtained using a CP/MAS sequence at spinning speeds between 8-11 kHz and 14-14.5 kHz, respectively.

Raman spectra were acquired on a Bruker IFS-120 with a Nd/YAG excitation laser (1064 nm) at a power of 100-300 mW in melting point capillaries. IR spectra were acquired in the argon atmosphere of a glovebox on a Bruker Alpha spectrometer equipped with an ATR module. All vibrational spectra were acquired at ambient temperature (20-30 °C).

Tetraphenylphosphonium bromide and iodide were purchased from TCI, other reagents were purchased from Sigma Aldrich. B_2F_4,[2] B_2Cl_4,[3] B_2Br_4[4] and B_2I_4[5] were prepared using literature procedures.[6] B_2F_4, B_2Cl_4 and B_2Br_4 were handled and purified using high-vacuum line techniques and amounts were measured using a combination of weighing and/or pressure measurements in a vacuum line with calibrated volumes.

Synthesis, Characterization, Spectroscopic Details and Further Discussion

$[TBA]_2[B_2F_6]$

Tetrabutylammonium triphenyldifluorosilicate (228 mg, 0.423 mmol) was placed into a reaction vessel. Dichloromethane (5-10 mL) was condensed over the solid at –196 °C. The mixture was homogenized at room temperature, then frozen at –196 °C. B_2F_4 (0.21 mmol) was condensed over the frozen solution at –196 °C. The mixture was thawed at –96 °C and slowly warmed to room temperature under stirring. After 2 h of stirring, the volatiles were removed under vacuum. The resulting sticky paste was rinsed several times with diethyl ether and pentane to remove the Ph_3SiF byproduct and the resulting solid was dried *in vacuo*. The consistency of the isolated material was very sensitive to small amounts of residual Ph_3SiF and other impurities. When the compound is still gel-like, it can be dissolved in dichloromethane and precipitated by adding hexane and cooling at –30 °C. This resulted in a white gel that was washed with toluene, ether and pentane and dried in the argon atmosphere of a glovebox, yielding $[TBA]_2[B_2F_6]$ as a colorless, somewhat tacky solid of sufficient purity (69 mg, 56 mol% recovered yield based on B_2F_4). Analytically pure samples were obtained by repeated recrystallization from DCM/ether at –30 °C. Crystals suitable for X-ray diffraction were obtained by adding 1-2 equivalents of $[PPh_4]Br$ to acetonitrile or DCM solutions of $[TBA]_2[B_2F_6]$ and storing the solution at –30 °C for 1-2 days.

NMR (200 MHz, CD_3CN, 296 K) δ/ppm: ^{1}H 3.12 (m, 8H, N-CH_2-CH_2-CH_2-CH_3); 1.61 (m, 8H, N-CH_2-CH_2-CH_2-CH_3);1.35 (sxt, $^{3}J_{H\text{-}H}$ = 7.3 Hz, 8H, N-CH_2-CH_2-CH_2-CH_3); 0.96 (t, $^{3}J_{H\text{-}H}$ = 7.3 Hz, 12H, N-CH_2-CH_2-CH_2-CH_3); ^{11}B 5.9 (bs); ^{13}C 13.8 (s, N-CH_2-CH_2-CH_2*C*H_3), 20.3 (s, N-CH_2-CH_2-*C*H_2CH_3), 24.4 (s, N-CH_2-*C*H_2-CH_2-CH_3), 59.3 (t, $^{1}J_{14N\text{-}13C}$ = 2.8 Hz, N-*C*H_2-CH_2-CH_2CH_3); ^{19}F –129.3 (bs).

The compound appears to be indefinitely stable when stored at –30 °C, but a noticeable increase in BF_4^- and other decomposition products can be observed by ^{11}B and ^{19}F NMR spectroscopy upon storage of the isolated solid or solutions at room temperature.

Elemental analysis (%): C 62.23; H 11.70; N 4.92. Calculated for $[NBu_4]_2[B_2F_6]$ ($C_{32}H_{72}B_2F_6N_2$): C 61.94; H 11.69; B 3.48; F 18.37 N, 4.51.

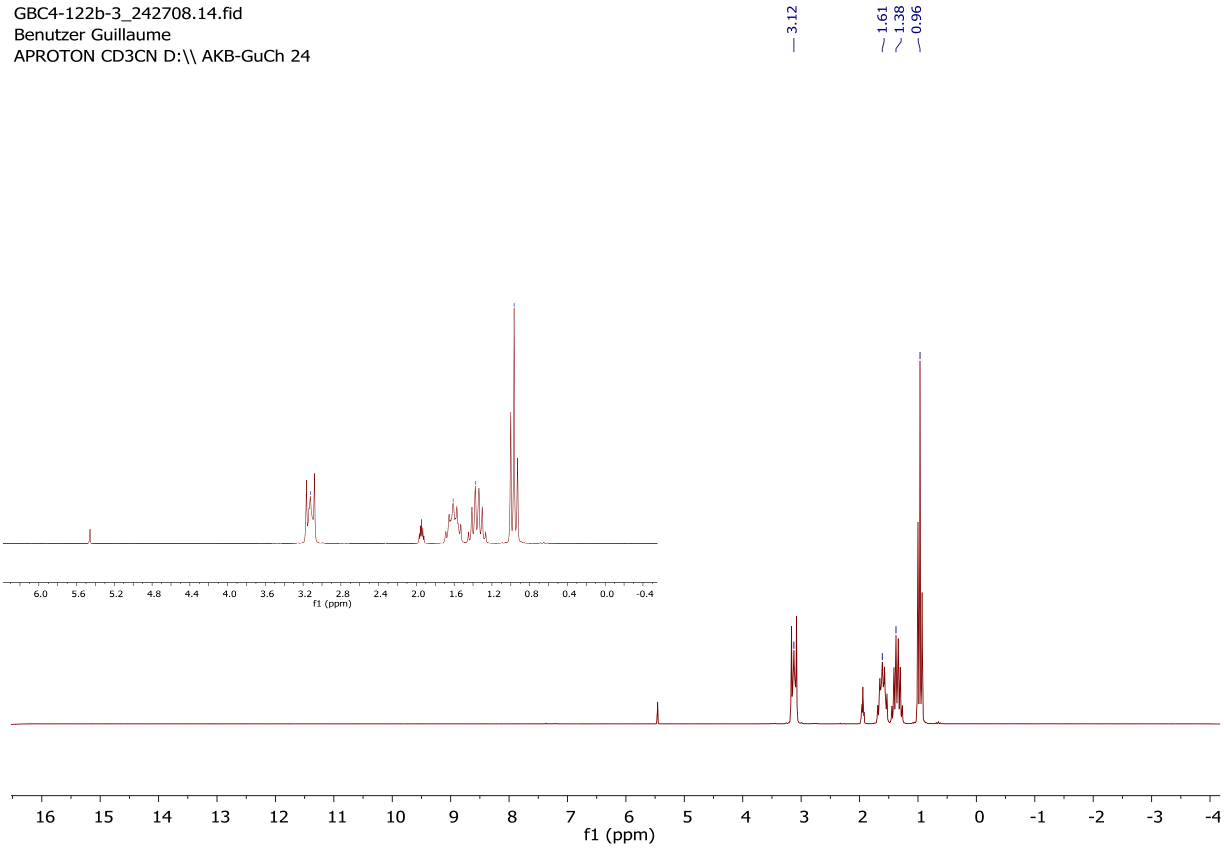

Figure S1: ^{1}H NMR (CD_3CN) spectrum of $[TBA]_2$[**1**]. The signal at *ca.* 5.5 ppm belongs to trace amounts of residual dichloromethane. Inset: zoom on the 0 to 6 ppm region.

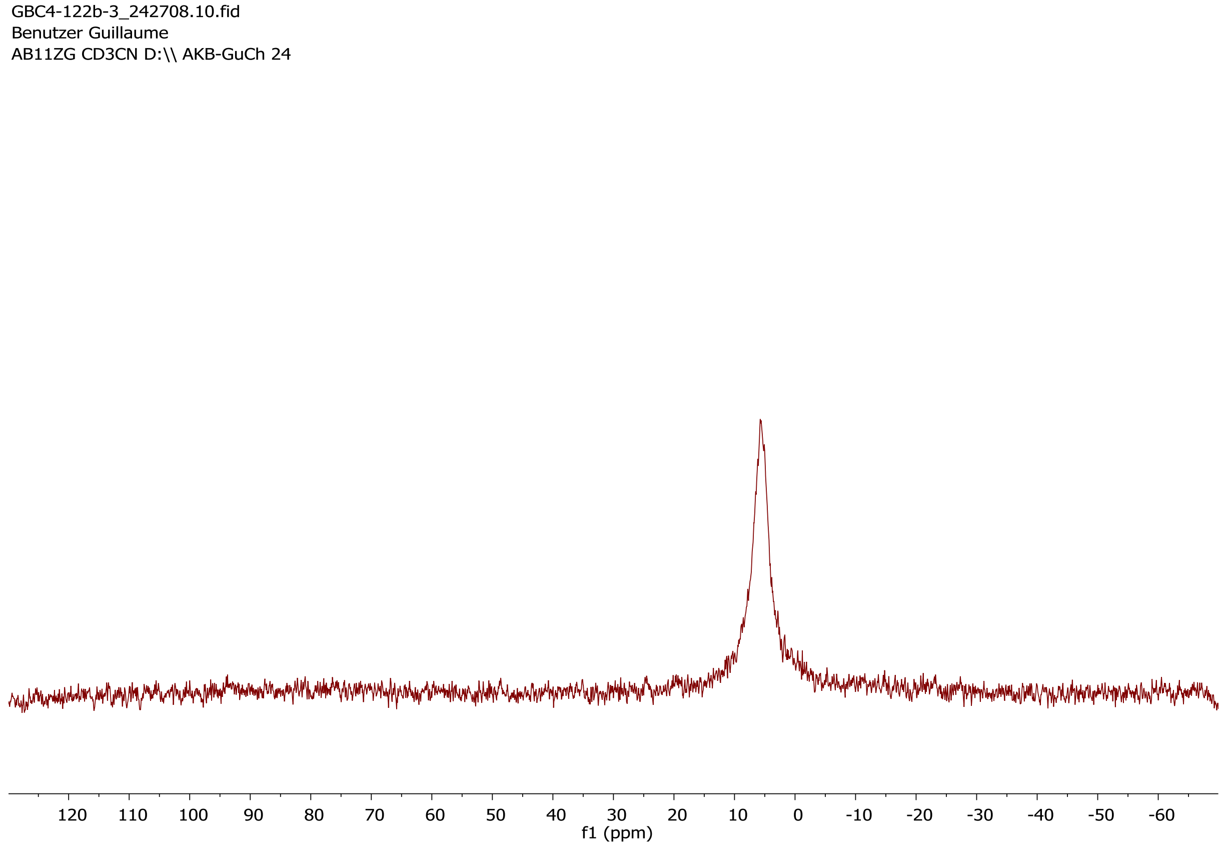

Figure S2: ^{11}B NMR (CD_3CN) spectrum of $[TBA]_2$[**1**].

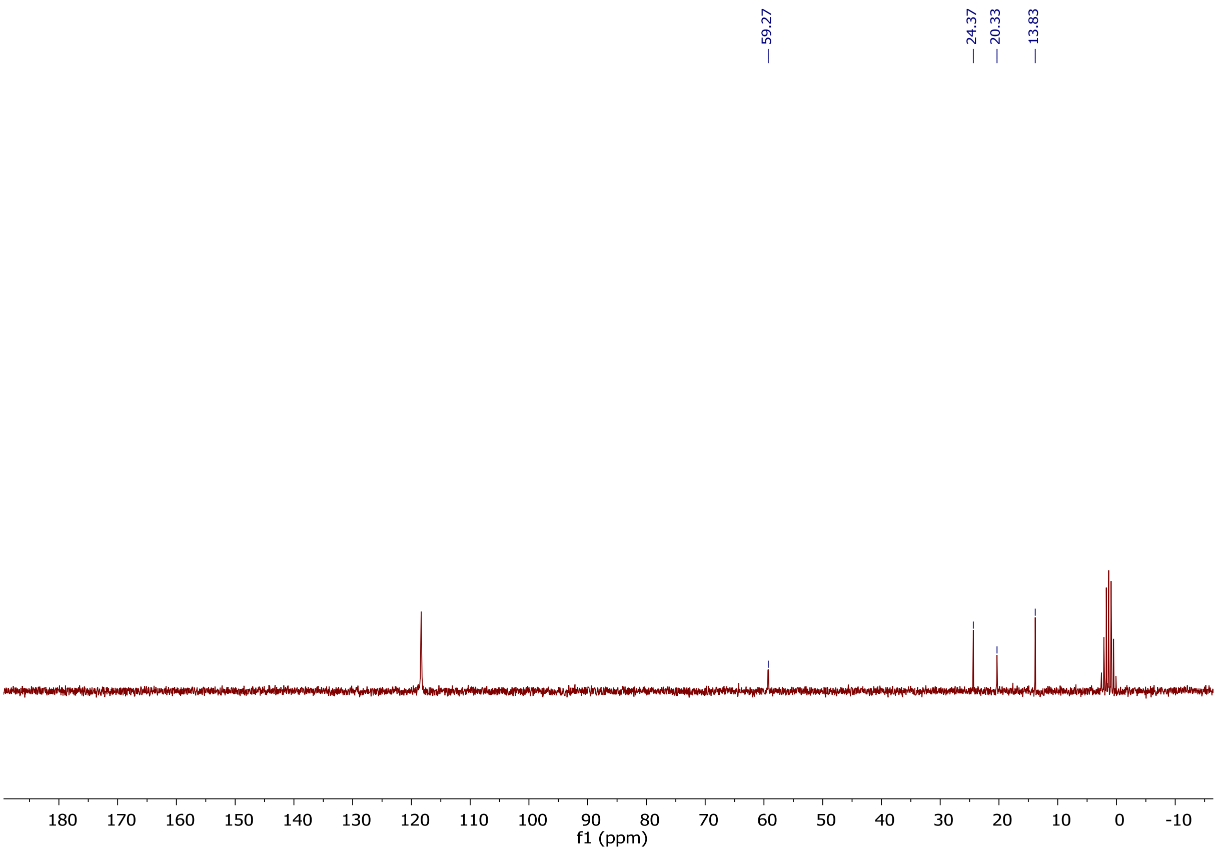

Figure S3: ^{13}C NMR (CD_3CN) spectrum of $[TBA]_2[\mathbf{1}]$.

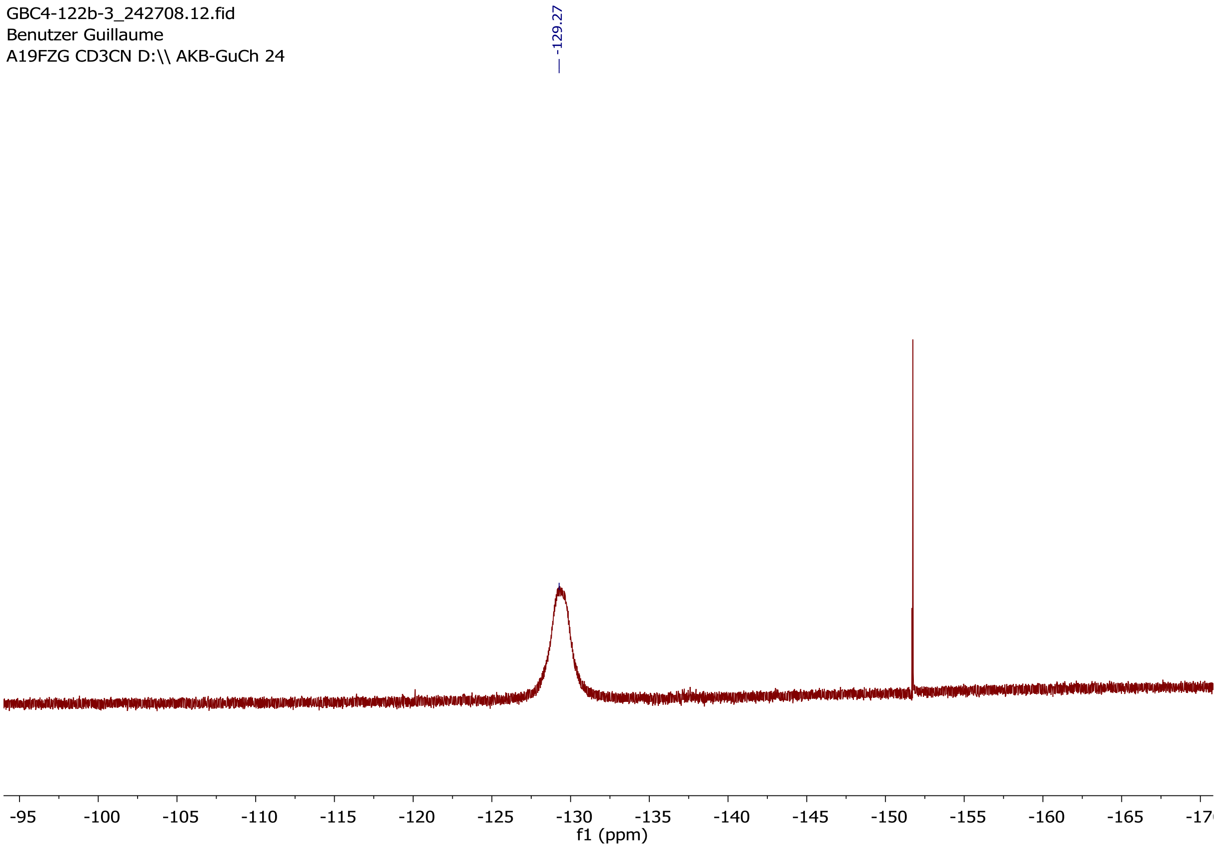

Figure S4: ^{19}F NMR (CD_3CN) spectrum of $[TBA]_2$[**1**]. The signal at –151.7 ppm belongs to small amounts of $[BF_4]^-$.

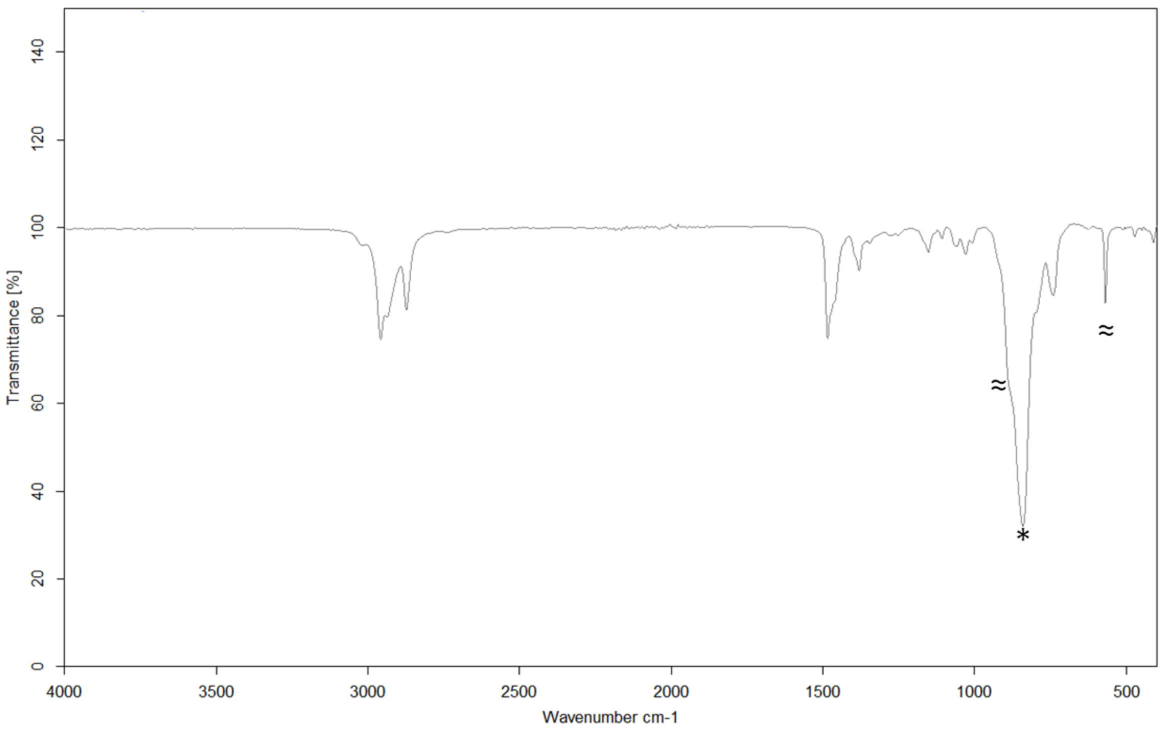

Figure S5: ATR-IR spectrum of recrystallized $[TBA]_2[\mathbf{1}]$. * denotes the vibrational band unambiguously attributable to **1** (843 cm^{-1}), while ≈ denotes bands (886 cm^{-1} and 563 cm^{-1}) which are likely attributable to **1** but might result from the [TBA] cation.

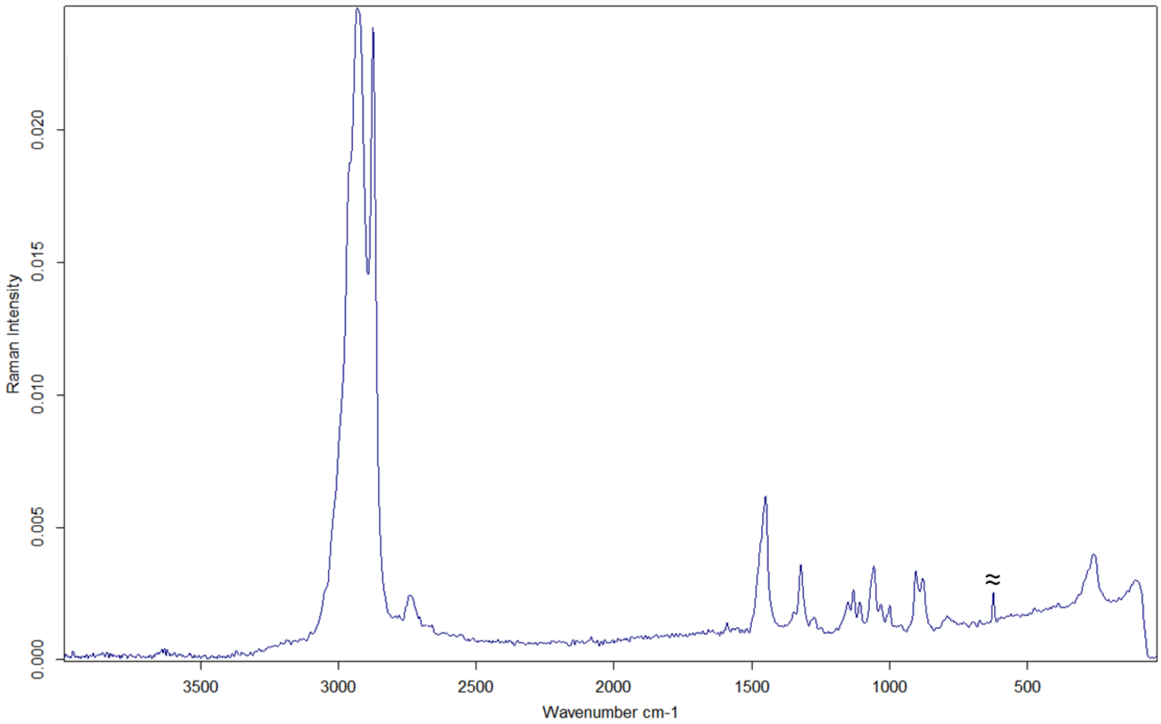

Figure S6: Raman spectrum of $[TBA]_2[\mathbf{1}]$. The spectrum is typical of a poor Raman scatterer along with signs of a fluorescence background, all of which leading to a mediocre signal. ≈ denotes a band (626 cm^{-1}) which is likely attributable to **1** but might result from the [TBA] cation. The relative weakness of the Raman signals characteristic of **1** is consistent with our computational predictions (*vide infra*).

$[PPh_4]_2[B_2Cl_6]\cdot 2CH_2Cl_2$

$[PPh_4]Cl$ (572 mg, 1.52 mmol) was placed into a reaction vessel. Dichloromethane (5-10 mL) was condensed over the solid and the solution was homogenized at room temperature, then frozen at –196 °C. B_2Cl_4 (0.75 mmol) was condensed over the frozen solution at –196 °C. The mixture was thawed at –10 °C and slowly warmed to room temperature under stirring. Copious amounts of a white precipitate formed after a few minutes of stirring. After *ca.* 30 min. of stirring, the solid was decanted and the solution removed by cannulation. The solid was rinsed with 2 x 10-20 mL of DCM and dried *in vacuo* for 15 min. $[PPh_4]_2[B_2Cl_6]$ was isolated as a white solid (667 mg; 85 mol% yield based on B_2Cl_4). Dissolution of the isolated $[PPh_4]_2[B_2Cl_6]$ in acetonitrile (leading to the decomposition of the anion) gives a ^{1}H NMR spectrum that is consistent with the presence of 1.7 to 2 molecules of DCM in the isolated $[PPh_4]_2[B_2Cl_6]$. Crystals of $[PPh_4]_2[B_2Cl_6]\cdot 2CH_2Cl_2$ formed upon reproducing the reaction conditions on a smaller scale (*ca.* 0.4 mmol B_2Cl_4 in 0.5 mL CH_2Cl_2) in a J. Young-type NMR tube. The compound crystallizes with two molecules of CH_2Cl_2 for each $[B_2Cl_6]$. Elemental analysis (%): C 55.48; H 4.14. Calculated for $[PPh_4]_2[B_2Cl_6]\cdot 2CH_2Cl_2$ ($C_{50}H_{44}B_2Cl_{10}P_2$): C, 55.45; H, 4.10; B, 2.00; Cl, 32.74; P, 5.72.

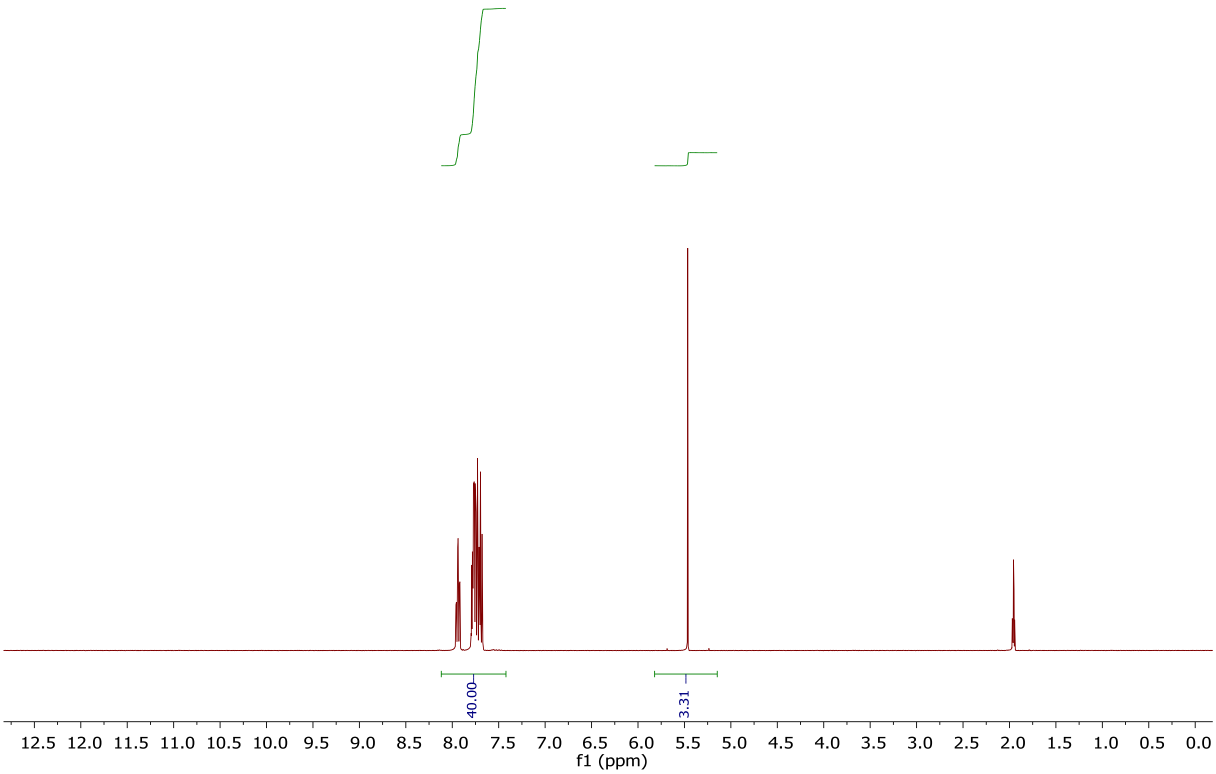

Figure S7: ^{1}H NMR (CD_3CN) spectrum of isolated $[PPh_4]_2$[**2**], showing the amount of CH_2Cl_2 (signal at *ca.* 5.5 ppm) leftover in the isolated material.

GBC4-140c-2_231403.10.fid
PPh4)2 B2Cl6 xDCM
in CD3CN
A11BZG CD3CN {D:\Topspin21} User 23

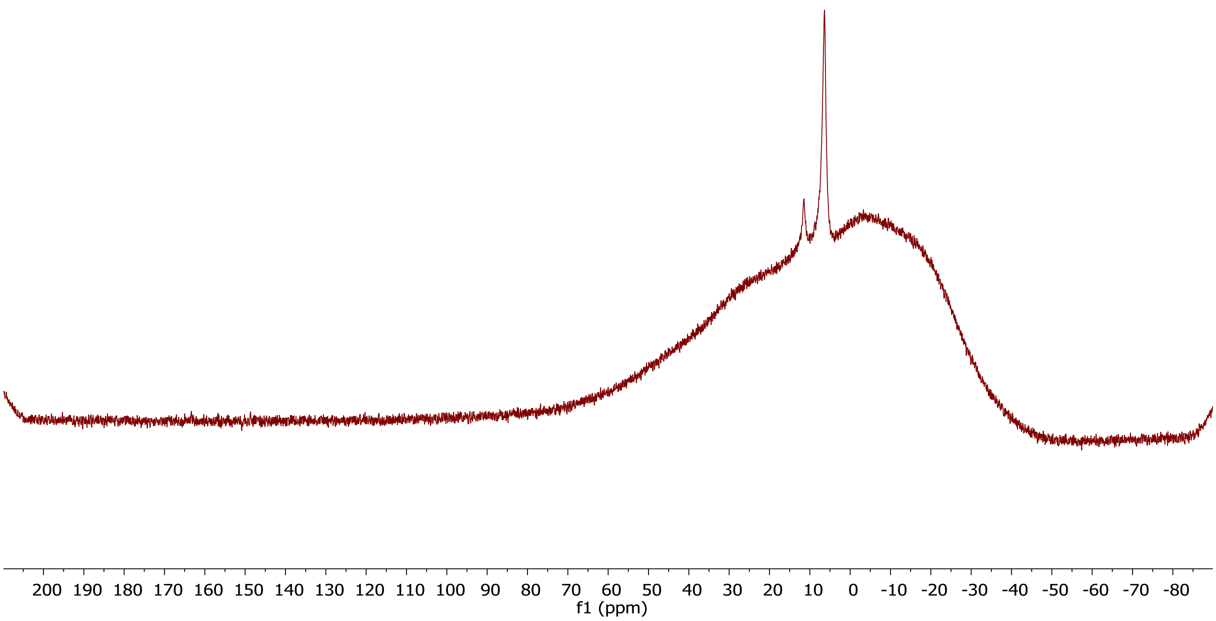

Figure S8: ^{11}B NMR (CD_3CN) spectrum of isolated $[PPh_4]_2$[**2**]. The signal at *ca.* 10 ppm is assigned to $[PPh_4]_2$[**2**] and the major signal at 6.5 to the putative $[PPh_4][Cl_3BBCl_2(NCCD_3)]$ (predicted: –3.5; 5.7 ppm, or an average of *ca.* 1 ppm). Interestingly, $[PPh_4]_2$[**2**] is the only species that crystallizes from such solutions (see crystallographic data section).

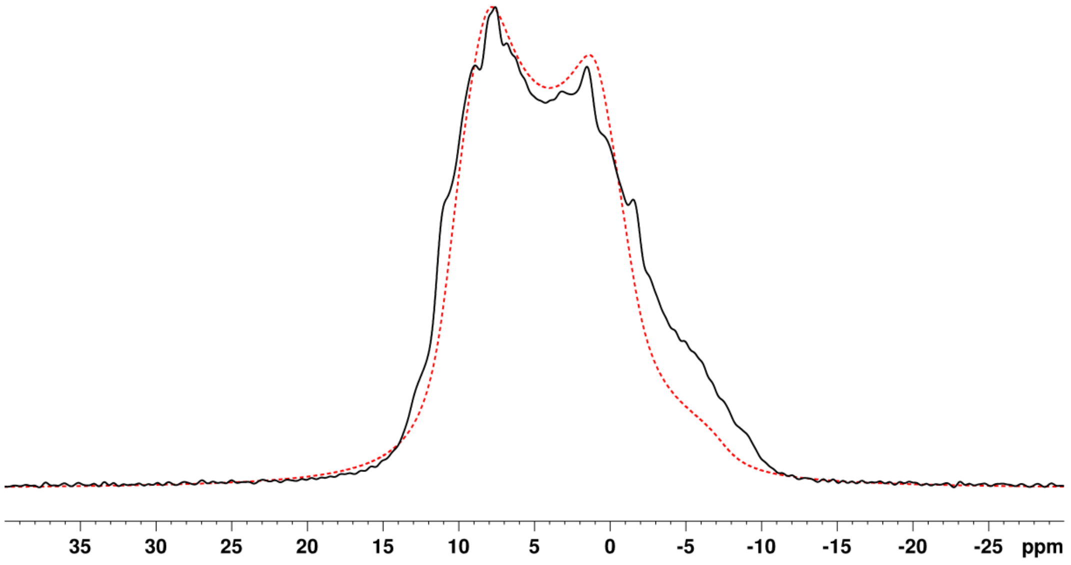

Figure S9: MAS ^{11}B NMR spectrum of isolated $[PPh_4]_2$[**2**] (solid trace) and simulated spectrum with $\delta(^{11}B)$ 12.3 ppm (dashed line).

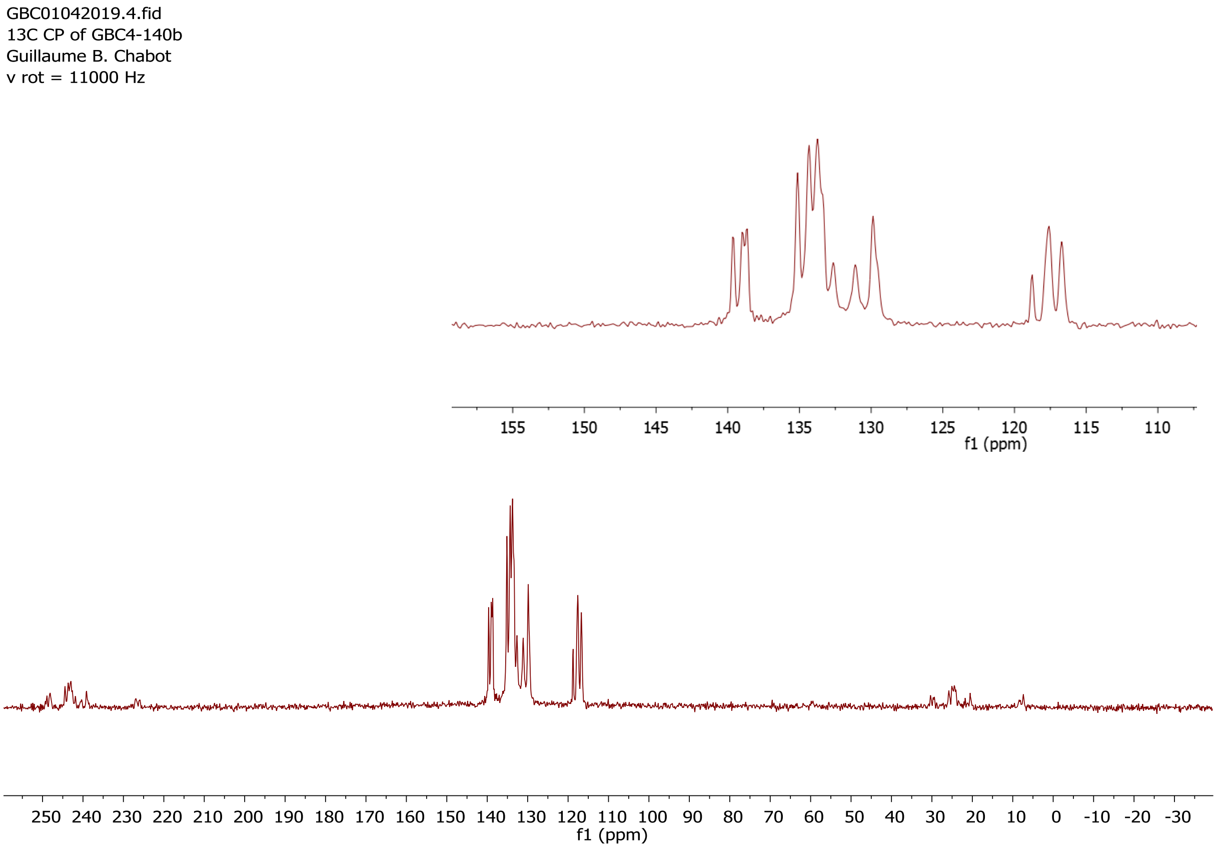

Figure S10: MAS ^{13}C NMR spectrum of isolated $[PPh_4]_2$[**2**]. Inset: zoom on the 110 to 155 ppm region.

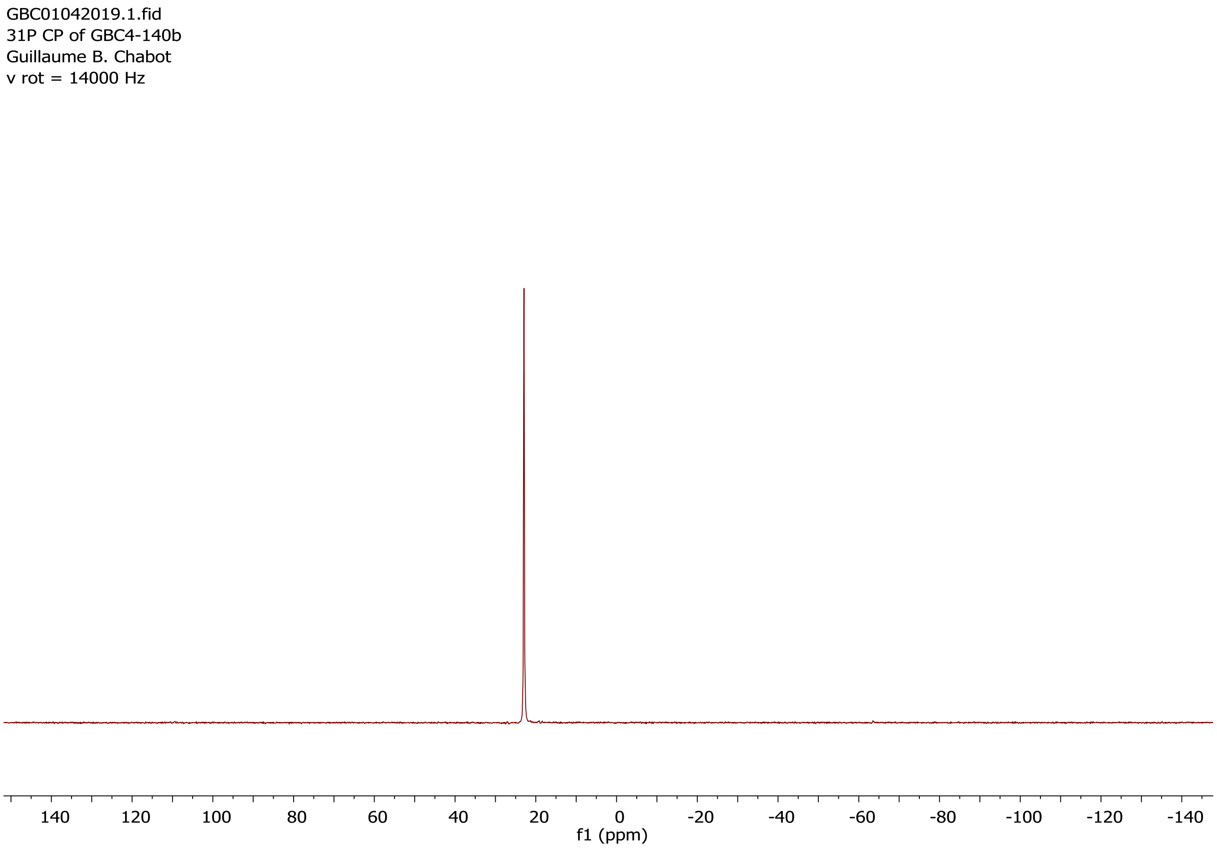

Figure S11: MAS ^{31}P NMR spectrum of isolated $[PPh_4]_2$[**2**].

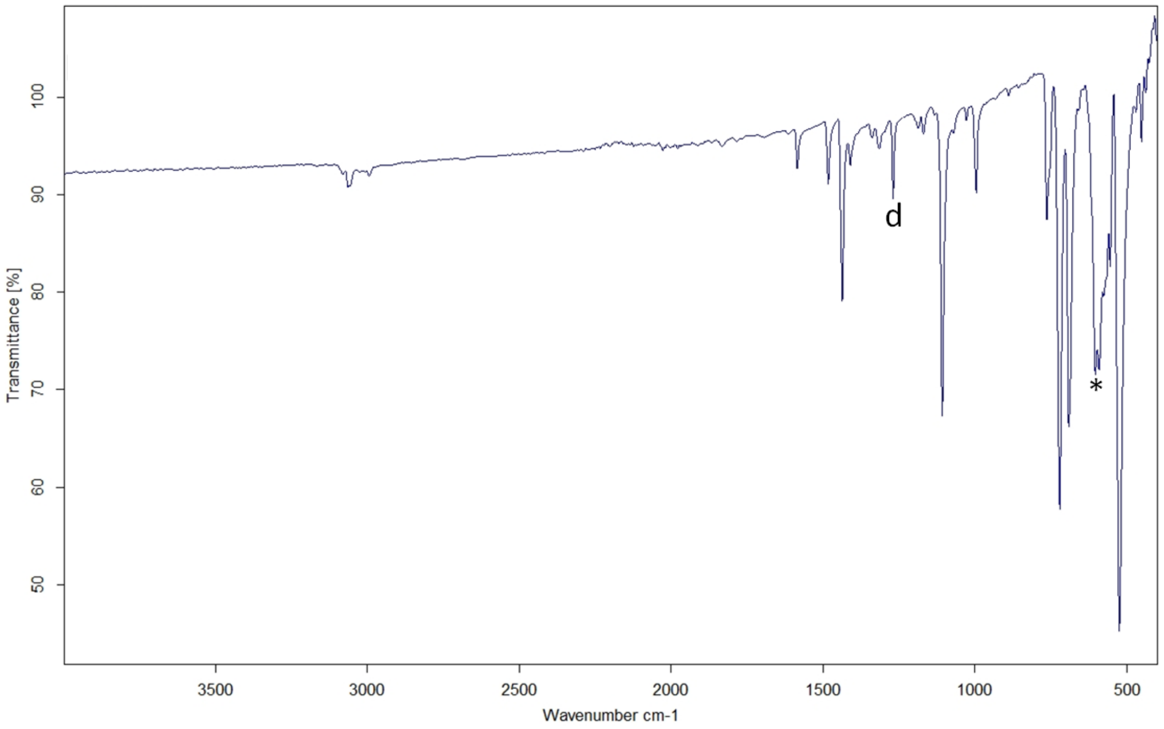

Figure S12: ATR-IR spectrum of $[PPh_4]_2$[**2**]·$2CH_2Cl_2$ * denotes the vibrational band unambiguously attributable to the [**2**] dianion (complex feature at 580 cm^{-1}), while d denotes a band (1266 cm^{-1}) likely attributable to cocrystallized CH_2Cl_2 (CH_2 wag 1265 cm^{-1}).[7]

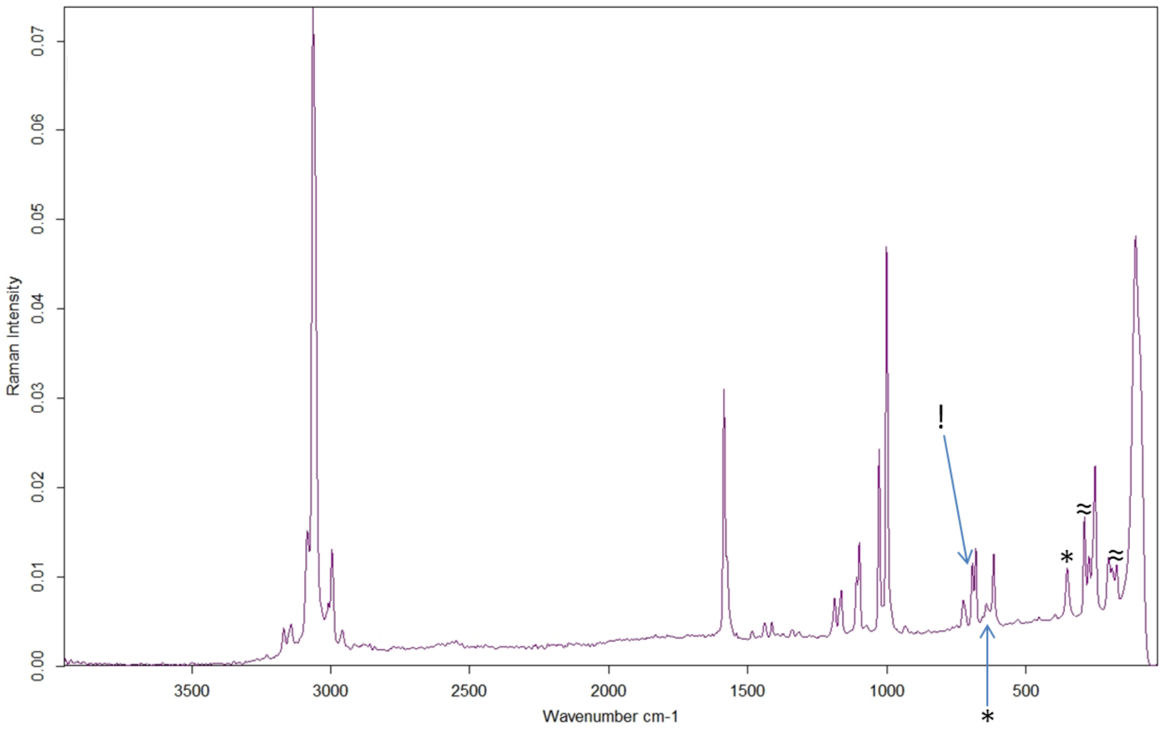

Figure S13: Raman spectrum of $[PPh_4]_2$[**2**]·$2CH_2Cl_2$ * denotes the vibrational bands unambiguously attributable to the [**2**] dianion (641 and 354 cm^{-1}). ≈ denotes bands (275 and 175 cm^{-1}) that are most likely attributable to [**2**] but overlap with groups of $[PPh_4]$ bands. !

denotes a band (692 cm^{-1}) that may belong to cocrystallized CH_2Cl_2 (CCl_2 sym. stretch 717 cm^{-1}),[7] $[BCl_4]^-$ impurities (670 and 707 cm^{-1})[8] or to the $[PPh_4]$ cation.

$[PPh_4]_2[B_2Br_6]{\cdot}xCH_2Cl_2$

$[PPh_4]Br$ (797 mg, 1.90 mmol) was placed into a reaction vessel. Dichloromethane (5-10 mL) was condensed over the solid and the solution was homogenized at room temperature, then frozen at –196 °C. B_2Br_4 (320 mg; 0.94 mmol) was condensed over the frozen solution at –196 °C. The mixture was thawed at –20 °C and slowly warmed up to room temperature under stirring. Copious amounts of a white precipitate formed after a few minutes of stirring. After *ca.* 90 min. of stirring, the solid was decanted and the solution removed by cannulation. The solid was rinsed with twice with 10-20 mL of CH_2Cl_2 and the solid was dried *in vacuo* for 90 min. $[PPh_4]_2[B_2Br_6]$ was isolated as a white solid (892 mg; 75 mol% yield based on B_2Br_4). Dissolution of the isolated $[PPh_4]_2[B_2Br_6]$ in acetonitrile (leading to the decomposition of the anion) suggests that only *ca.* one molecule of CH_2Cl_2 is still present after the isolation of the bulk $[PPh_4]_2[B_2Br_6]$. Crystals of $[PPh_4]_2[B_2Br_6]$ formed upon treating a B_2Br_4 (*ca.* 0.5 mmol) solution in CH_2Cl_2 (*ca.* 0.5 mL) with $[PPh_4]Br$ in a J. Young-type NMR tube. The compound crystallizes with two molecules of CH_2Cl_2 for each $[B_2Br_6]$. Elemental analysis (%): C 46.37; H 3.32. Calculated for $[PPh_4]_2[B_2Br_6]{\cdot}CH_2Cl_2$ ($C_{49}H_{42}B_2Br_6Cl_2P_2$): C, 46.53; H, 3.35; B, 1.71; Br, 37.91; Cl, 5.61; P, 4.90.

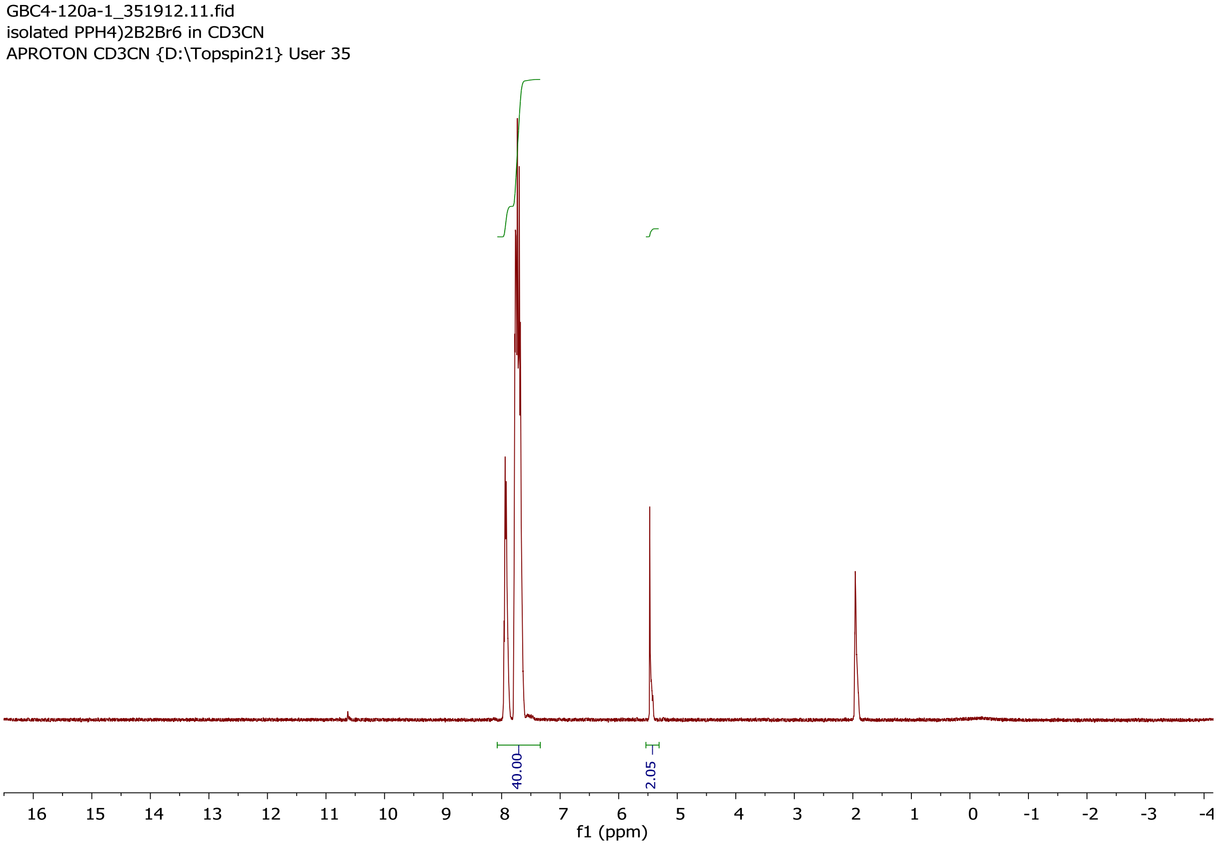

Figure S14: ^{1}H NMR (CD_3CN) spectrum of isolated $[PPh_4]_2$[**3**], showing the amount of CH_2Cl_2 (signal at *ca* 5.5 ppm) leftover in the isolated material.

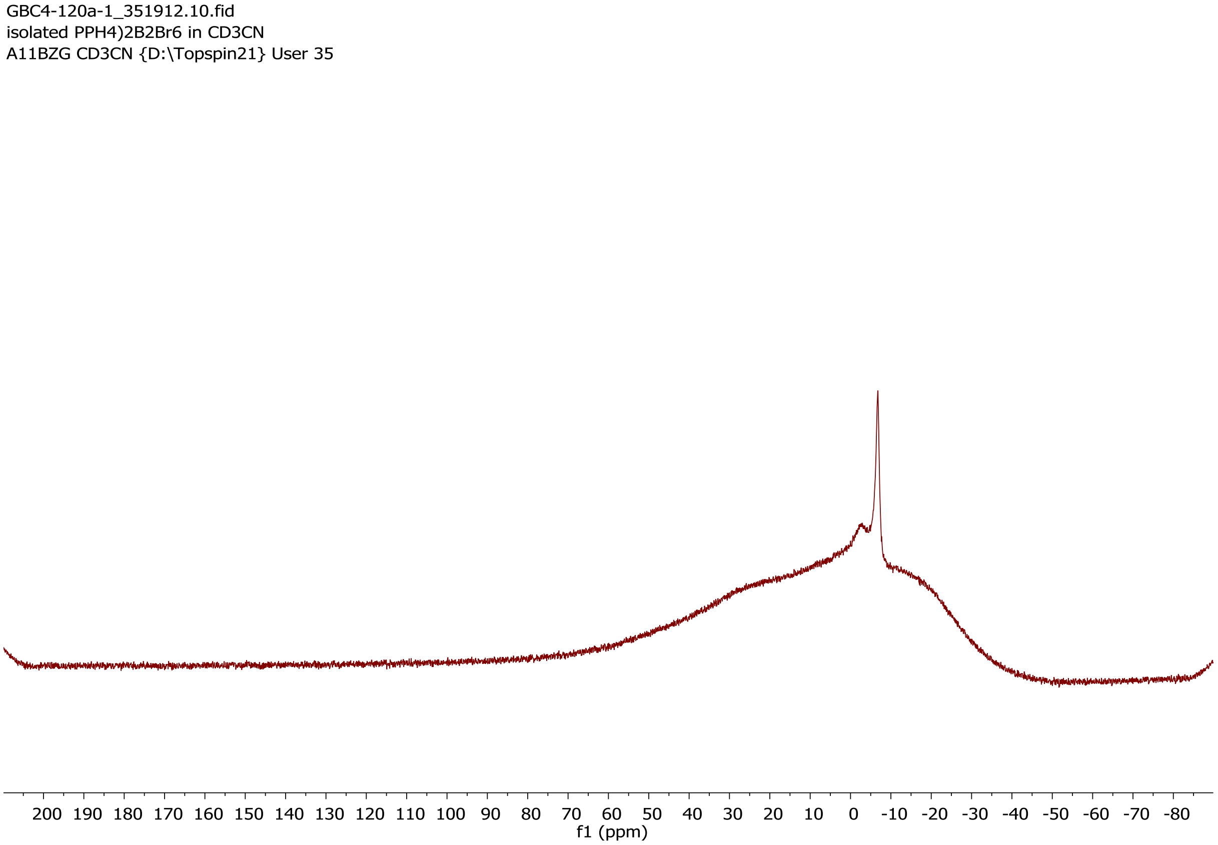

Figure S15: ^{11}B NMR (CD_3CN) spectrum of isolated $[PPh_4]_2$[**3**]. The signal at *ca* –3 ppm is tentatively assigned to $[Br_3BBBr_2(NCCD_3)]$, assuming a rapid averaging of the expected two inequivalent boron environments, and the major signal at –7 ppm to the putative $[(CD_3CN)Br_2BBBr_2(NCCD_3)]$. The existence of the former was attested by single-crystal X-ray crystallography (see crystallographic data section).

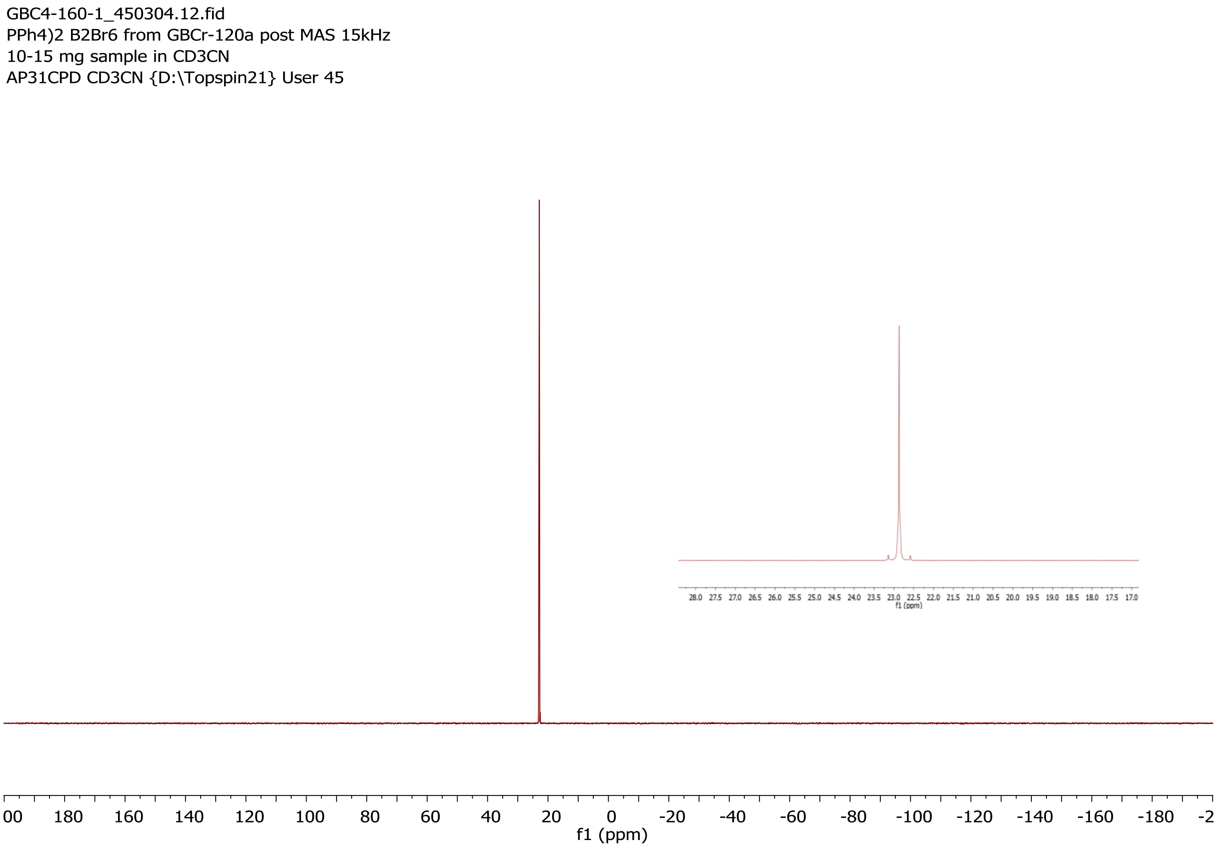

Figure S16: $^{31}P\{^{1}H\}$ NMR (CD_3CN) spectrum of isolated $[PPh_4]_2[\mathbf{3}]^{\cdot}xCH_2Cl_2$. Inset: zoom on the 18 to 28 ppm region.

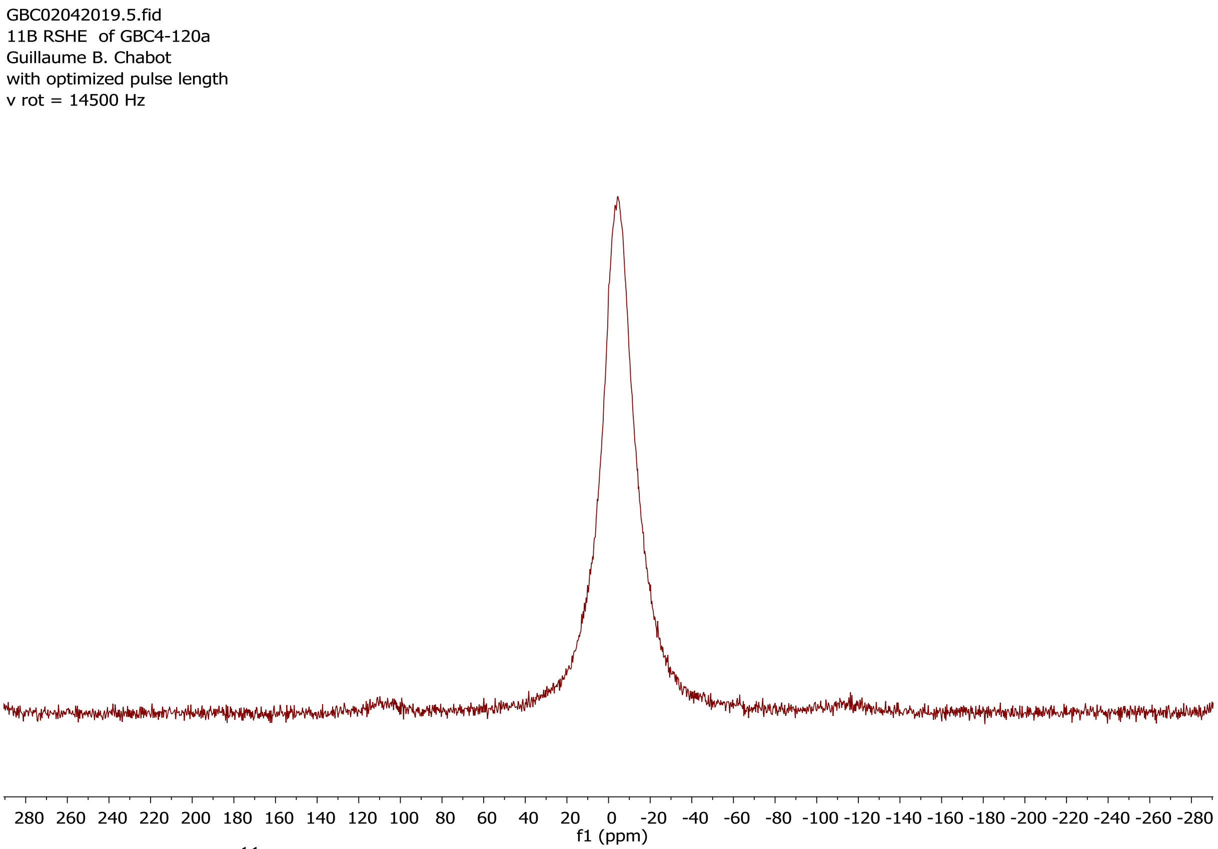

Figure S17: MAS ^{11}B NMR spectrum of isolated $[PPh_4]_2[\mathbf{3}]^{\cdot}xCH_2Cl_2$.

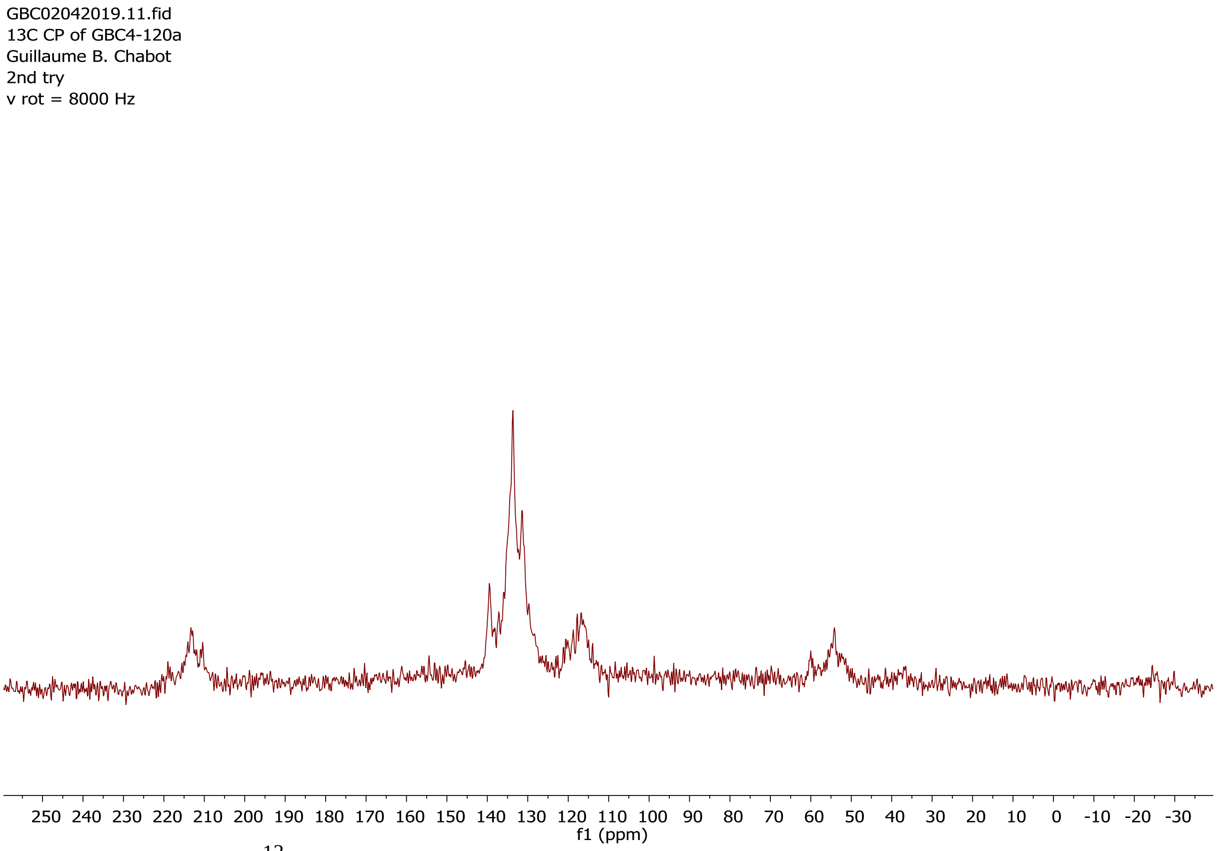

Figure S18: MAS ^{13}C NMR spectrum of isolated $[PPh_4]_2$[**3**]·xCH_2Cl_2. The apparent low resolution of the spectrum is likely due to the diversity of carbon environments (see MAS ^{31}P NMR) and possibly to the partial liquefaction of the sample under the rapid rotation of the MAS NMR measurement. The signals are nonetheless consistent with the higher-resolution spectrum observed for $[PPh_4]_2$[**2**].

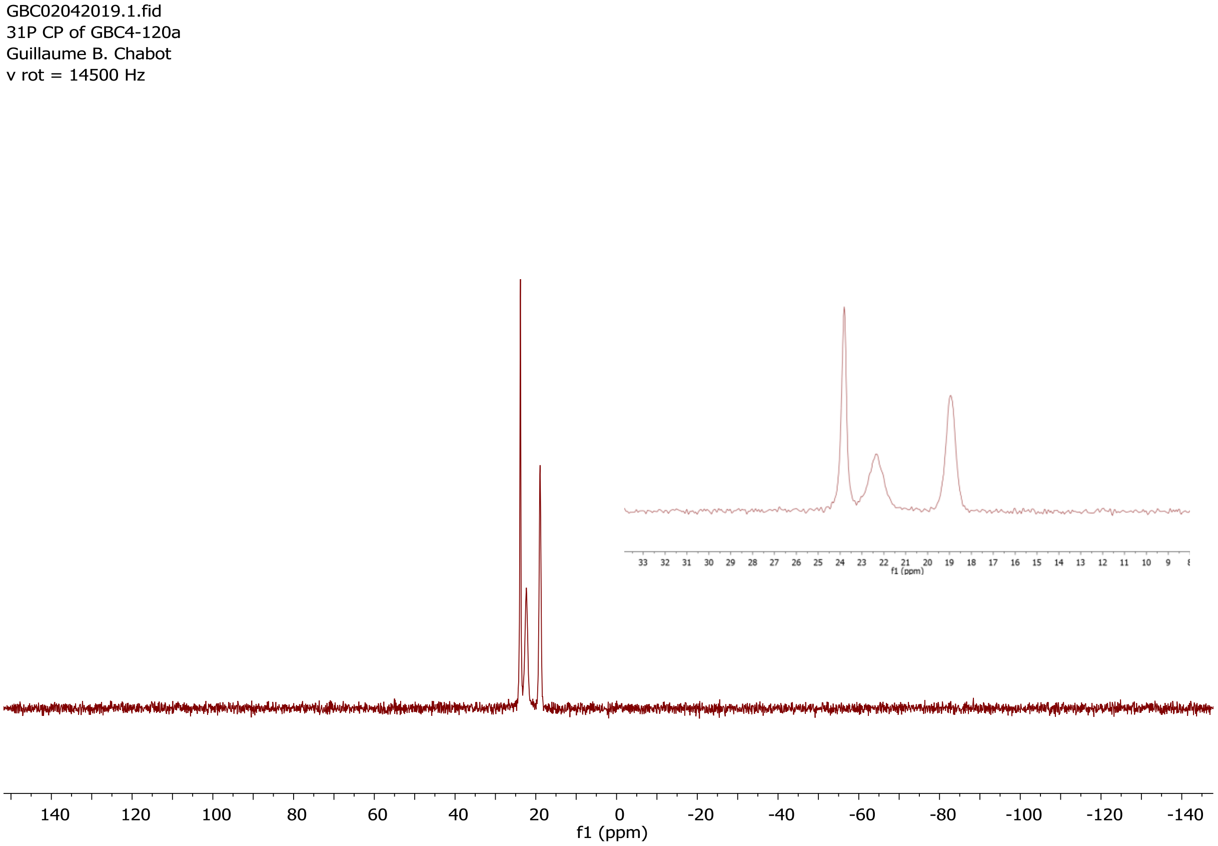

Figure S19: MAS ^{31}P NMR spectrum of isolated $[PPh_4]_2[\mathbf{3}]^{\cdot}xCH_2Cl_2$. The complexity of the signal is consistent with at least three distinct phosphorus environments, in agreement with the observed loss of DCM from the known crystalline phase $[PPh_4]_2[\mathbf{3}]^{\cdot}2CH_2Cl_2$. Solution-phase NMR spectroscopy in CD_3CN solution on the sample post-MAS NMR indicated that no decomposition of the $[PPh_4]^+$ cation occured (see Figure S16). Inset: zoom on the 9 to 33 ppm region.

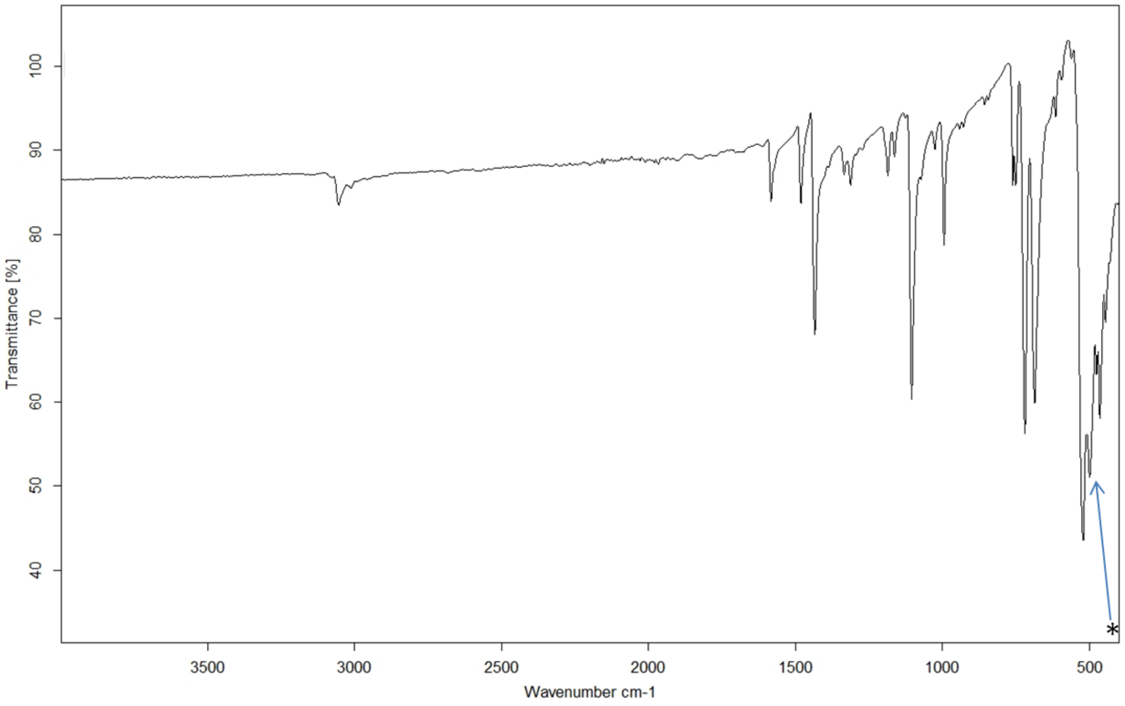

Figure S20: ATR-IR spectrum of $[PPh_4]_2[\mathbf{3}]^{\cdot}xCH_2Cl_2$ * denotes the vibrational band that is very likely attributable to the **3** dianion (498 cm^{-1}).

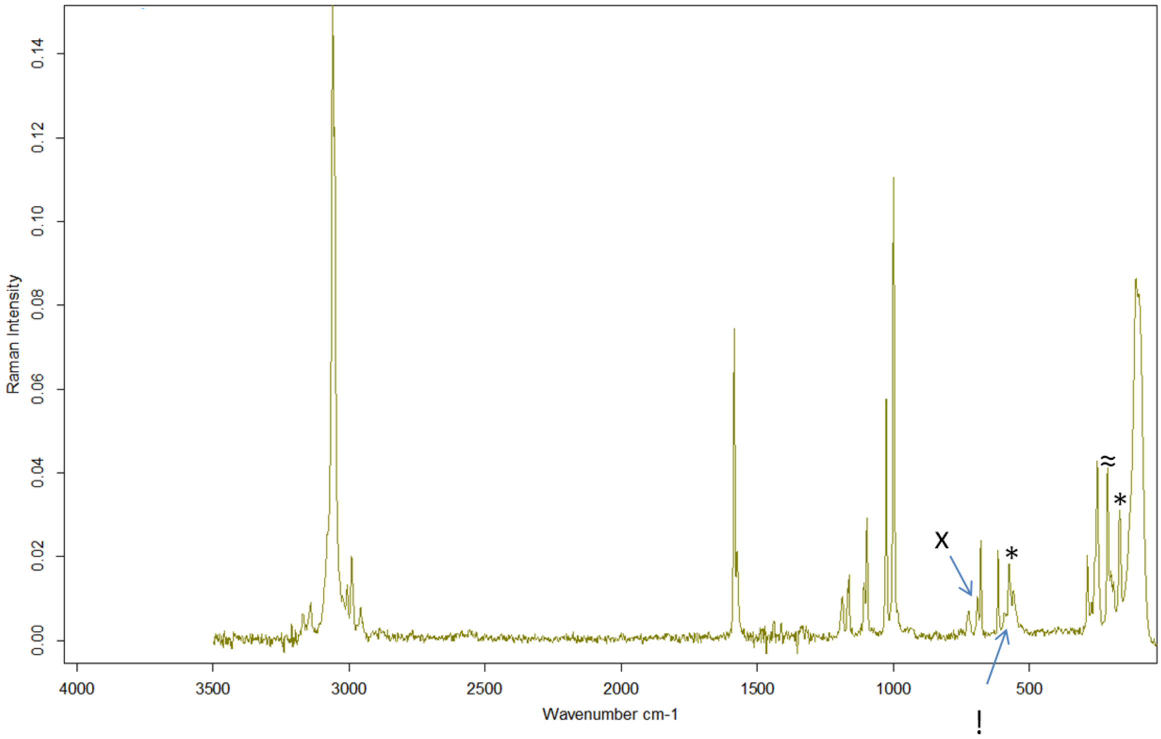

Figure S21: Raman spectrum of $[PPh_4]_2[\mathbf{3}]\cdot xCH_2Cl_2$. * denotes vibrational bands (576;560 and 170cm^{-1}) that can be unambiguously attributed to the **3** dianion. The multiplicity of the complex feature centered at 575 cm^{-1} is likely in part due to the diversity of phases present in the sample (probably a continuum between $[PPh_4]_2[\mathbf{3}]\cdot 2CH_2Cl_2$ and $[PPh_4]_2[\mathbf{3}]$), which is strongly supported by MAS-NMR, solution ^{1}H NMR spectroscopy and single-crystal X-ray crystallography. ≈ denotes a band (214 cm^{-1}) that is likely attributable to **3** but could be due to the $[PPh_4]^+$ cation. ! denotes a signal potentially due to small impurities of BBr_4^-. x denotes a band (692 cm^{-1}) that may belong to cocrystallized CH_2Cl_2 (CCl_2 sym. stretch 717 cm^{-1})[7] or to the $[PPh_4]$ cation.

$[PPh_4]_2[B_2I_6]\cdot xCH_2Cl_2$

$[PPh_4]I$ (55 mg; 0.12 mmol) was dissolved in a minimum amount of DCM. The resulting solution was added onto a *ca.* 2 mL toluene suspension of B_2I_4 (29 mg; 0.55 mmol). DCM was added dropwise until a pale yellow clear solution was obtained. A few drops of toluene were added and the solution was stored at –30 °C for a few hours, at which point crystals started to form. After several days at that temperature, $[PPh_4]_2[B_2I_6]$ was obtained (36 mg; 45 mol% yield based on B_2I_4) as a colorless crystalline material. Depending on the conditions, crystals of $[PPh_4]_2[B_2I_6]\cdot 2CH_2Cl_2$ can also be obtained. Larger amounts of more variable composition material ("bulk" material) can be obtained by adding a saturated solution of $[PPh_4]I$ in CH_2Cl_2 (2 equiv) to a saturated solution of B_2I_4 in toluene (1 equiv). A colorless precipitate forms which consists mostly of $[PPh_4][\mathbf{4}]$ with varying amounts of cocrystallized solvent (CH_2Cl_2) and likely small amounts of dissociated **4** (a mixture of $[PPh_4]I$ and putative $[PPh_4][B_2I_5]$). MAS-NMR spectra of microcrystalline samples indeed hint at the possible coprecipitation of small amounts of the putative $[PPh_4][B_2I_5]$ and $[PPh_4]I$. Elemental analysis (%): C 39.76; H 2.74. Calculated for $[PPh_4]_2[B_2I_6]$ ($C_{48}H_{40}B_2I_6P_2$): C, 39.44; H, 2.76; B, 1.48; I, 52.09; Cl, P, 4.24.

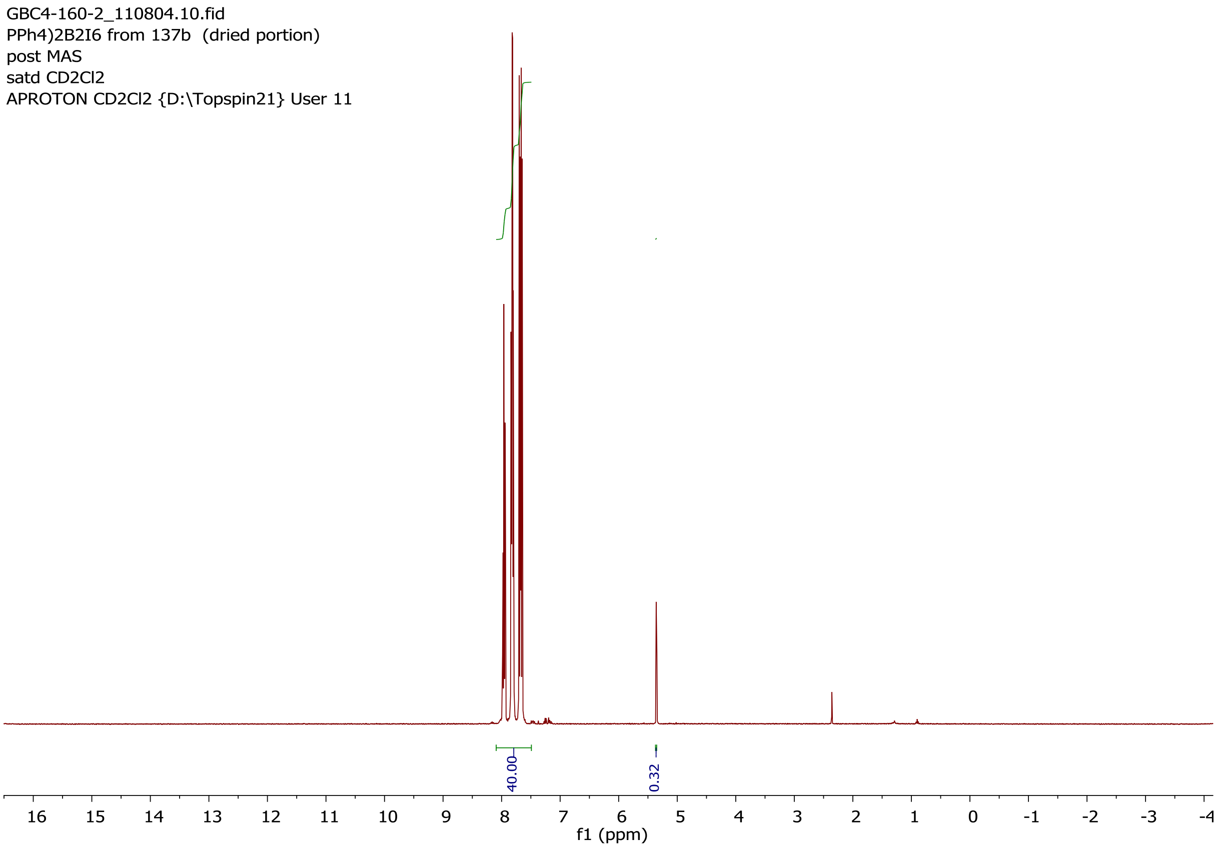

Figure S22: ^{1}H NMR (CD_2Cl_2) spectrum of isolated bulk $[PPh_4]_2$[**4**], showing the amount of CH_2Cl_2 (signal at *ca* 5.5 ppm) leftover in the isolated bulk material. The signals between 0 and 3 ppm belong to small amounts of residual toluene and pentane.

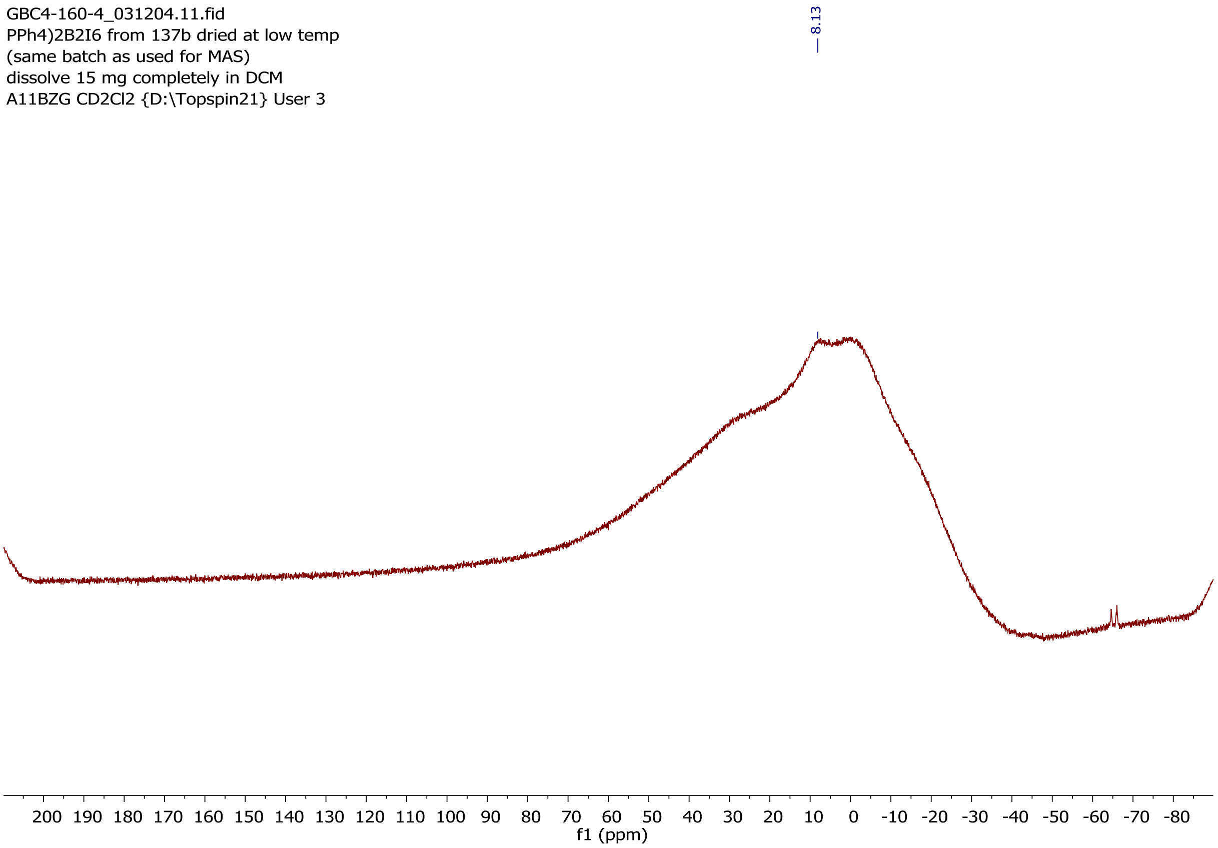

Figure S23: ^{11}B NMR (CH_2Cl_2, unlocked) spectrum of isolated bulk $[PPh_4]_2$[**4**]. The signal at *ca.* –60 ppm belongs to small amounts of an unidentified decomposition product. The main, very broad signal at *ca.* 8 ppm is assigned to the putative $[B_2I_5]^-$ species, in which the two different boron environments would average out in solution. Solutions from which $[PPh_4]_2$[**4**]$\cdot xCH_2Cl_2$ crystals are consistently obtained (as confirmed by single-crystal X-ray diffraction) systematically produce ^{11}B NMR spectra such as this one (see further notes on solution NMR below).

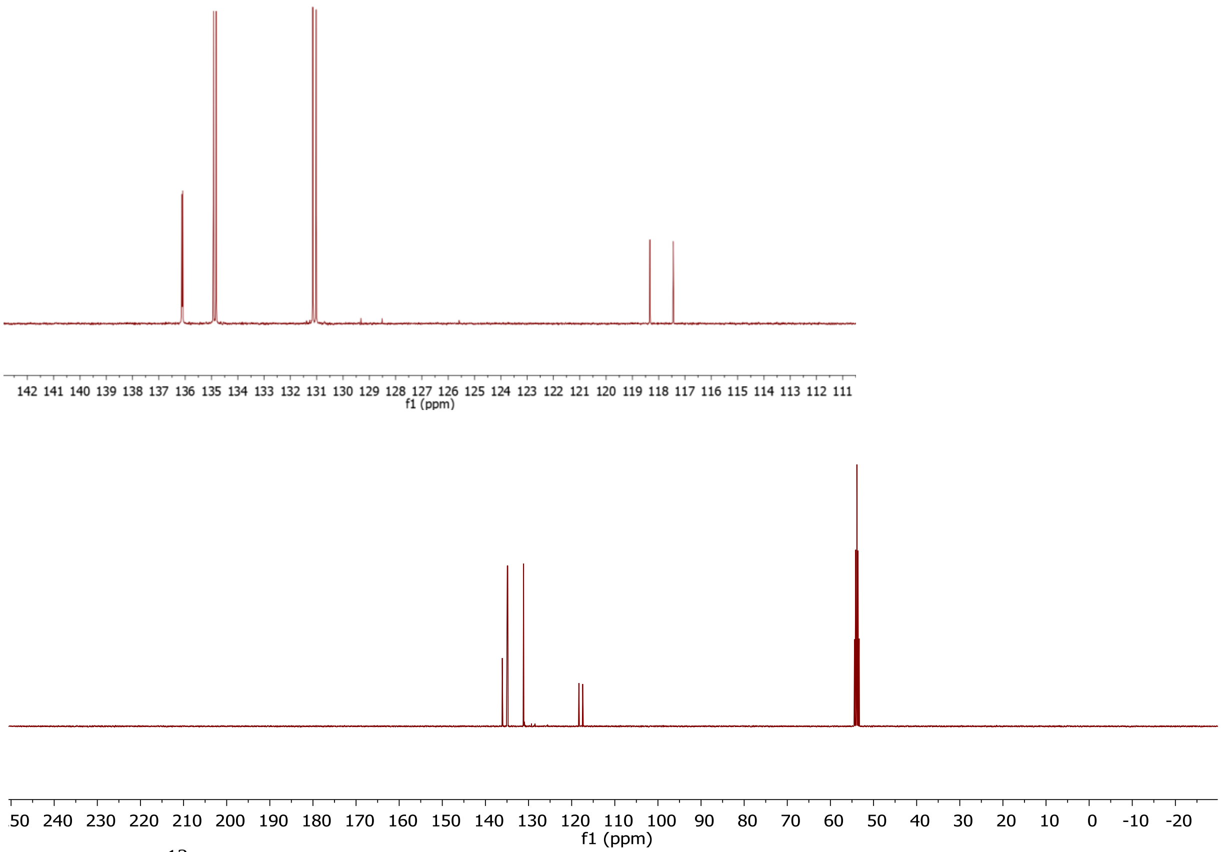

Figure S24: ^{13}C NMR (CD_2Cl_2) spectrum of isolated bulk $[PPh_4]_2$[**3**]. Inset: zoom on the aromatics region of the spectrum.

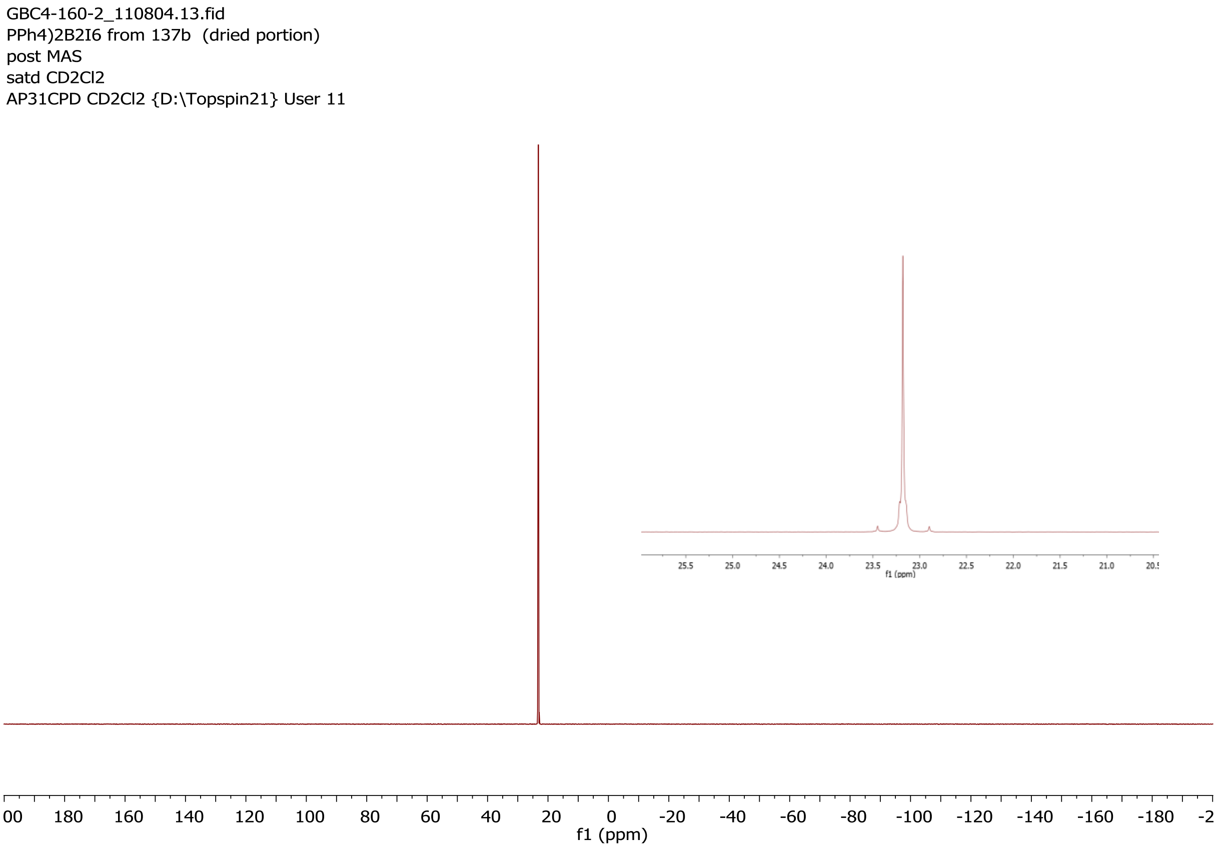

Figure S25: ^{31}P NMR (CD_2Cl_2) spectrum of isolated bulk $[PPh_4]_2[\mathbf{3}]$. Inset: zoom on the 20 to 25 ppm region of the spectrum.

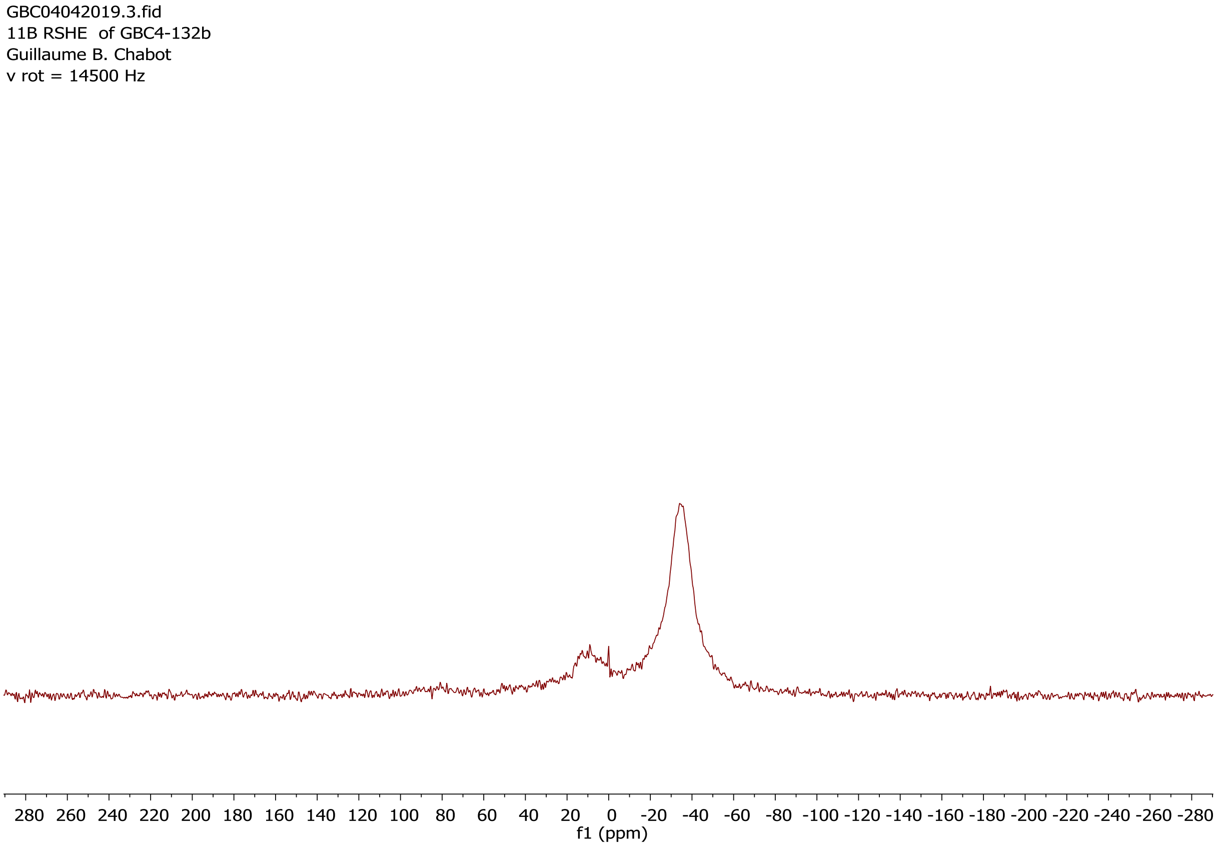

Figure S26: MAS ^{11}B NMR of isolated $[PPh_4]_2[\mathbf{4}]^{\cdot}xCH_2Cl_2$. The smaller signal at *ca.* 10 ppm is consistent with small amounts of the main species observed in solution, which we assign to $[B_2I_5]^-$. The absence of two very different boron environments for that putative species indicates either a fluidization of the sample under the high pressure conditions of the MAS NMR measurement, allowing for dynamic processes, the broadness of the expected $-BI_2$ signal that would render it hard to detect or, instead, that the structure of the putative $[B_2I_5]^-$ anion is symmetrical in the solid state (bridging fifth I^- moiety). Nevertheless, the spectrum indicates that small amounts of a second species cocrystallizes along with the main species in bulk syntheses of $[PPh_4]_2[\mathbf{4}]$, which can in part explain the diversity of ^{31}P environments observed by MAS ^{31}P NMR spectroscopy.

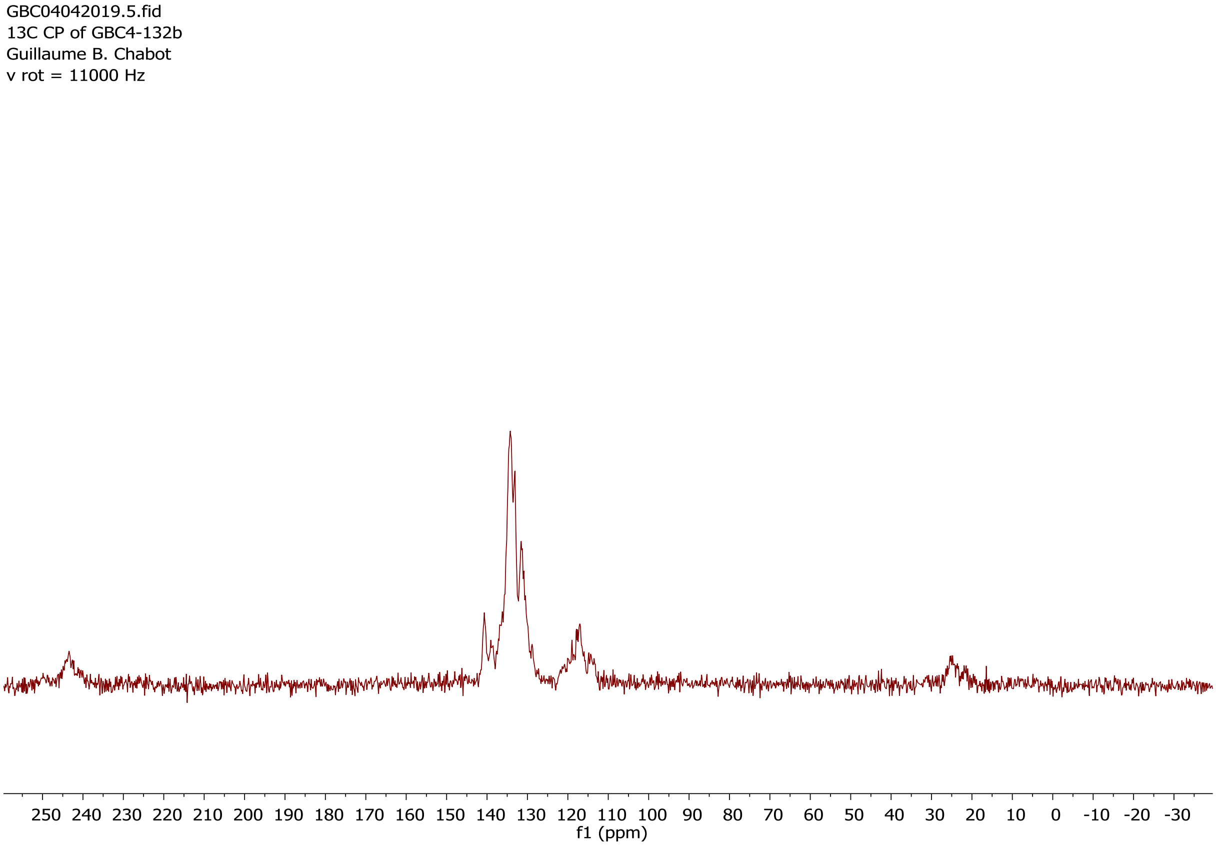

Figure S27: MAS ^{13}C NMR spectrum of isolated $[PPh_4]_2[\mathbf{4}]^{\cdot}xCH_2Cl_2$. The apparent low-resolution of the spectrum is likely due to the diversity of carbon environments for each signal (see MAS ^{31}P NMR spectrum) and possibly to the partial liquefaction of the sample under the rapid rotation conditions of the MAS NMR measurement. The signals are nonetheless consistent with the higher-resolution spectrum observed for $[PPh_4]_2[\mathbf{2}]$.

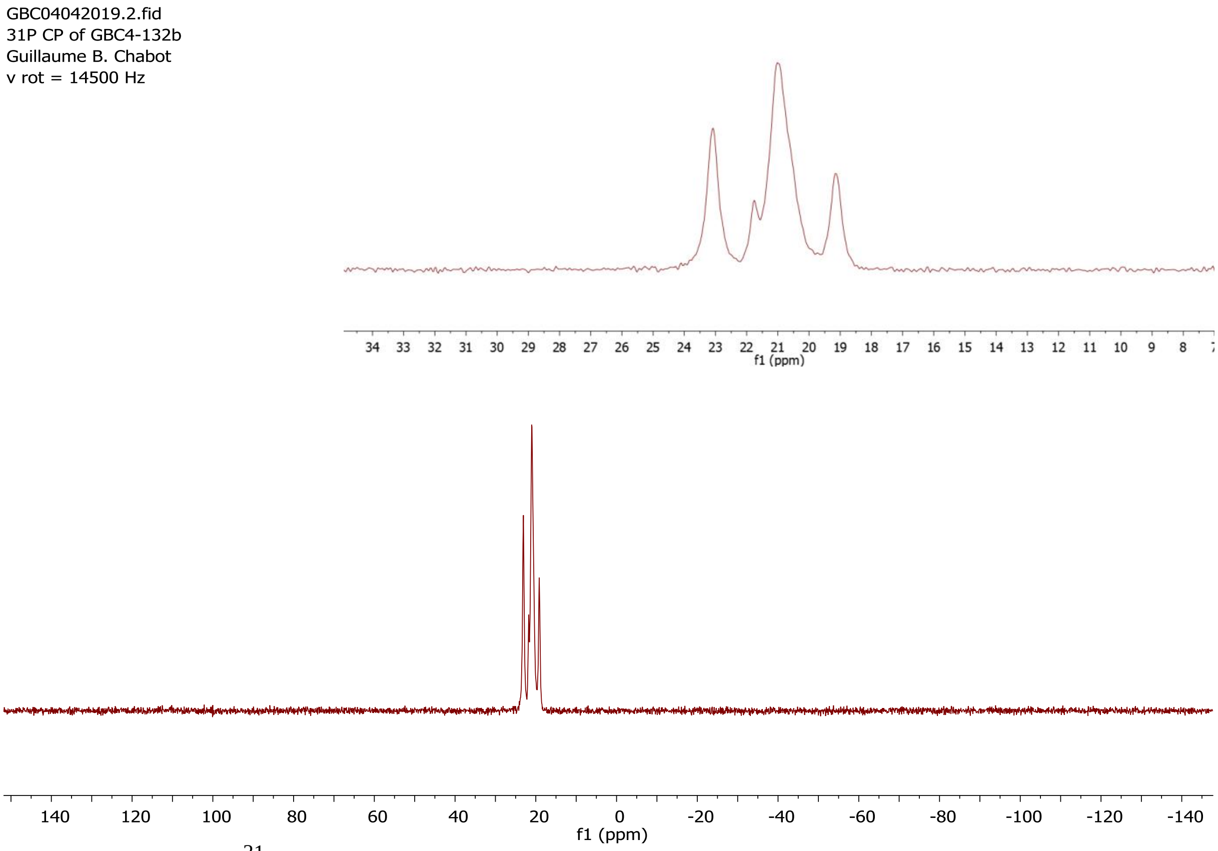

Figure S28: MAS ^{31}P NMR spectrum of isolated $[PPh_4]_2$[**4**]·xCH_2Cl_2. Inset: zoom on the 8 to 34 ppm region. The complexity of the spectrum is consistent with at least four distinct phosphorus environments, in agreement with the crystal phase variety observed by X-ray diffraction (at least two solvates). Solution phase NMR in CD_2Cl_2 solution on the sample post-MAS NMR indicates that no decomposition of the $[PPh_4]^+$ cation occured. Inset: zoom on the 7 to 34 ppm region.

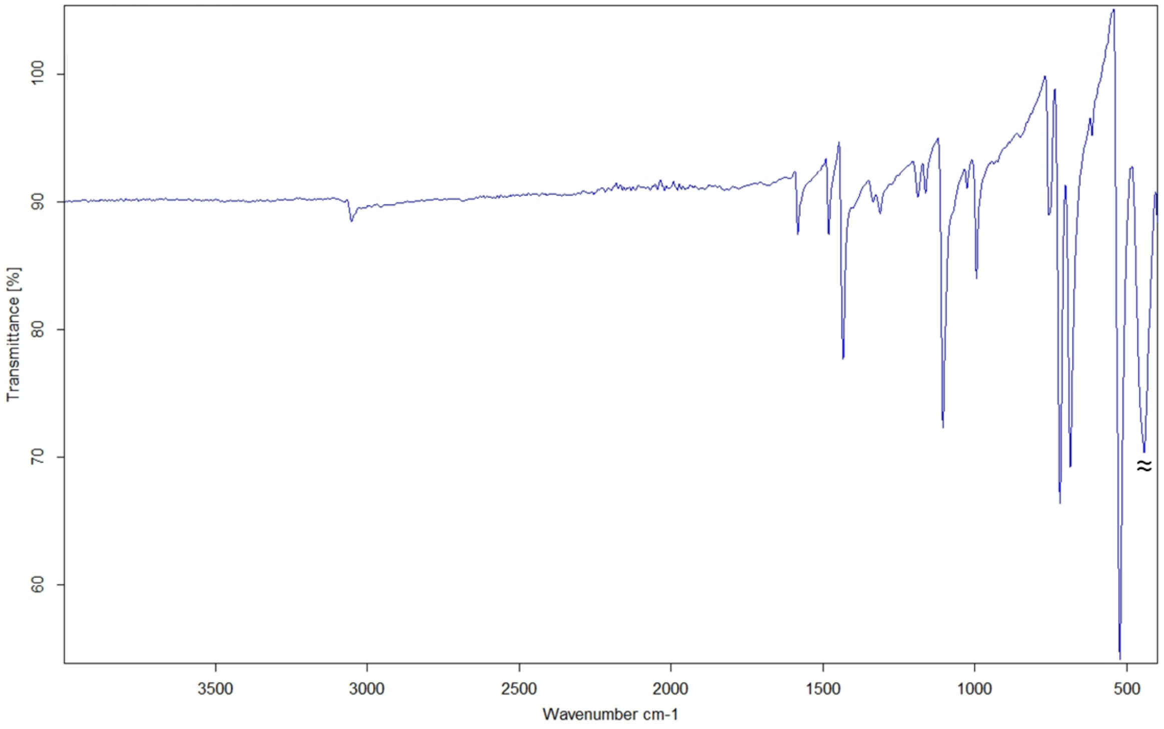

Figure S29: ATR-IR spectrum of crystalline $[PPh_4]_2[\mathbf{4}]$. ≈ denotes the vibrational band that is likely attributable to the [**4**] dianion (442 cm^{-1}) but that overlaps with a $[PPh_4]^+$ band.

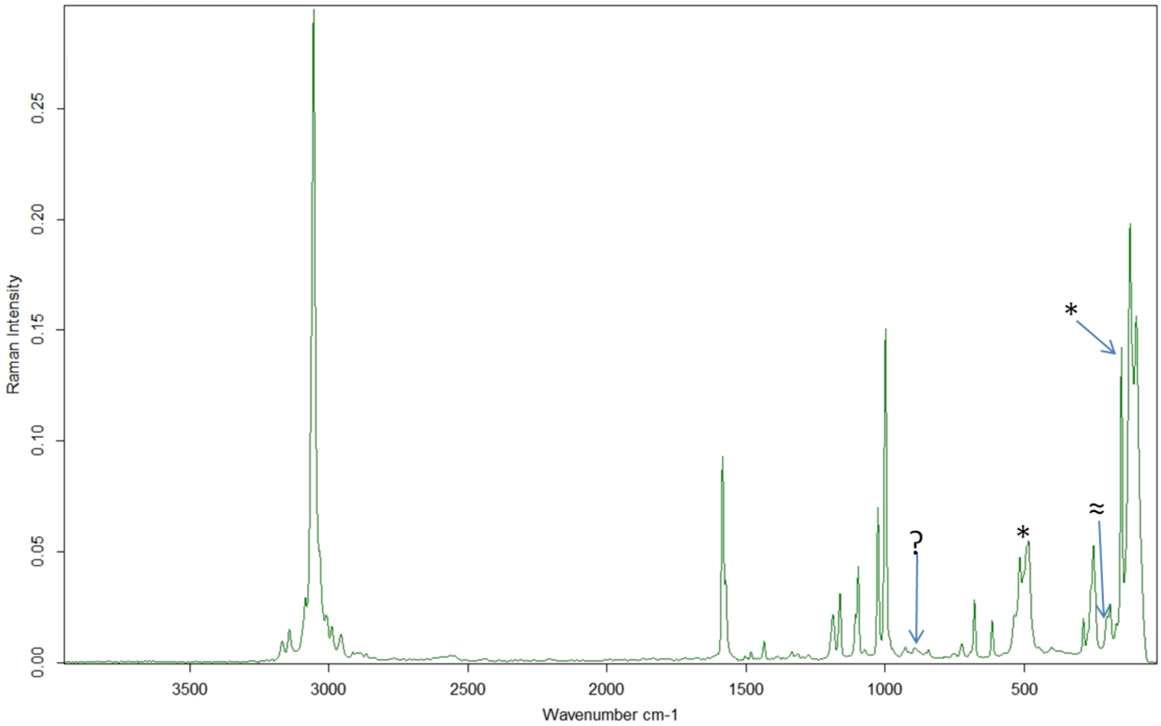

Figure S30: Raman spectrum of crystalline $[PPh_4]_2[\mathbf{4}]$. * denotes vibrational bands (complex feature at 500 cm^{-1} -with peaks at 534, 517, 486- and 152 cm^{-1}) that can unambiguously be attributed to the [**3**] dianion. ≈ denotes a band (200 cm^{-1}) that is likely attributable to [**3**] but could be due to the $[PPh_4]^+$ cation. ? denotes a band (892 cm^{-1}) that fits well with predicted values for the B-B stretching mode, but whose intensity matches poorly with the expected one. The complexity of the feature centered at 500 cm^{-1} is likely explained by the variety of possible phases for $[PPh_4]_2[\mathbf{4}]$, as suggested by MAS-NMR spectroscopy and as evidenced by single-crystal X-ray crystallography (*vide infra*). It could also be in part explained by the presence of trace amounts of the putative $[B_2I_5]^-$ species.

Solution behavior

Because $[PPh_4]_2[B_2X_6]$ (X = Cl, Br and I) are either completely insoluble or equilibrium mixtures in solution, their solution behavior is discussed separately here. Samples of B_2X_4 usually contain small amounts of BX_3, which typically increase slightly upon handling and reaction, even at low temperature. Upon treatment with X^- salts, BX_3 forms $[BX_4]^-$, which give very sharp signals in ^{11}B NMR spectroscopy. In solution NMR experiments, the careful control and evacuation of the heat of reaction is difficult and, consequently, the ^{11}B NMR spectra of samples containing $[B_2X_6]^{2-}$ or equilibrium mixtures thereof all show signs of decomposition. Even in conditions were two ^{11}B NMR signals would be expected from symmetry considerations, *e.g.* where $[B_2X_5]^-$ species are expected, no instance of pairs of signals of equal intensity were observed, which either suggest a symmetrical $[B_2X_5]^-$ species where the fifth X^- ligand bridges the two boron atoms, or a dynamic averaging of the two boron environments in solution (or even an equilibrium mixture between B_2X_4 and $[B_2X_6]^{2-}$). The thorough study of these monoanions is complicated by the much lower solubility of the

salts of the dianions (which therefore tend to crystallize first) and by the apparent lower stability of solutions containing "$[B_2X_5]^{-}$" or mixtures of $[B_2X_6]^{2-}$ and B_2X_4.[9] Because of these complications, such thorough studies are beyond the scope of this work and discussions of $[B_2X_5]^{-}$ remain tentative.

$[B_2Cl_6]^{2-}$ (2)

In contrast to the [PPh_4] salt, the [PPN] salt of **2** is soluble. Based on predicted small positive Gibbs free energies of reaction for $[B_2Cl_5]^{-} + Cl^{-} \rightarrow [B_2Cl_6{}^{2}]^{-}$, we suspected the possibility of an equilbrium mixture, so we examined the reaction of B_2Cl_4 with [PPN]Cl by ^{11}B NMR spectroscopy. Solutions were kept at *ca.* –20 °C between NMR acquisitions. The reaction of B_2Cl_4 with two equivalents of [PPN]Cl was apparently somewhat exothermic and led to the formation of detectable amounts of the $[BX_4]^{-}$ decomposition product observed as a very sharp singlet at 7.6 ppm. No other significant resonance could be observed, and the spectrum did not change upon adding three more equivalents of [PPN]Cl. B_2Cl_4 was added to the mixture so as to have between one and two equivalents of [PPN]Cl for one equivalent of B_2Cl_4. A major new resonance at +36 ppm was observed, as well as a smaller one at +11.7 ppm. These resonances are a good match with the predicted values for $[B_2Cl_5]^{-}$ (assuming a rapid intramolecular exchange of a chloride ligand from one boron atom to the other, giving a predicted average chemical shift of *ca.* 33 ppm for the two inequivalent boron environments) and [**2**] (predicted 8.0 ppm), respectively (Table S3). Adding [PPN]Cl to this last mixture so as to obtain a 1: <2 ratio of B_2Cl_4 to [PPN]Cl leads to the consumption of most of the species with a chemical shift of 36 ppm and to the increase in intensity of the 11 ppm signal. While the identity of the species observed for stoichoimetries between 0 and 1 equivalent of [PPN]Cl remains uncertain, the disappearance of the major signal for stoichiometries close or above two equivalents of [PPN]Cl and the reappearance of a major signals at 11.7 ppm for stoichiometries between one and two equivalents of [PPN]Cl and its fit with predicted values for [**2**] and with the MAS-NMR chemical shift observed for isolated [PPh_4][**2**]$\cdot 2CH_2Cl_2$ strongly suggest the existence of **2** in solution and the rapid exchange of chloride ligands with stoichiometries above two equivalents of [PPN]Cl, leading to the collapse of the ^{11}B NMR signals. As a final confirmation, the solid-state structure of $[PPN]_2$[**2**]$\cdot 2CH_2Cl_2$ was derived by single-crystal X-ray diffraction from crystals obtained from the solution that produced the major ^{11}B NMR signal at 11.7 ppm. The absence of a signal belonging to **2** and the presence of a sharp signal belonging to $[BCl_4]^{-}$ in solutions where **2** is expected has been noted previously[9] and (incorrectly) suggested the non-existence of **2**.

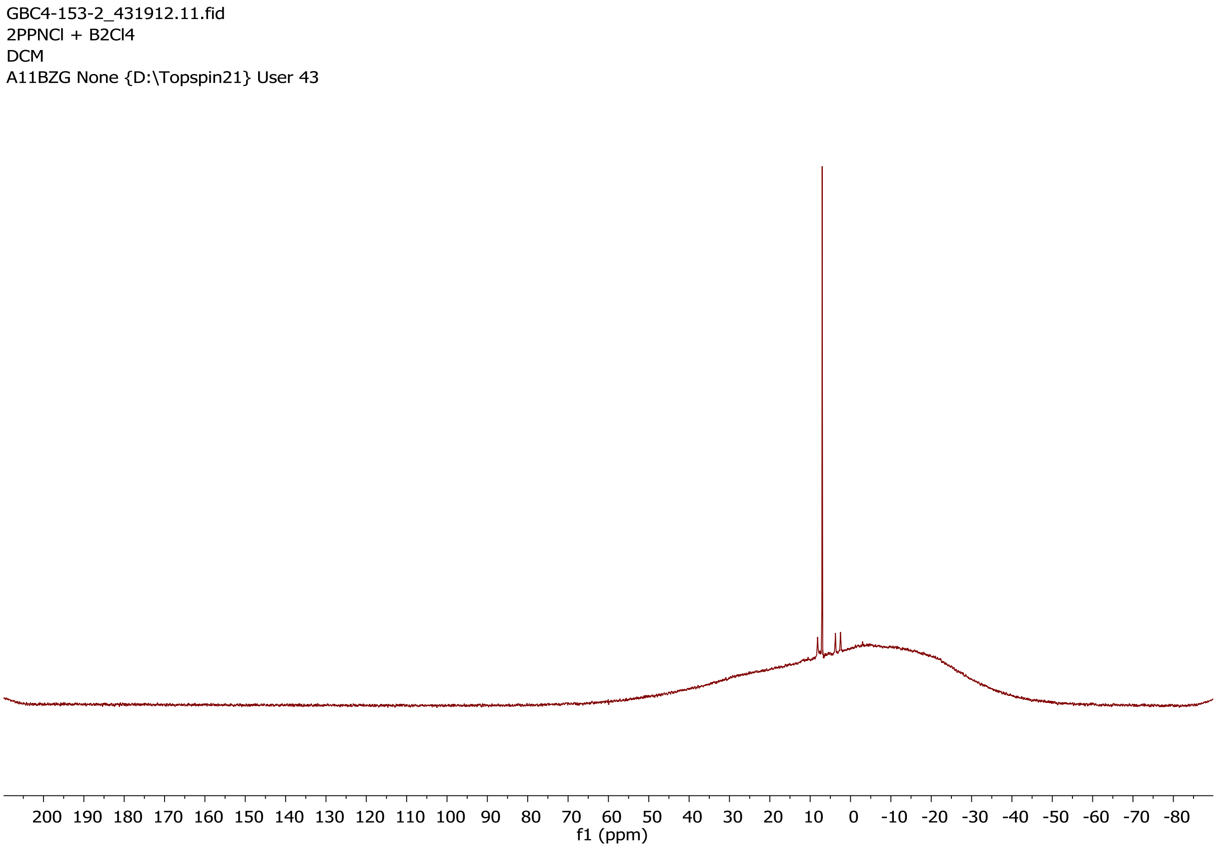

Figure S31: ^{11}B NMR spectrum of B_2Cl_4 + 2 equiv [PPN]Cl in CH_2Cl_2 (unlocked). The major signal at *ca.* 7 ppm belongs to $[BCl_4]^-$ and the other small signals strongly correlate to small amounts of other decomposition products. Note that that the combined signal integration is much lower than that expected from the amount of B_2Cl_4 in the system.

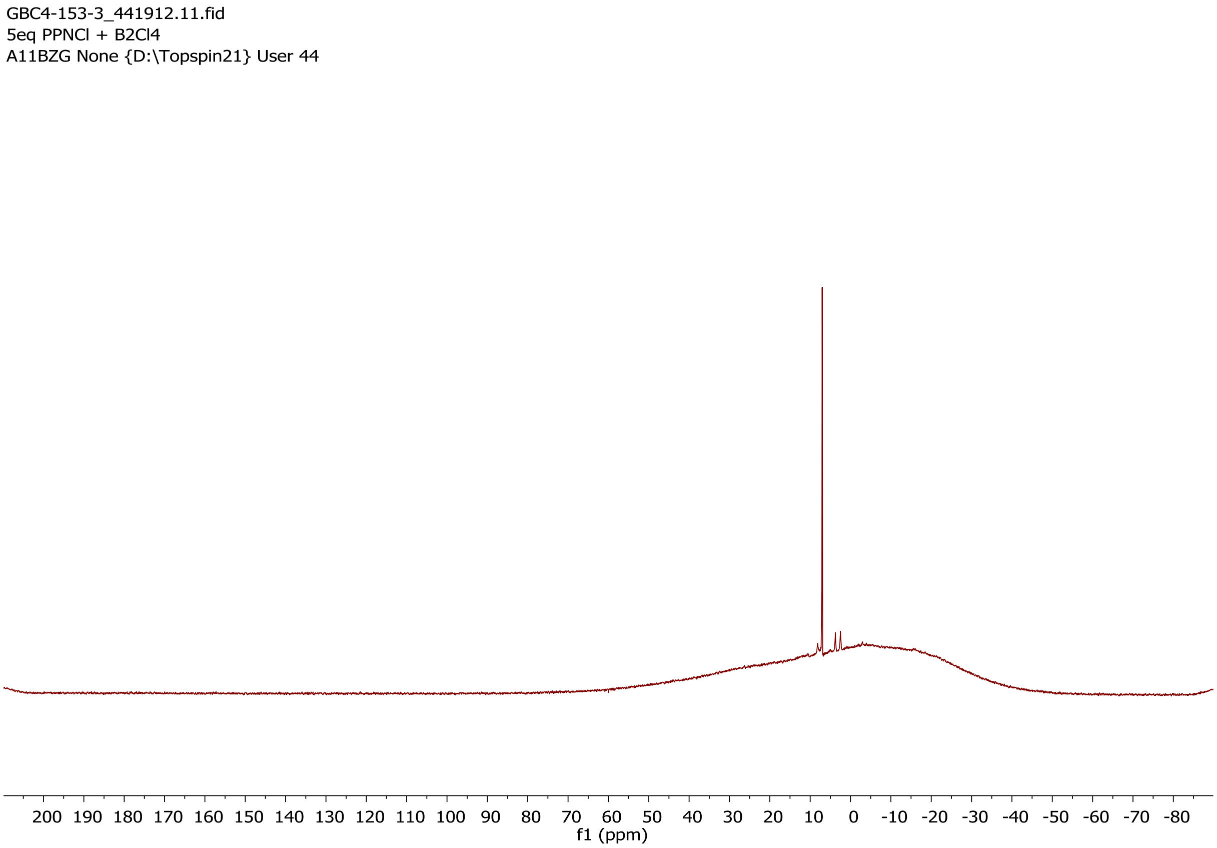

Figure S32: ^{11}B NMR spectrum of B_2Cl_4 + 5 equiv [PPN]Cl in CH_2Cl_2 (unlocked). Note no substantial change from the previous spectrum.

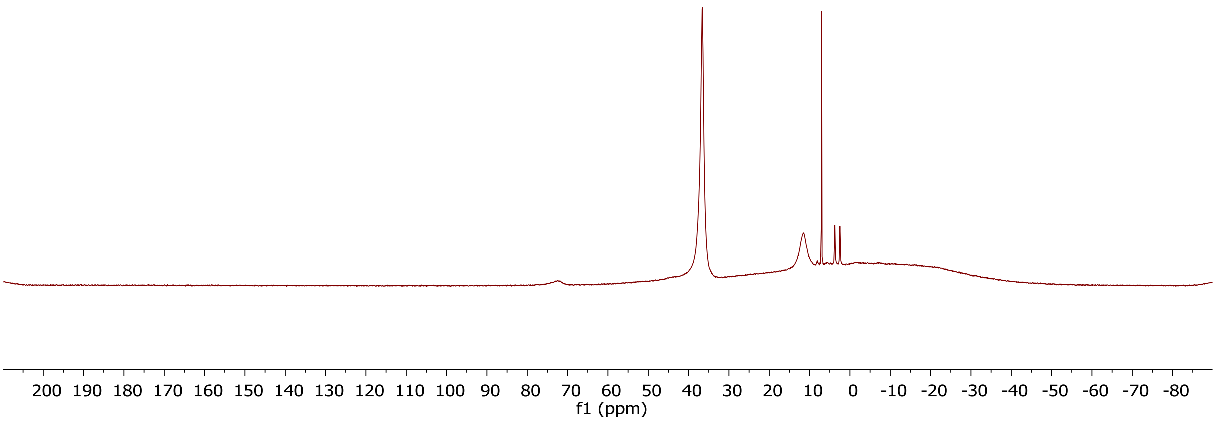

Figure S33: ^{11}B NMR spectrum of B_2Cl_4 added to sample of Figure S32 so as to obtain a *ca.* 1:1.5 ratio of B_2Cl_4 : [PPN]Cl in CH_2Cl_2 (unlocked). Note the large increase in total signal intensity from the previous spectrum. The signal at *ca.* 36 ppm is tentatively attributed to the putative $[B_2Cl_5]^-$ and the signal at *ca.* 11 ppm to $[B_2Cl_6]^{2-}$.

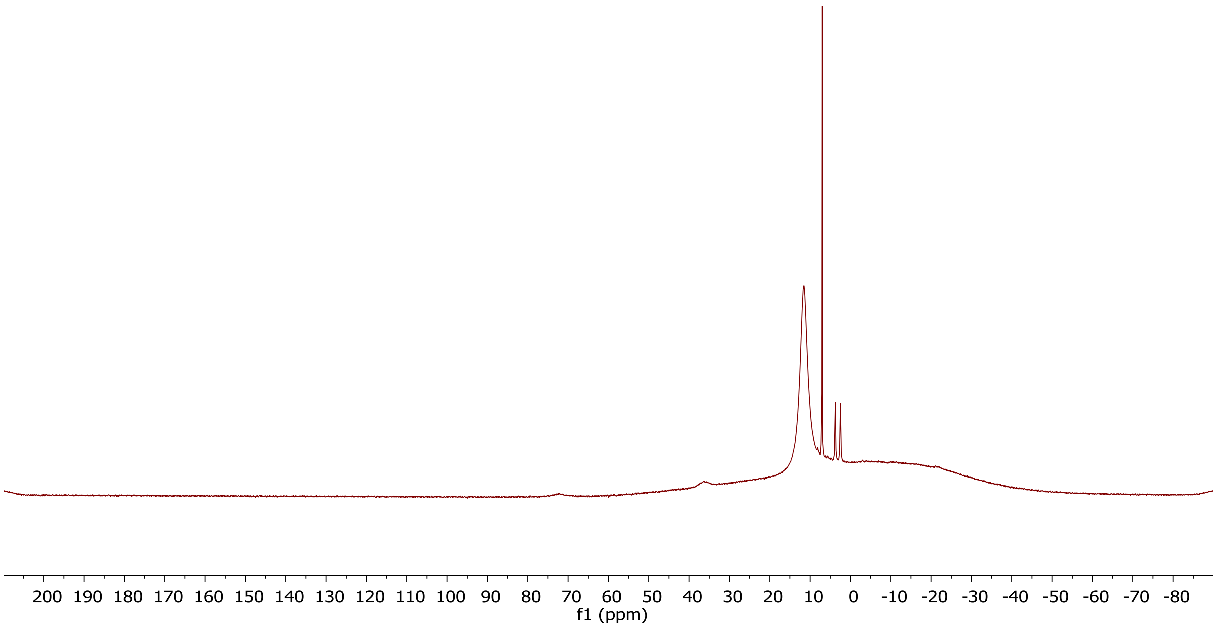

Figure S34: ^{11}B NMR spectrum of [PPN]Cl added to the sample of Figure S33 so as to obtain a *ca.* 1:<2 ratio of B_2Cl_4 to [PPN]Cl in CH_2Cl_2 (unlocked). Note the nearly complete consumption of the species with a signal at 36 ppm and its apparent conversion into the species with a chemical shift at 11 ppm (**2**).

$[B_2Br_6]^{2-}$ (3)

B_2Br_4 was treated with 2, 4 and 10 equivalents of [TBA]Br. A ^{11}B NMR resonance at *ca.* 1.5 ppm was observed with 2 equiv, which broadens significantly when 4 and 10 equivalents of [TBA]Br are used. Similarly to **2**, it appears that increasing the concentration of bromide ions in solution leads to a rapid exchange of the ligands. The extent of broadening, contrary to **2**, does not lead to the collapse of the ^{11}B NMR signal and could therefore potentially be due to the change in viscosity of the solution. At any rate, the center of the resonance does not move significantly and its chemical shift is in good agreement with solid-state MAS ^{11}B NMR spectra acquired on isolated $[PPh_4]_2[\mathbf{3}]\cdot xCH_2Cl_2$ (–4 ppm), indicating that **3** exists in solution.

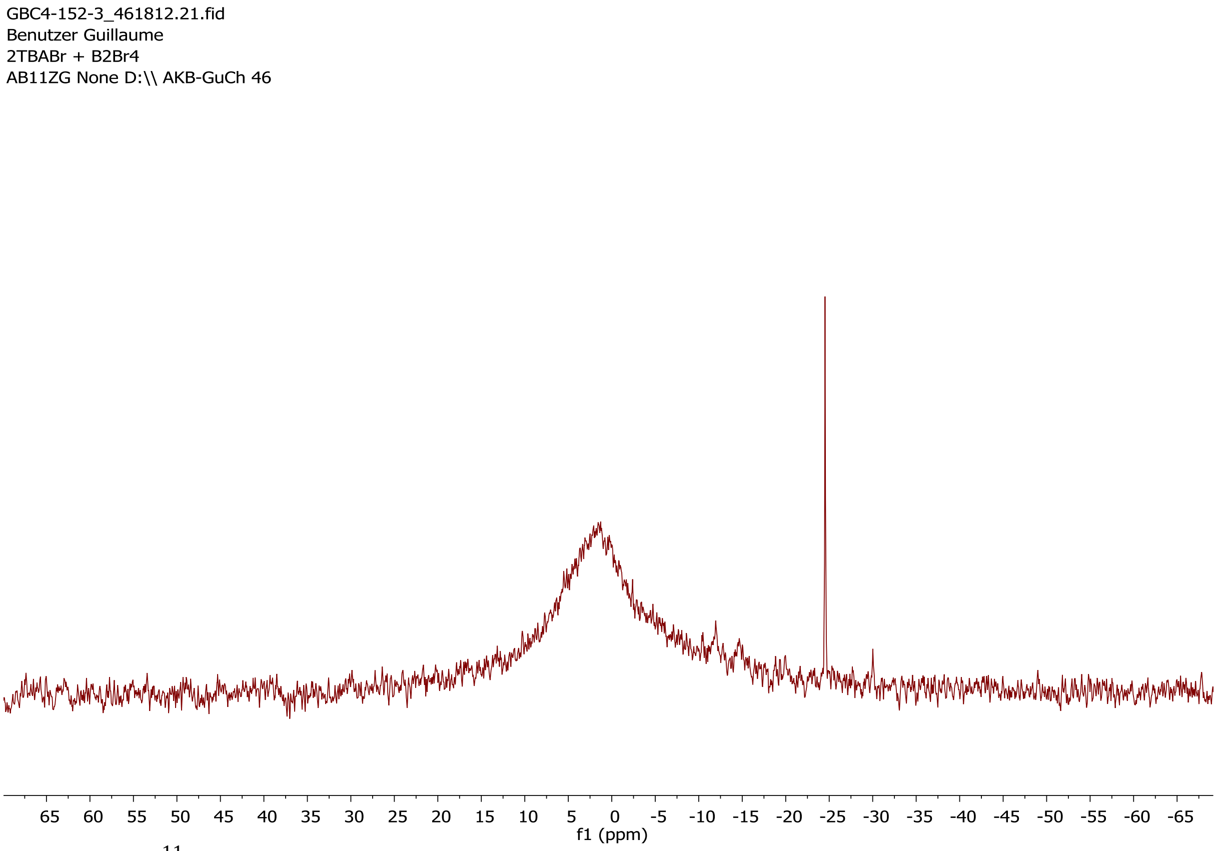

Figure S35: ^{11}B NMR spectrum of B_2Br_4 + 2 equiv of [TBA]Br in CH_2Cl_2 solution. The signal at *ca.* –25 ppm belongs to $[BBr_4]^-$.

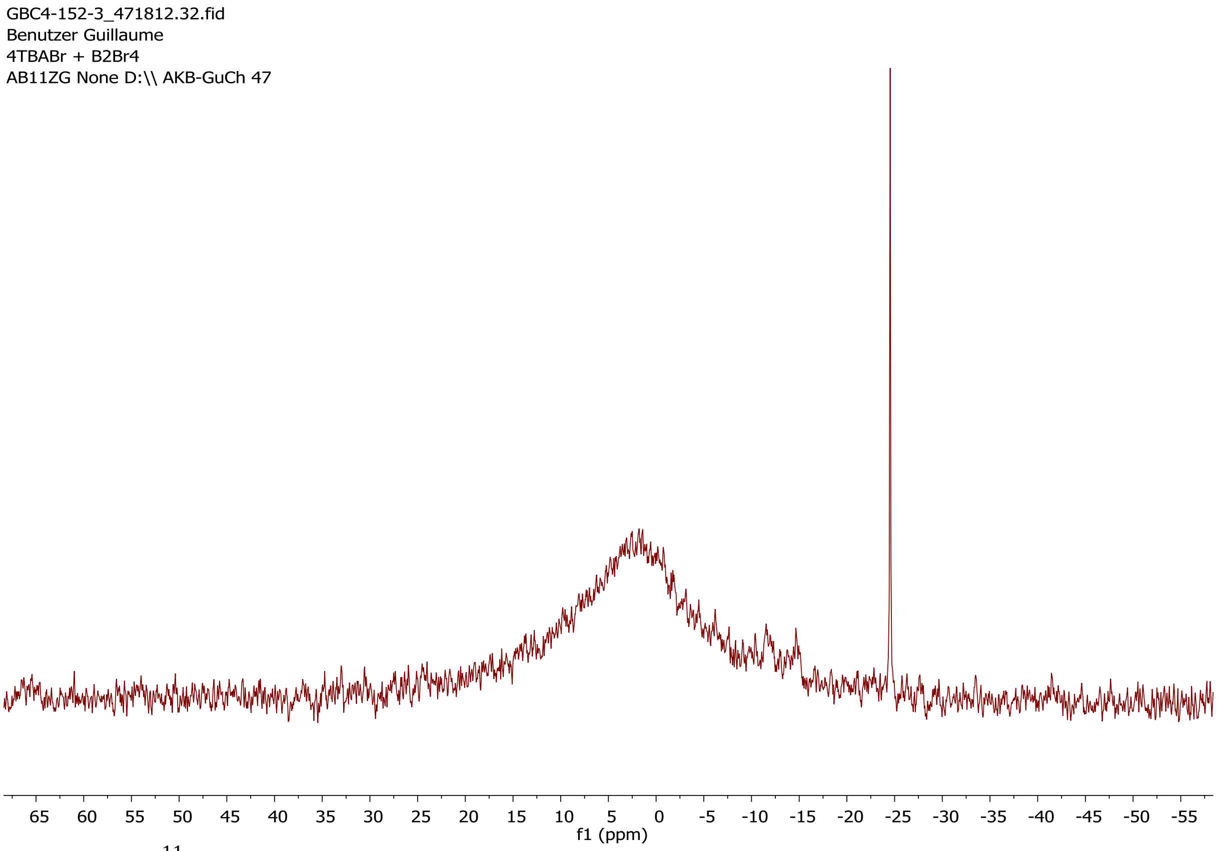

Figure S36: ^{11}B NMR spectrum of B_2Br_4 + 4 equiv of [TBA]Br in CH_2Cl_2 solution. The signal at *ca* –25 ppm belongs to $[BBr_4]^-$.

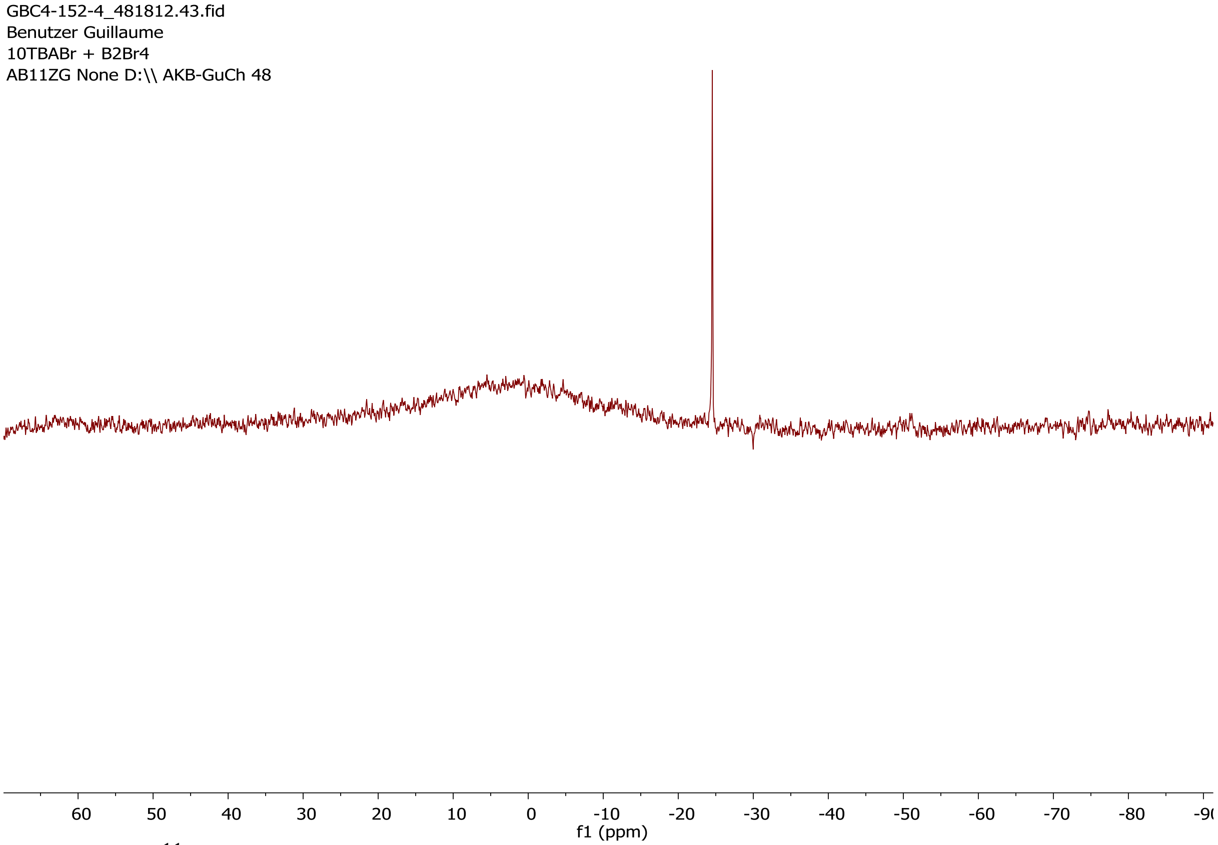

Figure S37: ^{11}B NMR spectrum of B_2Br_4 + 10 equiv of [TBA]Br in CH_2Cl_2 solution. The signal at *ca.* –25 ppm belongs to $[BBr_4]^-$.

$[B_2I_6]^{2-}$ (4)

Based on extrapolations on trends observed for X= F, Cl and Br and on the predicted formation enthalpies of $[B_2X_6]^{2-}$, **4** was expected to be relatively labile. This was confirmed by the observation of equilibria in the [TBA]I/B_2I_4 system, which manifested itself as a main ^{11}B NMR signal progressively converging to a value of *ca.* –29.9 ppm upon treatment with 4 to 10 equivalents of [TBA]I. A ^{11}B NMR signal at *ca.* 9 ppm could be observed for the 1:1 stoichoimetry. At a 2:1 stoichiometry, a weak and broad signal at *ca.* 8 ppm was observed. At a 4:1 ratio, a significantly more intense signal was observed at –28 ppm and a much broader one at –16 ppm. At 6:1 and 10:1 stoichiometries, the most intense signal shifts towards –30 ppm while two other much broader signals shift from *ca.* –1 and –16 to –12 and –21 ppm, respectively. The relative instability of B_2I_x species in CH_2Cl_2 solution makes accurate assignments difficult, but the observations suggest the formation of equilibria favoring **4** at high I^- concentration. Solid-state NMR-MAS spectra indicate a chemical shift of –35 ppm for the major species in bulk isolated $[PPh_4]_2[\mathbf{4}]$, in good agreement with our observations in solution.

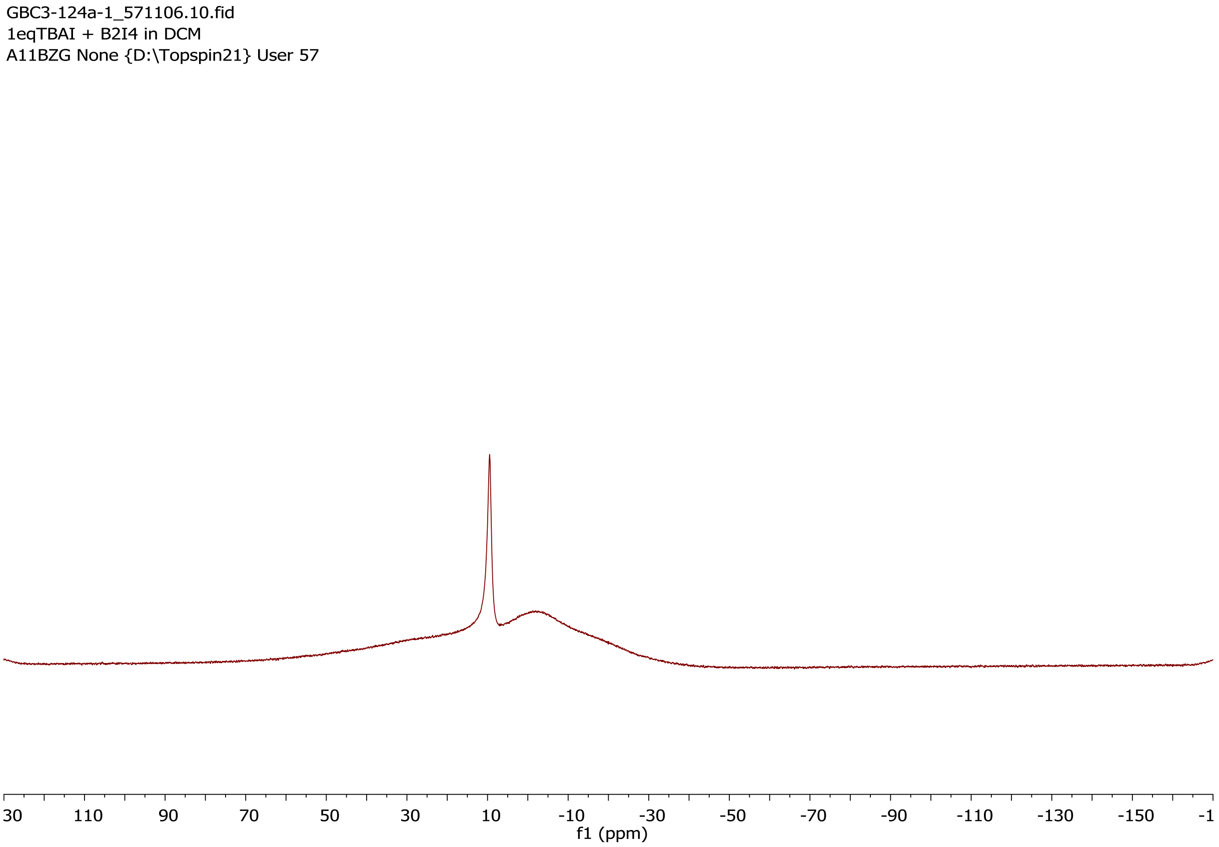

Figure S38: ^{11}B NMR spectrum of B_2I_4 + 1 equiv [TBA]I in CH_2Cl_2 (unlocked). The signal at *ca.* 10 ppm is tentatively assigned to $[B_2I_5]^-$.

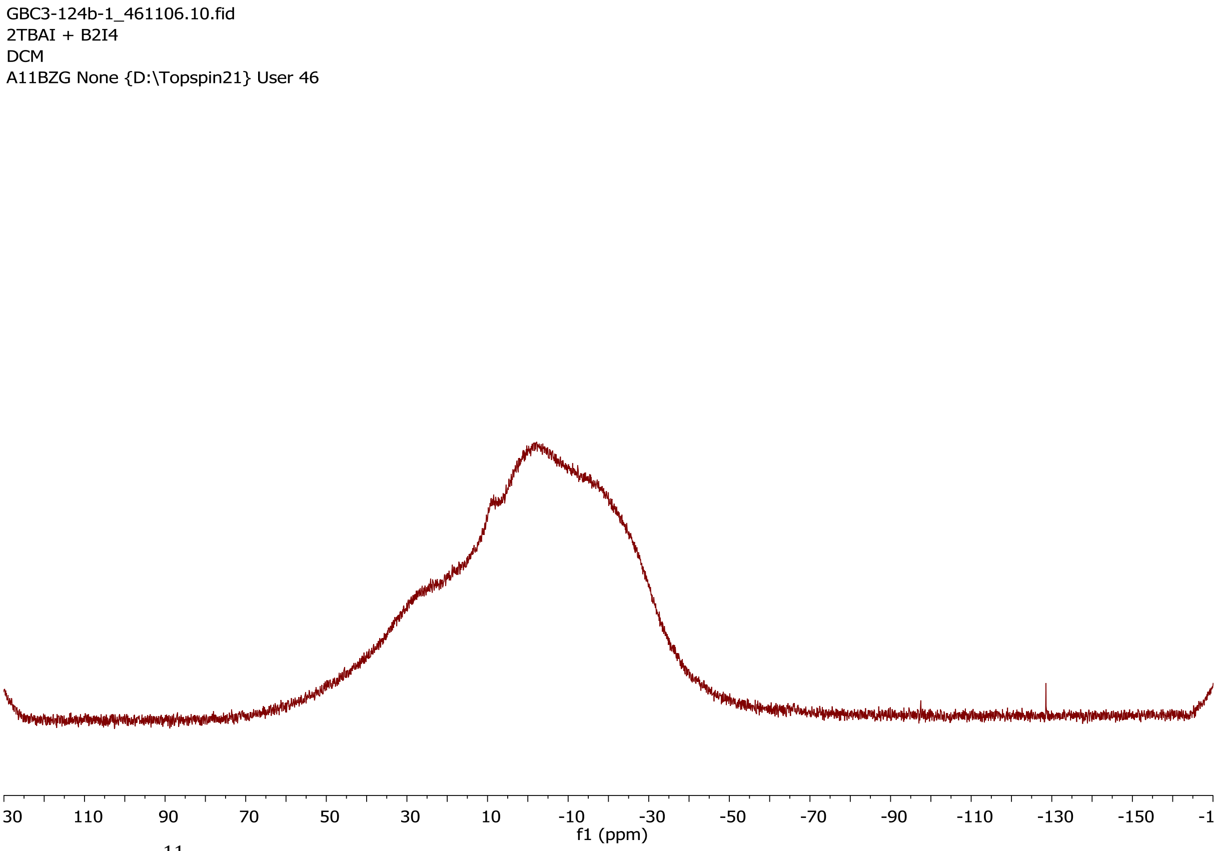

Figure S39: ^{11}B NMR spectrum of a separate experiment in dichloromethane (unlocked). B_2I_4 + 2 equiv [TBA]I. Only a weak and broad signal can be observed at *ca.* 8 ppm. The sharp signal at *ca.* –130 ppm is consistent with the presence of traces of BI_4^-.

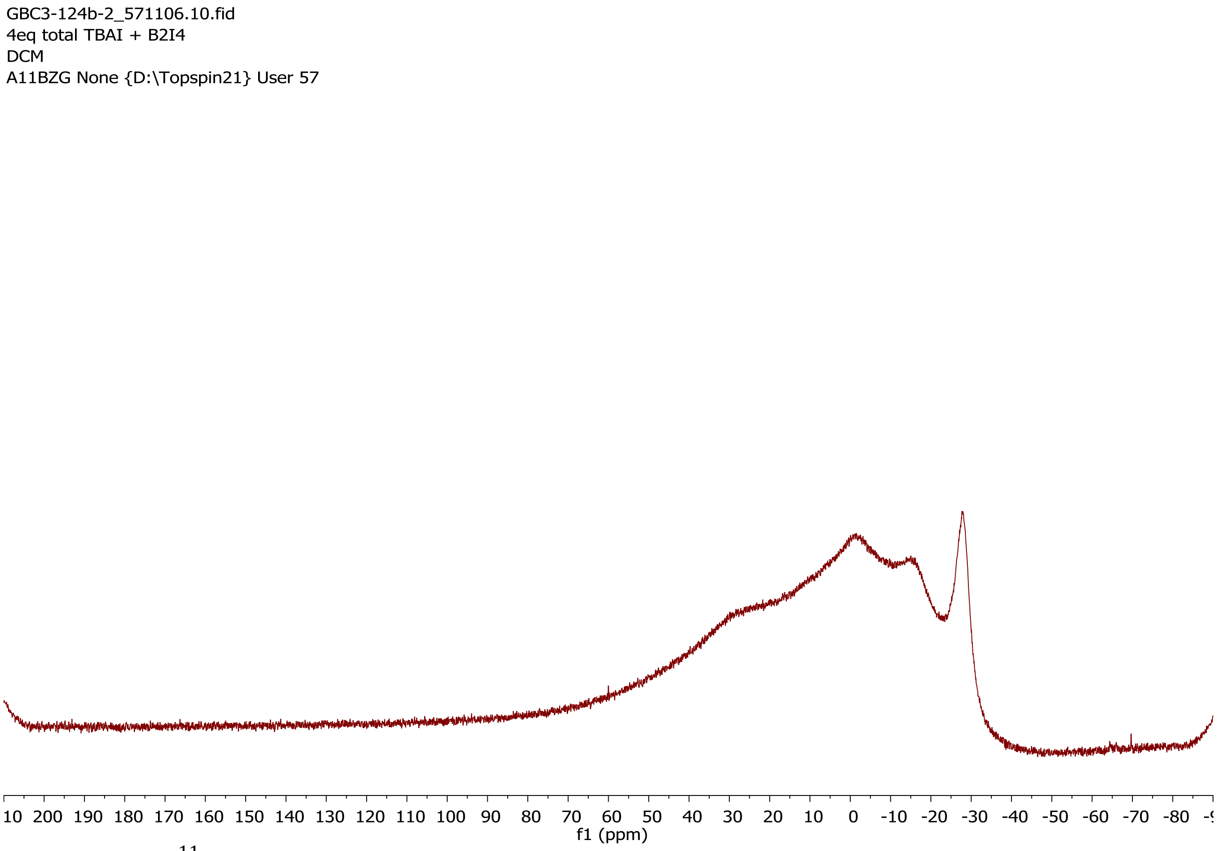

Figure S40: ^{11}B NMR spectrum in dichloromethane (unlocked). Addition of 2 equiv of [TBA]I to the mixture of Figure S39. The signal at *ca.* 0 ppm is difficult to distinguish from the background signal, while the signals at *ca* –10 and –28 ppm clearly belong to new species.

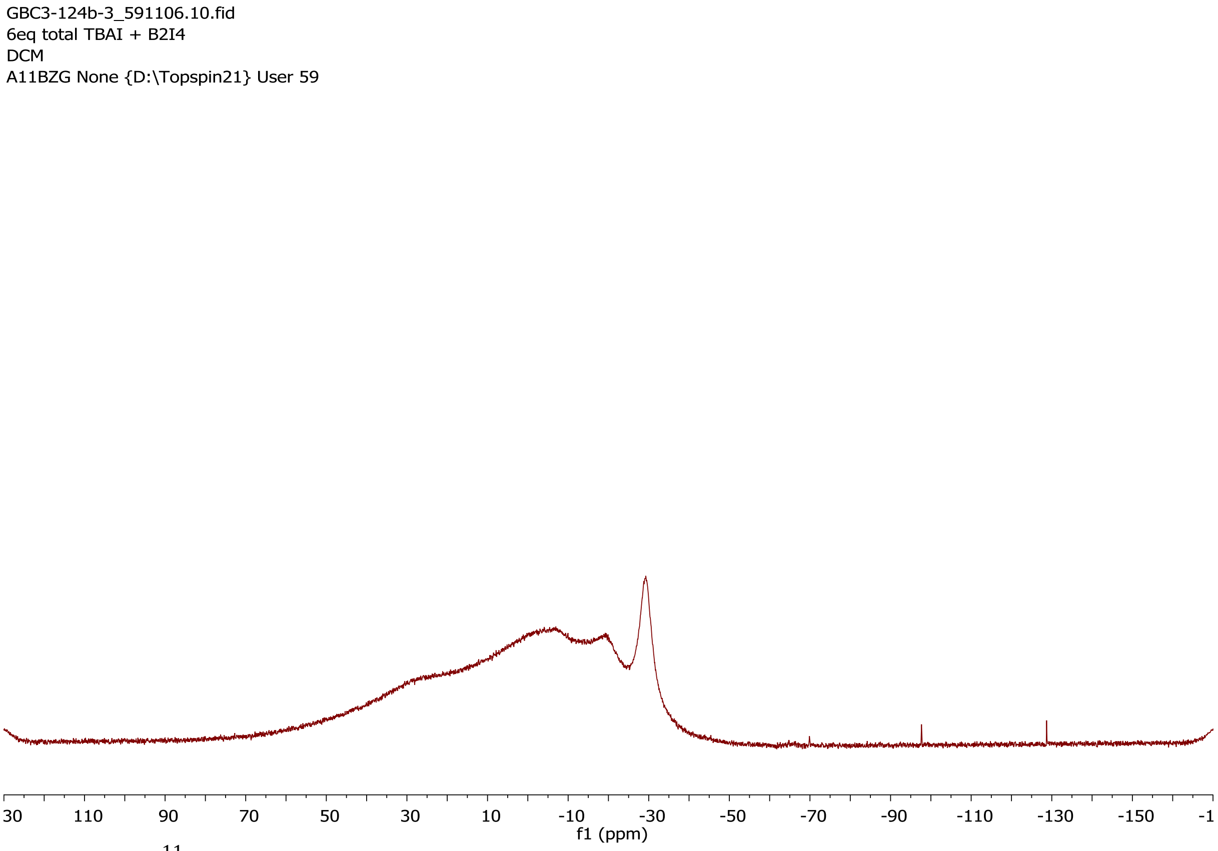

Figure S41: ^{11}B NMR spectrum in dichloromethane (unlocked). Addition of 2 equiv of [TBA]I to the mixture of Figure S40. The two major signals have shifted to –20 and –30 ppm,

respectively. The signal at *ca.* –100 ppm belongs to an unidentified species, likely highly symmetrical.

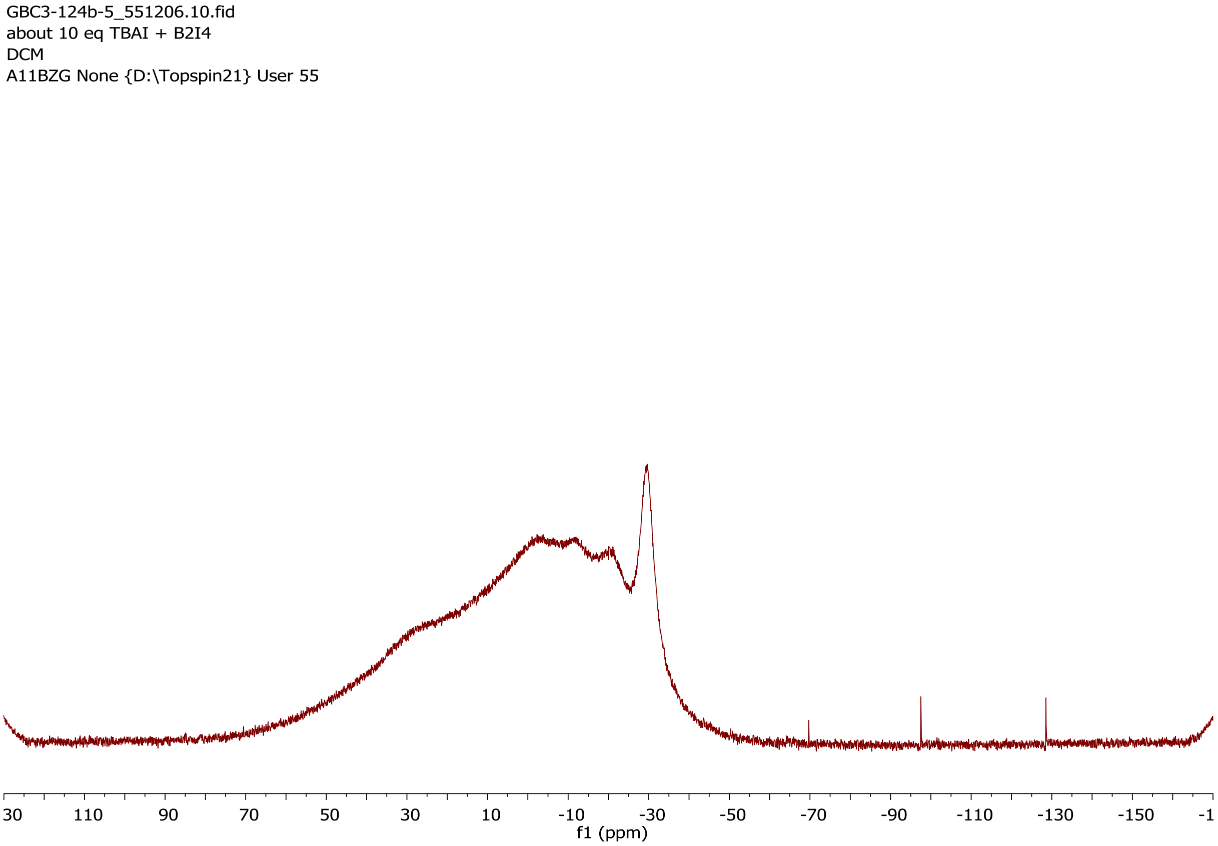

Figure S42: Addition of 4 equiv of [TBA]I to the mixture of Figure S41. The major signal at *ca.* –30 ppm belongs to **4**, while the signals at *ca.* –10 and –15 ppm belong to unidentified species, either belonging to B_xI_x decomposition products, to intermediate chemical shift values derived from $[B_2I_4]/[B_2I_5]^{-}/[B_2I_6]^{2-}$ equilibria, or to a combination thereof. The signals at *ca.* –70 and –100 ppm belongs to unidentified species, likely highly symmetrical.

Further Notes on Stability

In analogy to B_2I_4 and other boron iodides, **4** was assumed to be photosensitive and was therefore protected from light whenever possible. In contrast to **2**, the content of cocrystallized solvent in salts of **3** and **4** can be highly variable, as attested by the multiple phases of **3** and **4** suggested by solid-state NMR data as well as the two different crystal structures determined for **4.** Moreover, ^{1}H NMR spectra and elemental analysis of $[PPh_4]$[**3**]$\cdot 2CH_2Cl_2$ showed that one molecule of dichloromethane could easily diffuse out of the microcrystalline material under vacuum.

Notes on Behavior in Coordinating Solvents

The stability of **1** is predicted to be dramatically greater than that of **2-4** with respect to the loss of a halide ligand, in good agreement with our experimental observations. Indeed, **1** is unambiguously observed in solution and the fluoride anions cannot be displaced by a donor ligand (according to [Eq. (S1 and S2)]), even when the compound is dissolved in acetonitrile, for example (the fluoride-acetonitrile displacement reaction free energy is predicted to be endergonic by a prohibitive 22.3 kcal/mol, see Computational Data). For **2**, and by

extrapolation **3** and **4**, at least one of these displacement reactions is predicted to be exergonic, in agreement with the dissolution of the $[PPh_4]$ salts of **2** and **3** in acetonitrile and formation of new species, as observed by ^{11}B NMR spectroscopy (see Computational Data).

$$[B_2X_6]^{2-} + \text{solvent} \underset{+\,X^-}{\overset{-\,X^-}{\rightleftharpoons}} [X_3BBX_2(\text{solvent})]^- \qquad \text{(S1)}$$

$$[X_3BBX_2(\text{solvent})]^- + \text{solvent} \underset{+\,X^-}{\overset{-\,X^-}{\rightleftharpoons}} [(\text{solvent})X_2BBX_2(\text{solvent})] \qquad \text{(S2)}$$

Notes on Bromine/Chlorine exchange between $[PPh_4]Cl$ and $B_2Br_4{\cdot}2SMe_2$

By simple displacement reactions, we were able to demonstrate qualitatively that **2** is indeed significantly thermodynamically more favored than **3**. For example, while adding two equiv. of $[PPh_4]Br$ to $B_2Br_4{\cdot}2SMe_2$ only results in an equilibrium mixture, showing that Br^- cannot effectively compete with SMe_2 for binding to boron, the addition of six equiv. of $[PPh_4]Cl$ to $B_2Br_4{\cdot}2SMe_2$ results in a clean substitution with the crystallization of $[PPh_4]_2$[**2**]$\cdot 2CH_2Cl_2$. Because $[PPh_4]_2$[**3**]$\cdot 2CH_2Cl_2$ would also be expected to precipitate from a CH_2Cl_2 solution, this qualitatively supports the higher stability of **2** with respect to **3**.

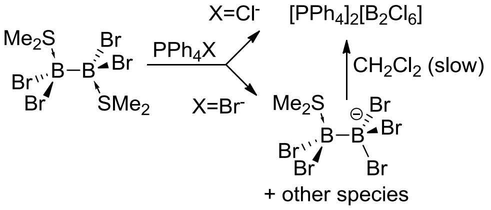

Scheme S1: Qualitative demonstration of the thermodynamically favorable formation of **2** with respect to **3**.

Notes on Bromine/Chlorine exchange between $[PPh_4]Br/B_2Br_4{\cdot}2SMe_2$ and CH_2Cl_2

The reaction of $B_2Br_4{\cdot}2SMe_2$ with $[PPh_4]Br$ in dichloromethane yields a solution that contains at least two species as determined by ^{11}B NMR spectroscopy. Surprisingly, the $B_2Br_4{\cdot}2SMe_2/[PPh_4]Br$ system slowly reacts with the dichloromethane solvent and eventually yields significant amounts of $[PPh_4]_2$[**2**]$\cdot 2CH_2Cl_2$, which crystallizes out of the mixture after weeks at room temperature (Scheme S1). Similar exchanges have been observed between B_2Br_4 and CCl_4.[21]

Notes on Vibrational Data

The accurate assignment of vibrational bands was complicated by the intense bands of the organic cations. The expected B-B stretching bands was not unambiguously observed for any of the $[B_2X_6]^{2-}$ species by Raman spectroscopy but are expected to be either relatively weak and/or to be hidden by the cation bands. Otherwise, the vibrational data is in good agreement with our theoretical predictions and tentative assignments can be found in Table S4 to Table S7. The most conclusive observations concern the IR-active out-of-phase symmetric and asymmetric B-X stretches, especially the very intense bands for **1**. The vibrational spectra are in poor agreement with published data for several larger borane subhalides[10] or for $[BX_4]^-$.[8, 11]

Computational Data

Thermochemical estimates for the halide and solvent coordination reactions were calculated with the Gaussian 09[12] suite. Structures were optimized using the Wb97XD[13] functional with 6-311+g(d,p) basis set (except for iodine-containing molecules, for which 6-311+g(d,p) was used for all light elements and lanl2dz[14] was used for iodine) with implicit solvation effects included with the SMD[15] method (solvent = dichloromethane). Frequency calculations were performed to confirm that the structures were minima on the potential energy surface. The same reactions were probed using the B3lyp[13b, 16] functional in the same conditions, which yielded values in somewhat poorer agreement with our data and with other systems and are tabulated for comparison purposes. Gibbs free energies are corrected for 1M state. Reactions listed in Table S2 that involve the substitution of halide ligands by acetonitrile are expected to be more favorable by *ca.* 1.7 kcal/mol if performed in neat acetonitrile (correction for 19 M for acetonitrile). The geometry of B_2F_4 has been and apparently continues to be a somewhat contentious issue. Many sources conclude that the equilibrium geometry should be planar (D_{2h})[17] but some reports suggest a staggered geometry (D_{2d}),[18] although in all cases the barrier for the rotation about the B-B bond is expected to be very low.[19] Our optimizations using implicit solvation modeling yielded a slightly distorted D_{2h} structure as the minimum and, for the sake of consistency, all comparisons and calculations used this geometry for B_2F_4.

^{11}B NMR shifts were computed with the GIAO method and referenced to $[BF_4]^-$ (exp. value at –1.4 ppm; cf. published values at –1.6 ppm for $[Ph_3C][BF_4]$[20]). Already with chlorine-containing molecules, shifts deviated significantly from expected values for test species. A calibration curve was therefore used for all chlorine-containing molecules (Figure S43 and Figure S44. Values for molecules bearing heavier halogens (Br, I) were completely inconsistent with known values for test species, even when large basis sets were used, and attempts to predict values for these systems were discontinued. Predicted vibrational frequencies are reported unscaled. Predicted values are in satisfactory agreement with published values for test species ($[BX_4]^-$) (Table S1).

Table S1: Comparison of our predicted frequencies for characteristic vibrational modes on test species $[BX_4]^-$ with published experimental values in cm^{-1}.

Molecule	Mode	B3lyp	Experimental	ωb97xd
BF_4^-	B-X symmetric stretch (ν_1)	733.8	780[21]	741.5
	B-X asym. stretch (ν_3)	990.3	*ca* 1100[21]	1018.3
BCl_4^-	B-X symmetric stretch (ν_1)	394.1	405[8]	407.9
	B-X asym. stretch (ν_3)	643.4	670[8]	693.2
BBr_4^-	B-X symmetric stretch (ν_1)	231.8	243[8]	239.6
	B-X asym. stretch (ν_3)	550.3	605[8]	597.2
BI_4^-	B-X symmetric stretch (ν_1)	160.3	-	177.0
	B-X asym. stretch (ν_3)	451.3	517[11b]	564.6

Table S2: Gibbs free energy and (enthalpy) changes for key complex formation reactions, in kcal/mol.

Reaction	B3lyp	Wb97xd
$B_2F_4 + F^- \rightarrow [B_2F_5]^-$	–37.1 (–42.5)	–39.3 (–44.7)
$[B_2F_5]^- \rightarrow [B_2F_6]^{2-}$	–10.6 (–18.4)	–13.0 (–20.7)
$BF_3 + F^- \rightarrow [BF_4]^-$	–42.0 (–50.0)	–43.8 (–51.8)
$[B_2F_6]^{2-} + CH_3CN \rightarrow [B_2F_5(NCCH_3)]^- + F^-$	22.5 (20.9)	22.3 (20.4)
$B_2Cl_4 + Cl^- \rightarrow [B_2Cl_5]^-$	–14.2 (–20.8)	–19.2 (–26.0)

$[B_2Cl_5]^- + Cl^- \rightarrow [B_2Cl_6]^{2-}$	5.6 (–1.2)	0.8 (–6.3)
$BCl_3 + Cl^- \rightarrow [BCl_4]^-$	–14.0 (–20.8)	–17.4 (–25.3)
$[B_2Cl_6]^{2-} + CH_3CN \rightarrow [B_2Cl_5(NCCH_3)]^- + Cl^-$	–3.0 (–6.0)	–2.8 (–5.5)
$[B_2Cl_5(NCCH_3)]^- + CH_3CN \rightarrow B_2Cl_4(CH_3CN)_2 + Cl^-$	5.1 (1.7)	5.1 (2.2)
$B_2Br_4 + Br^- \rightarrow [B_2Br_5]^-$	–12.1 (–19.7)	–18.6 (–25.2)
$[B_2Br_5]^- + Br^- \rightarrow [B_2Br_6]^{2-}$	11.9 (5.3)	6.9 (–0.4)
$BBr_3 + Br^- \rightarrow [BBr_4]^-$	–12.7 (–19.2)	–17.3 (–23.9)
$B_2I_4 + I^- \rightarrow [B_2I_5]^-$	0.7(–5.0)	–5.9 (–12.7)
$[B_2I_5]^- + I^- \rightarrow [B_2I_6]^{2-}$	12.2 (5.6)	4.1 (–2.8)
$BI_3 + I^- \rightarrow [BI_4]^-$	1.2 (–6.0)	–4.8 (–12.3)

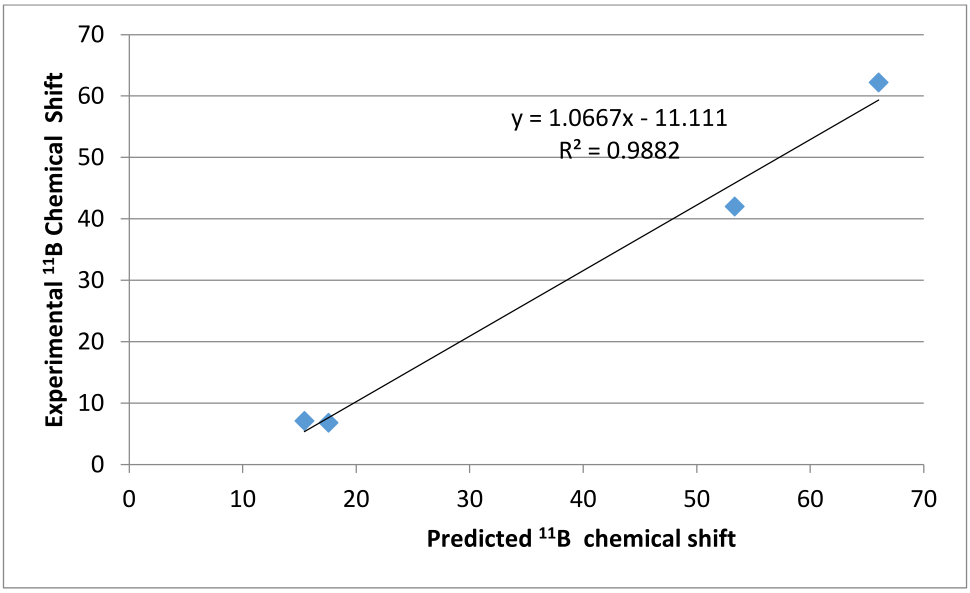

Figure S43: Calibration curve for ^{11}B chemical shifts for chlorine-containing species (B3lyp). From left to right: $BCl_3{\cdot}SMe_2$, $[BCl_4]^-$, BCl_3, B_2Cl_4.

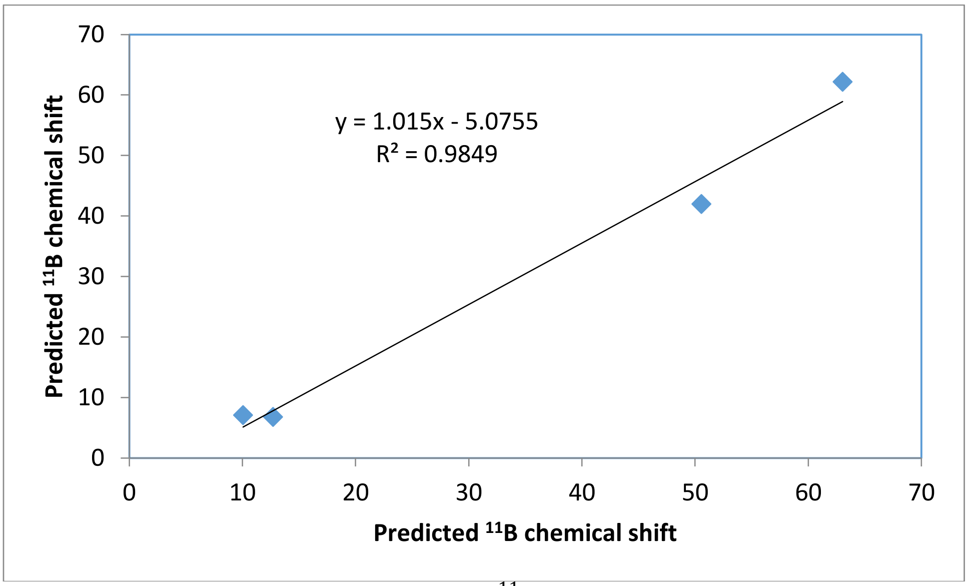

Figure S44: Calibration curve for ¹¹B chemical shifts for chlorine-containing species (ωb97xd). From left to right: $BCl_3{\cdot}SMe_2$, $[BCl_4]^-$, BCl_3, B_2Cl_4.

Table S3: Predicted chemical shifts in CH_2Cl_2.

	**B3lyp (fitted)**		**Experimental**		**ωb97XD (fitted)**	
	**B(1)**	**B(2)**	**B(1)**	**B(2)**	**B(1)**	**B(2)**
$[B_2F_6]^{2-}$ (**1**)	5.4		5.7		4.7	
$[F_3B(1)\text{-}B(2)F_2]^-$	0.1	31.5	-		–0.3	30.5
B_2F_4	23.6		23.6[6]		23.0	
BF_3	11.0		10 (cf. 9.4)[22]		11.3	
$[B_2Cl_6]^{2-}$ (**2**)	18.4 (8.5)		12		12.5 (8.0)	
$[Cl_3B(1)\text{-}B(2)Cl_2]^-$	10.2 (–0.3)	73.0 (66.8)	-		4.9 (0.3)	69.5 (65.5)
$[(CH_3CN)Cl_2B(1)\text{-}B(2)Cl_3]^-$	3.6 (–7.3)	16.5 (6.5)	6.2		1.1 (-3.5)	10.6 (5.7)
$(CH_3CN)Cl_2B\text{-}BCl_2(NCCH_3)$	2.4 (–8.6)		-		–0.1(–4.8)	
B_2Cl_4	66 (59.3)		62.2[6]		62.9 (58.9)	
BCl_3	53.4 (45.8)		46 (cf. 41.9)[20]		50.4 (46.2)	
BCl_4^-	17.6 (7.6)		7.0 (cf. 6.8 NH_4BCl_4)[20]		11.8 (7.3)	
$BCl_3{\cdot}SMe_2$	11.0 (5.4)		7.1[23]		10.2 (5.6)	

Table S4: Predicted vibrational frequencies (cm^{-1}) for **1**.

**Mode #**	**Tentative experimental assignment (r = Raman; i = IR)**	**Predicted frequency (IR int.) [Raman int.]**		**Approximate description of vibrational mode**
		**B3lyp**	**ωb97xd**	
**1**		61.9 (0) [0]	46.6 (0)[0]	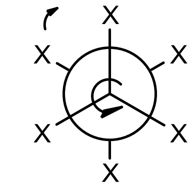 Torsion about B-B bond
**2**		138.0 (1.9) [0]	140.1 (2.2)[0]	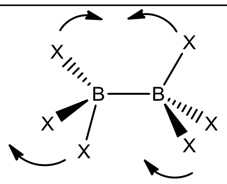 rock
**3**		159.7 (2.7) [0]	161.1 (3.0)[0]	
**4**		275.2 (0) [0.9]	276.9 (0)[1.2]	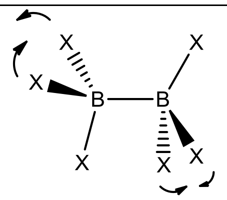 BX_2 bend
**5**		287.2 (0) [0.9]	289.5 (0)[1.0]	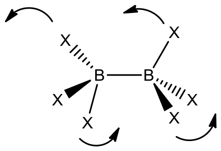 rock
**6**		290.0 (0) [0.9]	293.0 (0)[0.9]	

**7**		394.2<br>(0.9) [0]	394.5<br>(1.2)[0]	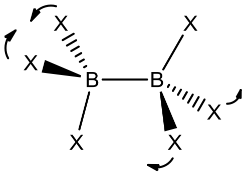<br>Out-of-phase BX_2 bend
**8**		398.2<br>(0.5) [0]	401.1<br>(0.8)[0]	
**9**		456.9<br>(0) [2.5]	459.1<br>(0)[2.3]	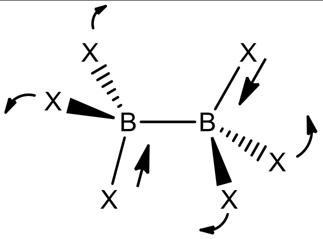<br>deformation
**10**		458.5<br>(0) [2.4]	460.2<br>(0)[2.3]	
**11**	563 i	544.5<br>(26.9) [0]	547.1<br>(27.9)[0]	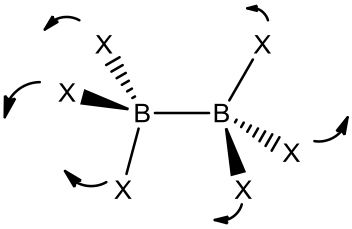<br>out-of-phase umbrella
**12**	626 r	601.8<br>(0) [7.1]	603.3<br>(0)[7.0]	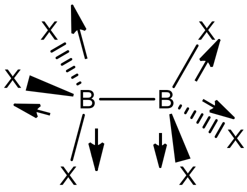<br>In-phase symmetric BX stretch
**13**	843i	800.1<br>(875.0) [0]	811.9<br>(887.1)[0]	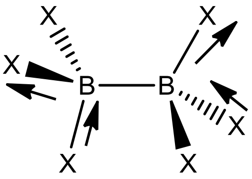<br>Out-of-phase asymetric B-X stretch
**14**		804.0<br>(871.6) [0]	815.9<br>(881.1)[0]	

**15**		838.8<br>(0) [3.4]	848.6<br>(0)[2.2]	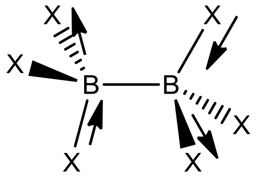
**16**		839.5<br>(0) [3.4]	850.6<br>(0)[2.2]	In-phase asymmetric B-X stretch
**17**	886(sh) i	841.8<br>(501) [0]	841.6<br>(523.0) [0]	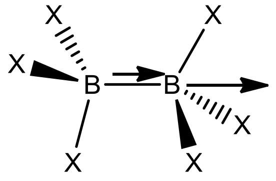<br>Out-of-phase symmetric B-X stretch
**18**		1173.5<br>(0) [63.3]	1192.5<br>(0) [20.6]	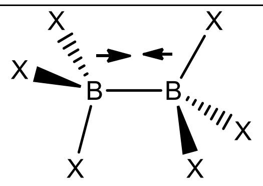<br>B-B stretch

Table S5: Predicted vibrational frequencies (cm^{-1}) for **2**.

**Mode #**	**Tentative experimental assignment (r = Raman; i = IR)**	**Predicted frequency (IR int.) [Raman int.]**		**Approximate description of vibrational mode**
		**B3lyp**	**ωb97xd**	
**1**		42.9 (0)[0]	64.8 (0) [0]	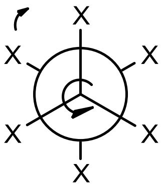 Torsion about B-B bond
**2**		99 (0.1) [0]	107.4 (0) [0]	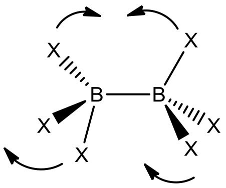 Rock
**3**		120.7 (0.6) [0]	125.5 (0.6) [0]	
**4**	175 r	156.6 (0) [2.3]	167.3 (0) [1.8]	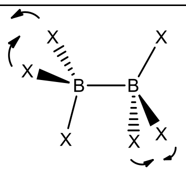 In-phase BX_2 bend
**5**		167.5 (0) [3.9]	176.8 (0) [3.1]	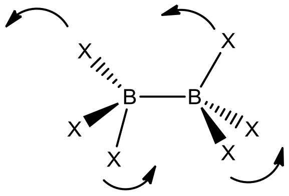 Deformation
**6**		172.5 (0) [2.9]	181.8(0) [3.4]	

**7**		214.8 (2.6) [0]	225.9 (1.1) [0]	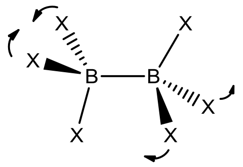 Out-of-phase BX_2 bend
**8**		226.2 (2.7) [0]	232.8 (0.9) [0]	
**9**	275 r	258.5 (0) [9.6]	268.6 (0) [7.8]	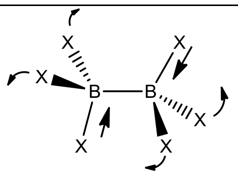 Deformation
**10**		261.1 (0) [9.6]	272.3 (0) [7.7]	
**11**		302.1 (0) [0]	310.5 (0.1) [0]	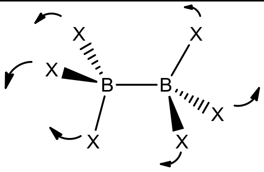 out-of-phase umbrella
**12**	354r	337.6 (0) [21.7]	351.0 (0.1) [19.9]	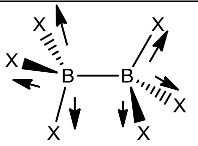 In-phase symmetric BX stretch
**13**	(554, 569, 591, 601) i	542.3 (265.7) [0]	559 (184.4) [0]	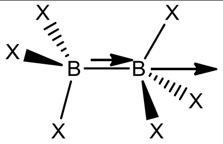 Out-of-phase symmetric B-X stretch
**14**		540.7 (643.9) [0]	583.8 (689.2) [0]	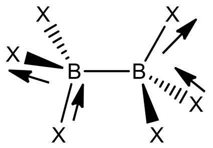 Out-of-phase asymmetric B-X stretch
**15**		543.1 (664.1) [0]	587.1 (686.5) [0]	

**16**	641r	588.2 (0)<br>[38.8]	627.1 (0)<br>[31.9]	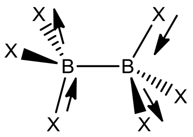<br>In-phase asymetric B-X stretch
**17**		589.9(0)<br>[38.7]	630.3 (0)<br>[31.8]	
**18**		985.2 (0)<br>[12.7]	1001.1 (0)<br>[14.7]	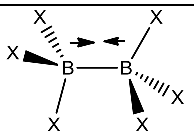<br>B-B stretch

Table S6: Predicted vibrational frequencies (cm^{-1}) for **3**.

**Mode #**	**Tentative experimental assignment (r = Raman; i = IR)**	**Predicted frequency (IR int.) [Raman int.]**		**Approximate description of vibrational mode**
		**B3lyp**	**ωb97xd**	
**1**		32.2<br>(0) [0]	46.4 (0) [0]	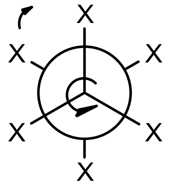<br>Torsion about B-B bond
**2**		64.6<br>(0) [0]	73.8<br>(0) [0]	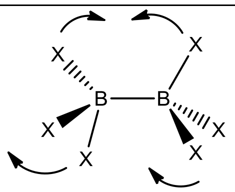<br>Rock
**3**		75.9<br>(0) [0]	81.7<br>(0) [0]	
**4**		100.5<br>(0) [2.3]	103.3<br>(0) [1.0]	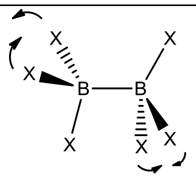<br>In-phase BX_2 bend

**5**		101.1 (0) [2.3]	107.2 (0) [2.0]	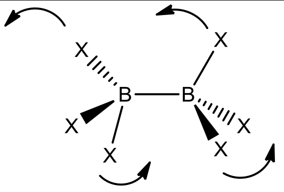 Deformation
**6**		103.4 (0) [0.7]	109.9 (0) [1.5]	
**7**		127.4 (2.6) [0]	135.6 (1.6) [0]	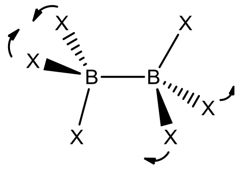 Out-of-phase BX_2 bend
**8**		134.2 (2.5) [0]	139.5 (1.5) [0]	
**9**	170 r	153.7 (0) [7.8]	164.1 (0) [5.7]	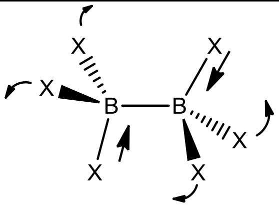 Deformation
**10**		157.0 (0) [7.8]	166.4 (0.7) [5.6]	
**11**		180.3 (1.1) [0]	187.20 (0.7) [0]	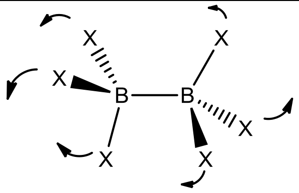 out-of-phase umbrella
**12**	214r	202.0 (0) [17.2]	211.5 (0) [15.3]	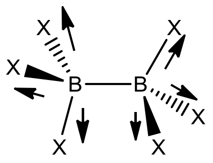 In-phase symmetric BX stretch
**13**		454.5 (106.8) [0]	475.0 (99.9) [0]	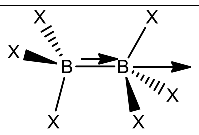 Out-of-phase symmetric B-X stretch

**14**	498 (sh) i	470.2<br>(497.6) [0]	516.3<br>(477.2) [0]	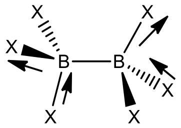<br>Out-of-phase asymmetric B-X stretch
**15**		471.0<br>(498.2) [0]	520.1<br>(480.3) [0]	
**16**	(560, 576)r	530.6<br>(0) [125.2]	575.1<br>(0) [92.5]	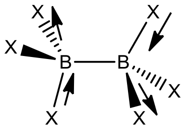<br>In-phase asymetric B-X stretch
**17**		531.2<br>(0) [125.2]	578.7<br>(0) [92.8]	
**18**		931.8<br>(0) [18.7]	946.6<br>(0) [23.8]	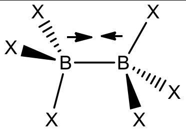<br>B-B stretch

Table S7: Predicted vibrational frequencies (cm^{-1}) for **4**.

**Mode #**	**Tentative experimental assignment (r = Raman; i = IR)**	**Predicted frequency (IR int.) [Raman int.]**		**Approximate description of vibrational mode**
		**B3lyp**	**ωb97xd**	
**1**		33.1 (0)[0]	42.1 (0)[0]	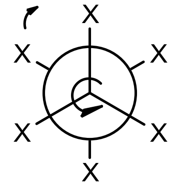 Torsion about B-B bond
**2**		56.7 (0.1)[0]	60.5 (0.1)[0]	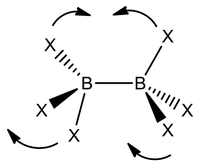 Rock
**3**		61.4 (0.1)[0]	63.7 (0.2)[0]	
**4**		76.5 (0)[9.8]	80.9 (0)[6.8]	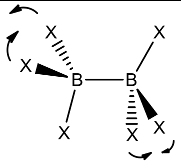 In-phase BX_2 bend
**5**		78.1 (0)[8.0]	82.2 (0)[8.7]	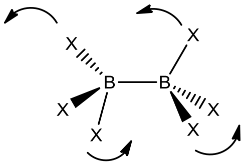 Deformation
**6**		79.0 (0)[9.0]	82.6 (0)[9.5]	

**7**		95.6<br>(1.7)[0]	102.8<br>(1.1)[0]	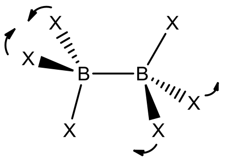<br>Out-of-phase BX_2 bend
**8**		98.5<br>(1.8)[0]	103.8<br>(1.1)[0]	
**9**	152 r	110.1<br>(0)[24.4]	120.6<br>(0)[18.5]	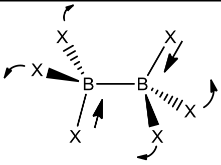<br>Deformation
**10**		112.1<br>(0)[24.9]	121.4<br>(0)[17.5]	
**11**		128.7<br>(1.0)[0]	134.8<br>(0.7)[0]	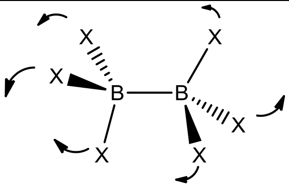<br>out-of-phase umbrella
**12**	200 r	142.3<br>(0)[46.1]	152.7<br>(0)[38.0]	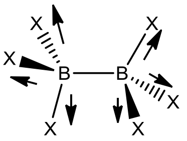<br>In-phase symmetric BX stretch

**13**	464 i	376.2<br>(157.4)[0]	416.6<br>(138.3)[0]	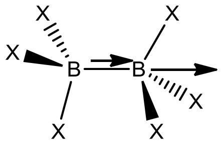<br>Out-of-phase symmetric B-X stretch
**14**		380.3<br>(581.0)[0]	428.2<br>(656.7)[0]	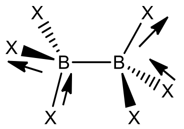<br>Out-of-phase asymmetric B-X stretch
**15**		384.2<br>(611.6)[0]	453.6<br>(652.0)[0]	
**16**	(486, 517, 534) r	444.7<br>(0)[252.9]	484.5<br>(0)[173.5]	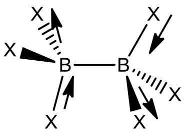<br>In-phase asymetric B-X stretch
**17**		447.3<br>(0)[249.9]	508.4<br>(0)[182.2]	
**18**	892 r	903.3<br>(0)[277.0]	926.1<br>(0)[180.6]	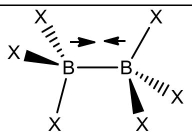<br>B-B stretch

X-ray Crystallographic Details

The crystal data for structures **$[PPh_4]_2[1]\cdot[PPh_4]Br\cdot CH_3CN$**, **$[PPh_4]_2[2]\cdot 2CH_2Cl_2$**, **$[PPh_4]_2[3]\cdot 2CH_2Cl_2$**, **$[PPh_4]_2[4]$**, **$[PPh_4]_2[2]\cdot 4CH_3CN$** and **$[PPh_4][B_2Br_5(CH_3CN)]$** were collected on a Bruker D8 Quest diffractometer with a CMOS area detector and multi-layer mirror monochromated $Mo_{K\alpha}$ radiation. The data for structures **$[PPh_4][BBr_4]$**, **$[PPN]_2[2]\cdot 4CH_2Cl_2$**, **$[PPh_4][BI_4]$** and **$[PPh_4]_2[4]\cdot 2CH_2Cl_2$** were acquired on a Bruker X8-APEX II diffractometer with a CCD area detector and multi-layer mirror monochromated $Mo_{K\alpha}$ radiation. The structure was solved using intrinsic phasing method[24] and refined with the ShelXL program[25] and expanded using Fourier techniques. All non-hydrogen atoms were refined anisotropically. Hydrogen atoms were included in structure factors calculations. All

hydrogen atoms were assigned to idealized geometric positions and are depicted as spheres of arbitrary radius. Non-hydrogen ellipsoids are depicted at the 50% probability level.

Crystallographic data have been deposited with the Cambridge Crystallographic Data Center as supplementary publication nos. CCDC 1916763-1916772. These data can be obtained free of charge from The Cambridge Crystallographic Data Centre *via* www.ccdc.cam.ac.uk/data_request/cif.

$[PPh_4]_2[B_2F_6]^{\cdot}[PPh_4]Br^{\cdot}CH_3CN$ ($[PPh_4]_2[1]^{\cdot}[PPh_4]Br^{\cdot}CH_3CN$)

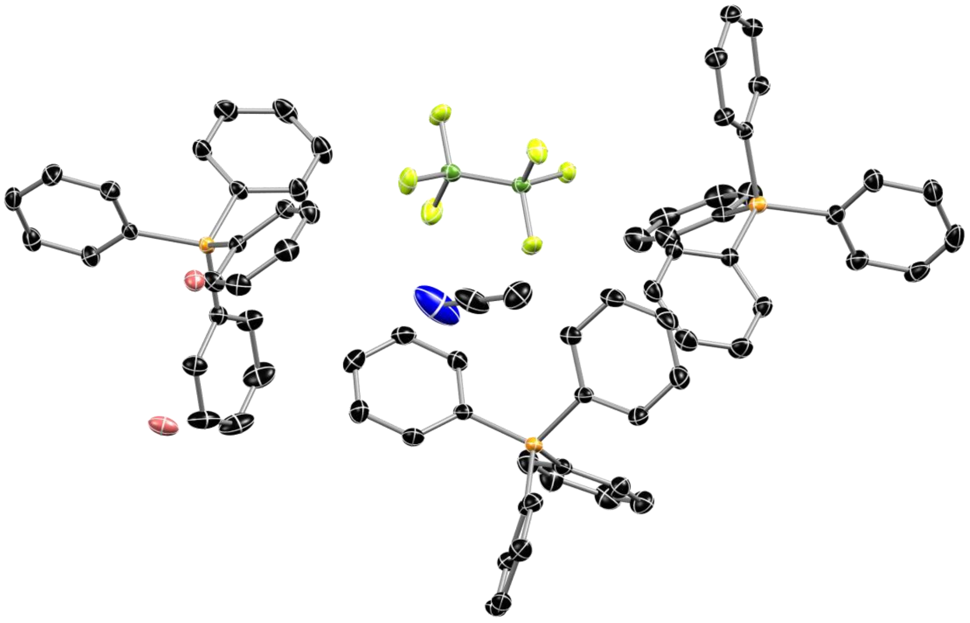

Figure S45: Solid-state structure of **$[PPh_4]_2[1]^{\cdot}[PPh_4]Br^{\cdot}CH_3CN$**, showing the asymmetric unit. Hydrogen atoms were omitted for clarity. The two bromide ions lie on a two-fold rotation axis and are therefore at half occupancy, whereas all of the other fragments are at full occupancy, in agreement with the expected charge balance.

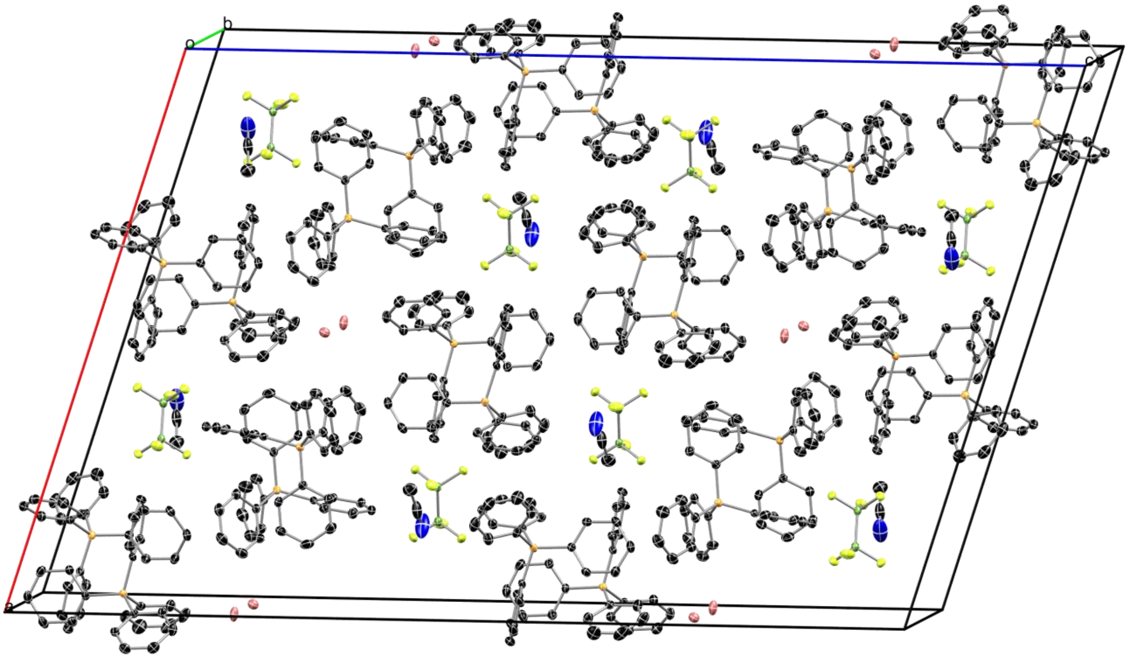

Figure S46: Packing in the solid-state structure of **[PPh$_4$]$_2$[1]$\cdot$[PPh$_4$]Br$\cdot$CH$_3$CN**, from the b-axis perspective. Hydrogen atoms were omitted for clarity.

Table S8: Sample and crystal data for **[PPh$_4$]$_2$[1]$\cdot$[PPh$_4$]Br$\cdot$CH$_3$CN.**

Data	**GuBC162_GuBC/final2_a.res**
Empirical formula	$C_{74}H_{63}B_2BrF_6NP_3$
Formula weight (g·mol^{-1})	1274.69
Temperature (K)	100(2)
Radiation, λ (Å)	0.71073
Crystal system	Monoclinic
Space group	$C2_1/c$
Unit cell dimensions	
a (Å)	27.934(5)
b (Å)	10.884(2)
c (Å)	42.694(10)
α (°)	90
β (°)	107.631(7)
γ (°)	90
Volume (Å^3)	12371.(4)
Z	8
Calculated density (Mg·m^{-3})	1.369
Absorption coefficient (mm^{-1})	0.806
F(000)	5264
Theta range for collection(°)	2.00 to 25.68
Reflections collected	45671
Independent reflections	11649 (R_{int} = 0.0728)

Maximum/minimum transmission	0.7454 and 0.6198
Refinement method	Full-matrix least-squares on F^2
Data / restraints/ parameters	11649/ 0 / 786
Goodness-of-fit on F^2	1.006
Final R indices [I>2σ(I)]	R1 = 0.0534, wR2 = 0.1057
R indices (all data)	R1 = 0.0829, wR2 = 0.1165
Maximum/minimum residual electron density (e·Å^{-3})	0.475 and −0.389

$[PPh_4]_2[B_2Cl_6]^{\cdot}2CH_2Cl_2$ ($[PPh_4]_2[2]^{\cdot}2CH_2Cl_2$)

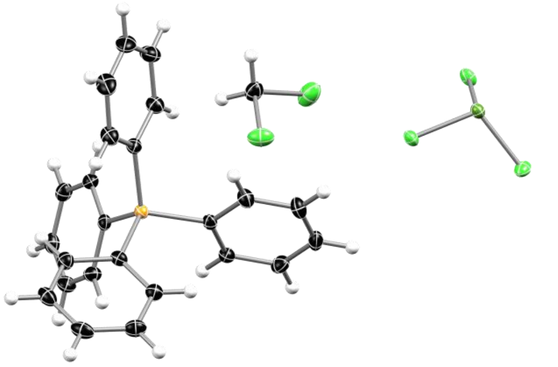

Figure S47: Solid-state structure of **$[PPh_4]_2[2]^{\cdot}2CH_2Cl_2$**, showing only the asymmetric unit. The $[B_2Cl_6]$ moiety lies on a an inversion center and the full dianion is generated by symmetry.

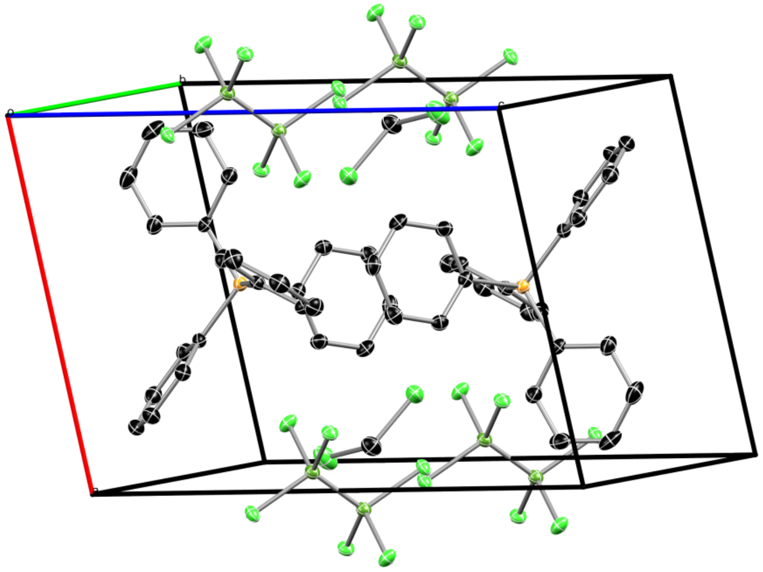

Figure S48: Packing in the solid-state structure of **$[PPh_4]_2[2]^{\cdot}2CH_2Cl_2$**, showing the symmetry-generated $[B_2Cl_6]$ moieties. The cell effectively "contains" four quarter-molecules of $[B_2Cl_6]$, two $[PPh_4]$ ions and two CH_2Cl_2 molecules. Hydrogen atoms were omitted for clarity.

Table S9: Sample and crystal data for **$[PPh_4]_2[2]^{\cdot}2CH_2Cl_2$**.

Data	**GuBC129_GuBC/final2_a.res**
Empirical formula	$C_{25}H_{22}BCl_5P$
Formula weight (g·mol^{-1})	541.45
Temperature (K)	100(2)
Radiation, λ (Å)	0.71073
Crystal system	Triclinic
Space group	$P\bar{1}$
Unit cell dimensions	
a (Å)	10.0512(6)
b (Å)	11.1533(6)
c (Å)	12.4741(7)
α (°)	69.286(2)
β (°)	78.975(2)
γ (°)	78.874(2)
Volume (Å^3)	1271.84(13)
Z	2
Calculated density (Mg·m^{-3})	1.414
Absorption coefficient (mm^{-1})	0.646
F(000)	554
Theta range for collection(°)	1.76 to 26.37
Reflections collected	33541
Independent reflections	5198 (R_{int} = 0.0556)
Maximum/minimum transmission	0.7454 and 0.7088
Refinement method	Full-matrix least-squares on F^2
Data / restraints/ parameters	5198/ 0 / 289
Goodness-of-fit on F^2	1.059
Final R indices [I>2σ(I)]	R1 =0.0309, wR2 = 0.0673
R indices (all data)	R1 =0.0368, wR2 =0.0706
Maximum/minimum residual electron density (e·Å^{-3})	0.676 and -0.594

$[PPN]_2[B_2Cl_6]^{\cdot}4CH_2Cl_2$ ($[PPN]_2[2]^{\cdot}4CH_2Cl_2$)

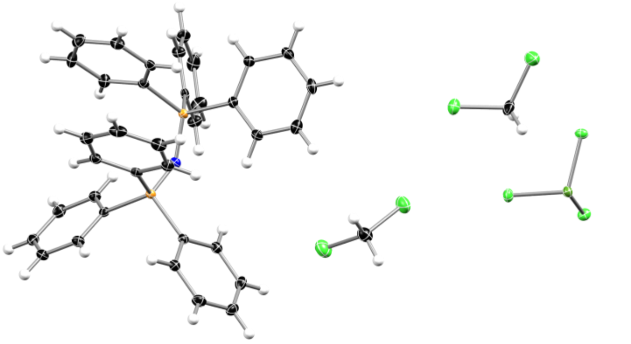

Figure S49: Solid-state structure of **$[PPN]_2[2]^{\cdot}4CH_2Cl_2$**, showing only the asymmetric unit. The $[B_2Cl_6]$ moiety lies on an inversion center and the full dianion is generated by symmetry. Selected bond distances (Å): B1-B1 1.703(3); B1-Cl1 1.907(2); B1-Cl2 1.896(2); B1-Cl3 1.895(3).

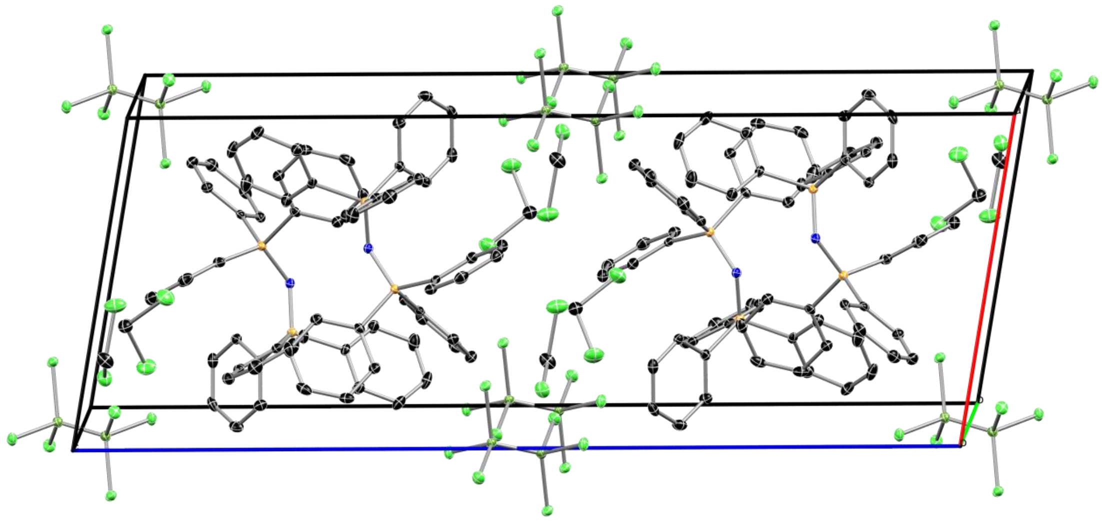

Figure S50: Packing in the solid-state structure of **$[PPN]_2[2]^{\cdot}4CH_2Cl_2$**, showing the symmetry-generated $[B_2Cl_6]$ moieties. The cell effectively "contains" eight quarter-molecules of $[B_2Cl_6]$, four [PPN] ions and eight CH_2Cl_2 molecules. Hydrogen atoms were omitted for clarity.

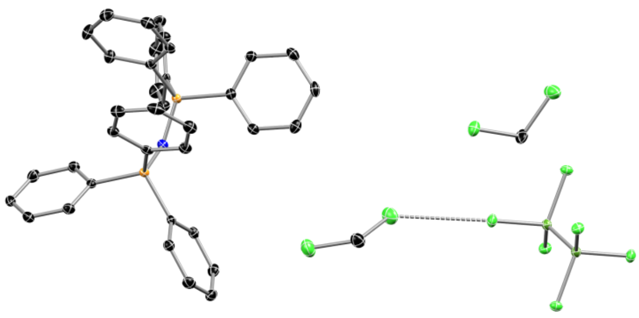

Figure S51: A noteworthy Cl-Cl short contact in the solid-state structure of **$[PPN]_2[2]^{\cdot}4CH_2Cl_2$** (Cl-Cl distance 3.3965(9) Å).

Table S10: Sample and crystal data for **$[PPN]_2[2]{\cdot}4CH_2Cl_2$**.

Data	**GuBC080_GuBC/final_a.res**
Empirical formula	$C_{38}H_{34}BCl_7NP_2$
Formula weight (g·mol^{-1})	825.56
Temperature (K)	100(2)
Radiation, λ (Å)	0.71073
Crystal system	Monoclinic
Space group	$P2_1/c$
Unit cell dimensions	
a (Å)	10.7243(4)
b (Å)	12.8555(5)
c (Å)	28.1347(13)
α (°)	90
β (°)	99.8150(10)
γ (°)	90
Volume (Å^3)	3822.1(3)
Z	4
Calculated density (Mg·m^{-3})	1.435
Absorption coefficient (mm^{-1})	0.633
F(000)	1692
Theta range for collection(°)	1.47 to 26.42
Reflections collected	31835
Independent reflections	7818 (R_{int} = 0.0544)
Maximum/minimum transmission	0.7454 /0.7014
Refinement method	Full-matrix least-squares on F^2
Data / restraints/ parameters	7818/ 0 / 442
Goodness-of-fit on F^2	1.013
Final R indices [I>2σ(I)]	R1 =0.0369, wR2 = 0.0834
R indices (all data)	R1 =0.0544, wR2 = 0.0911
Maximum/minimum residual electron density (e·Å^{-3})	0.691 and −0.407

$[PPh_4]_2[B_2Cl_6]^{\cdot}4CH_3CN$ ($[PPh_4]_2[2]^{\cdot}4CH_3CN$)

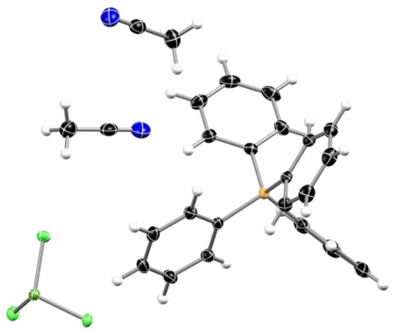

Figure S52: Solid-state structure of **$[PPh_4]_2[2]^{\cdot}4CH_3CN$**, showing only the asymmetric unit. The $[B_2Cl_6]$ moiety lies on a an inversion center and the full dianion is generated by symmetry. Selected bond distances (Å): B-B 1.710(2); B1-Cl3 1.898(1); B1-Cl1 1.890(1); B1-Cl2 1.909(1). Cl-B-B-Cl torsions angles (°): 60.2(1); –60.7(1); –180.00(6).

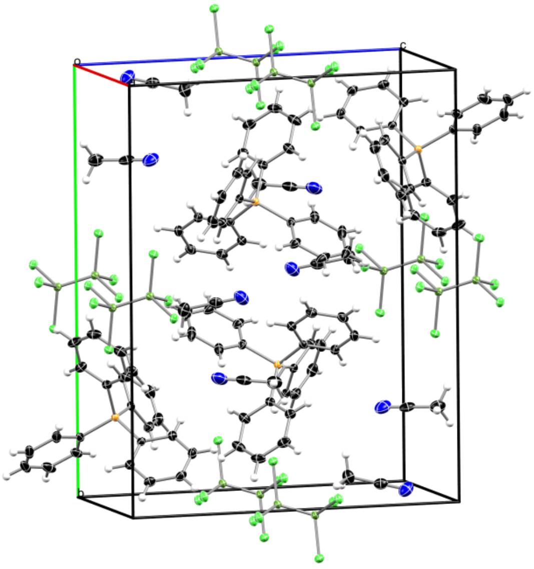

Figure S53: Packing in the solid-state structure of **$[PPh_4]_2[2]^{\cdot}4CH_3CN$**, showing the symmetry-generated $[B_2Cl_6]$ moieties. The cell effectively "contains" eight quarter-molecules of $[B_2Cl_6]$, four $[PPh_4]$ ions and eight CH_3CN molecules.

Table S11: Sample and crystal data for **$[PPh_4]_2[2]^{\cdot}4CH_3CN$**.

Data	**GuBC184_GuBC/final_a.res**
Empirical formula	$C_{56}H_{52}B_2Cl_6N_4P_2$,
Formula weight (g·mol^{-1})	1077.27
Temperature (K)	100(2)
Radiation, λ (Å)	0.71073
Crystal system	2

Space group	$P2_1/c$
Unit cell dimensions	
a (Å)	9.4064(4)
b (Å)	19.0107(8)
c (Å)	15.3590(6)
α (°)	90
β (°)	97.037(2)
γ (°)	90
Volume (Å^3)	2725.8(2)
Z	2
Calculated density (Mg·m^{-3})	1.313
Absorption coefficient (mm^{-1})	0.415
F(000)	1116
Theta range for collection(°)	2.43 to 26.40
Reflections collected	29053
Independent reflections	5593 (R_{int} = 0.0239)
Maximum/minimum transmission	0.8770/0.7280
Refinement method	Full-matrix least-squares on F^2
Data / restraints/ parameters	5593/ 0 / 318
Goodness-of-fit on F^2	1.037
Final R indices [I>2σ(I)]	R1 = 0.0241, wR2 = 0.0599
R indices (all data)	R1 = 0.0270, wR2 = 0.0620
Maximum/minimum residual electron density (e·Å^{-3})	0.470 and −0.258

$[PPh_4]_2[B_2Br_6]^{\cdot}2CH_2Cl_2$ ($[PPh_4]_2[3]^{\cdot}2CH_2Cl_2$)

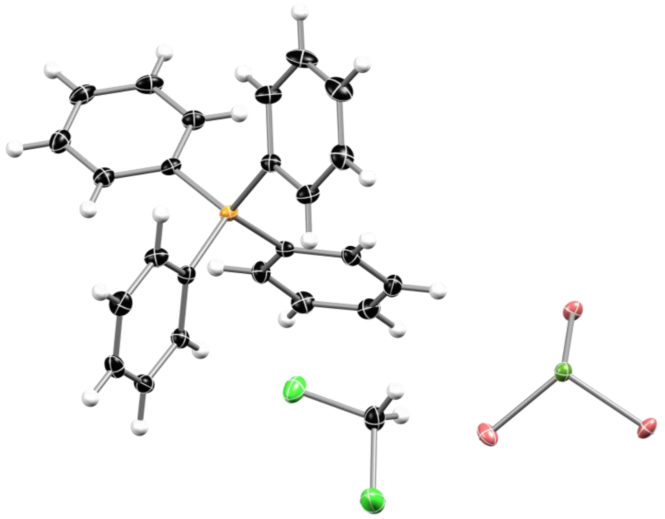

Figure S54: Solid-state structure of **$[PPh_4]_2[3]^{\cdot}2CH_2Cl_2$**, showing only the asymmetric unit. The $[B_2Br_6]$ moiety lies on an inversion center and the full dianion is generated by symmetry.

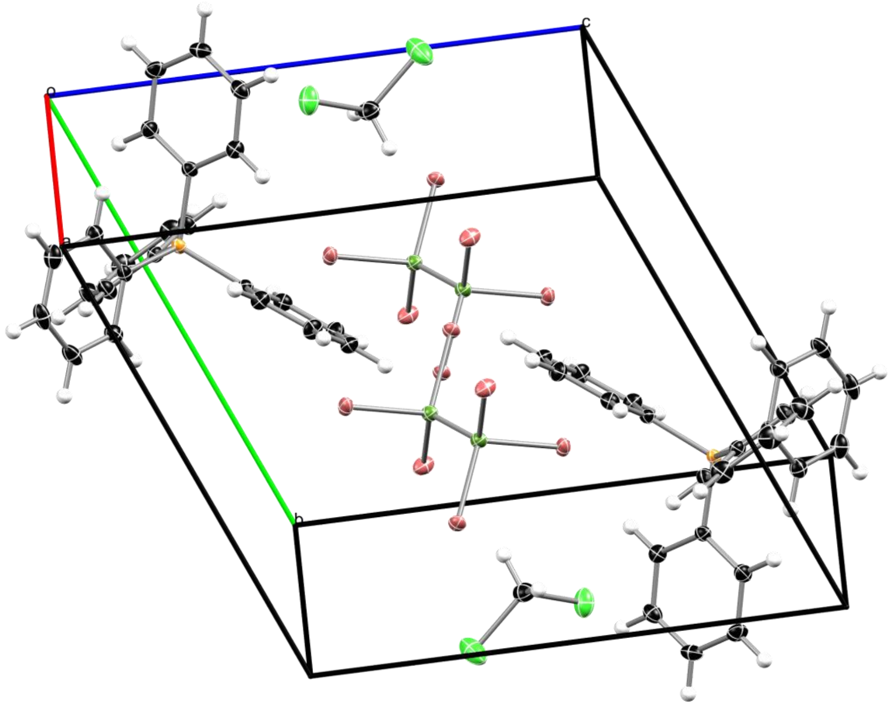

Figure S55: Packing in the solid-state structure of **[PPh$_4$]$_2$[3]·2CH$_2$Cl$_2$**, showing the symmetry-generated [B_2Br_6] moieties. The cell effectively "contains" two half-molecules of [B_2Br_6], two [PPh_4] ions and two CH_2Cl_2 molecules.

Table S12: Sample and crystal data for **[PPh$_4$]$_2$[3]·2CH$_2$Cl$_2$**.

Data	**GuBC044_GuBC/Final_a.res**
Empirical formula	$C_{25}H_{22}BBr_3Cl_2P$
Formula weight (g·mol^{-1})	674.83
Temperature (K)	100(2)
Radiation, λ (Å)	0.71073
Crystal system	Triclinic
Space group	$P\bar{1}$
Unit cell dimensions	
a (Å)	10.2440(7)
b (Å)	11.3481(8)
c (Å)	12.4910(8)
α (°)	69.238(2)
β (°)	80.399(2)
γ (°)	78.833(2)
Volume (Å^3)	
	1324.43(16)
Z	2
Calculated density (Mg·m^{-3})	1.692
Absorbtion coefficient (mm^{-1})	4.843
F(000)	662

Theta range for collection	2.54 to 27.10
Reflections collected	43272
Independent reflections	5771 [R(int) = 0.0429]
Minimum/maximum transmission	0.5962 and 0.4817
Refinement method	Full-matrix least-squares on F^2
Data / parameters / restrains	5771 / 0 / 289
Goodness-of-fit on F^2	1.064
Final R indices [I>2σ(I)]	R1 = 0.0218, wR2 = 0.0429
R indices (all data)	R1 = 0.0297, wR2 = 0.0451
Maximum/minimum residual electron density ($e \cdot Å^{-3}$)	0.690/ −0.808

$[PPh_4][B_2Br_5NCCH_3]$

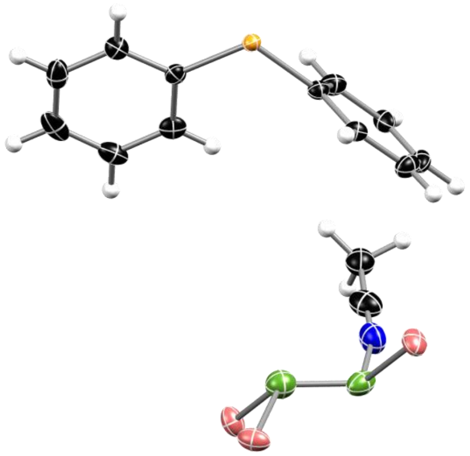

Figure S56: Solid-state structure of **$[PPh_4][B_2Br_5NCCH_3]$**, showing only the asymmetric unit. The $[PPh_4]$ ion lies on a two-fold rotation axis and the $[B_2Br_5(CH_3CN)]$ moiety partially lies on a mirror plane. The full ion pair is generated by symmetry.

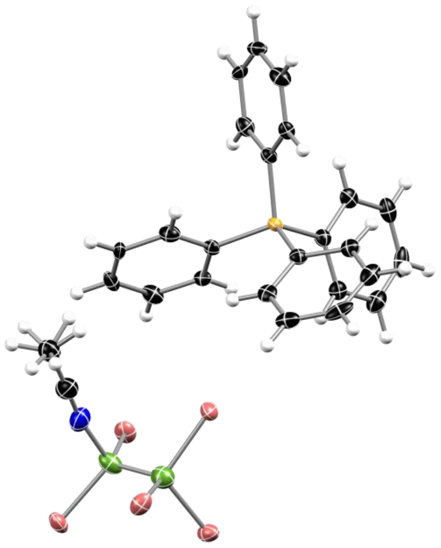

Figure S57: Solid-state structure of **[PPh_4][$B_2Br_5NCCH_3$]**, showing the full ion pair generated by symmetry and the two symmetry-generated orientations for the methyl group.

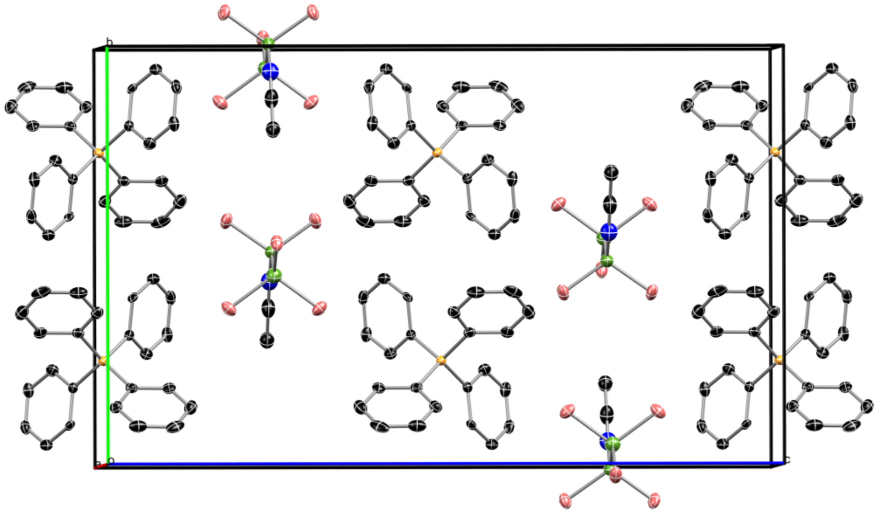

Figure S58: Packing in the solid-state structure of **[PPh_4][$B_2Br_5NCCH_3$]**, from the a-axis perspective. Hydrogen atoms were omitted for clarity.

The crystal was a pseudo-merohedral twin with a second domain rotated by 3.3° around real axis [–0.118 0.297 1.000] and a third domain rotated by 176.5° around real axis [–0.137 0.050 1.000]. The BASF parameters were refined to 32 and 15%. The structure was refined using the TWIN keyword. The model shows small signs of potential substitutional disorder that manifests itself as a rather large residual electron density peak in the vicinity of the acetonitrile ligand (which is also observed for crystals without twinning). The model as it stands is nevertheless of sufficient quality to confirm the existence of the **[$B_2Br_5NCCH_3$]** anion as suggested from our ^{11}B NMR results and quantum chemical calculations.

Table S13: Sample and crystal data for **[PPh_4][$B_2Br_5NCCH_3$]**.

Data	**GuBC186_GuBC/twin_a.res**
Empirical formula	$C_{26}H_{23}B_2Br_5NP$
Formula weight (g·mol^{-1})	801.54
Temperature (K)	100(2)
Radiation, λ (Å)	0.71073
Crystal system	Orthorhombic
Space group	*Pbcm*
Unit cell dimensions	
a (Å)	7.4355(2)
b (Å)	15.4193(5)
c (Å)	25.0317(8)
α (°)	90
β (°)	90
γ (°)	90
Volume (Å^3)	2869.89(15)
Z	4
Calculated density (Mg·m^{-3})	1.855

Absorption coefficient (mm^{-1})	7.074
F(000)	1544
Theta range for collection(°)	2.64 to 25.68
Reflections collected	3321
Independent reflections	2784(R_{sigma} = 0.0584)
Maximum/minimum transmission	0.745801/0.443496
Refinement method	Full-matrix least-squares on F^2
Data / restraints/ parameters	2784/ 0 / 171
Goodness-of-fit on F^2	1.049
Final R indices [I>2σ(I)]	R1 = 0.0616, wR2 = 0.1014
R indices (all data)	R1 = 0.0906, wR2 = 0.1125
Maximum/minimum residual electron density (e·$Å^{-3}$)	1.972 and −0.981

$[PPh_4]_2[B_2I_6]$ ($[PPh_4]_2$[4])

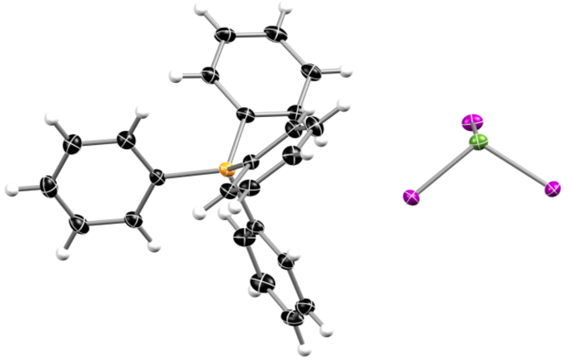

Figure S59: Solid-state structure of **$[PPh_4]_2$[4]**, showing only the asymmetric unit. The $[B_2I_6]$ moiety lies on an inversion center and the full dianion is generated by symmetry.

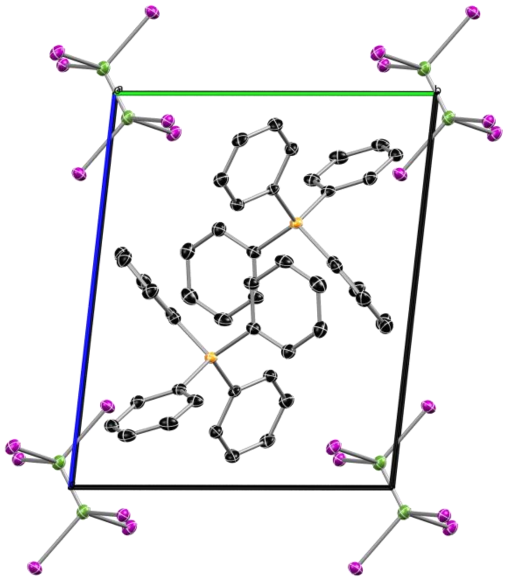

Figure S60: Packing in the solid-state structure of **[PPh$_4$]$_2$[4]**, showing the symmetry-generated [B_2I_6] moieties. The cell effectively "contains" four quarter-molecules of [B_2I_6] and two [PPh_4] ions. Hydrogen atoms were omitted for clarity.

Table S14: Sample and crystal data for **[PPh$_4$]$_2$[4]**.

Data	**GuBC154_GuBC/final_a.res**
Empirical formula	$C_{24}H_{20}BI_3P$
Formula weight (g·mol^{-1})	730.88
Temperature (K)	105(2)
Radiation, λ (Å)	0.71073
Crystal system	Triclinic
Space group	$P\bar{1}$
Unit cell dimensions	
a (Å)	10.1795(5)
b (Å)	10.9548(5)
c (Å)	12.4301(5)
α (°)	90.435(2)
β (°)	102.036(2)
γ (°)	115.6950(10)
Volume (Å^3)	124.03(10)
Z	2
Calculated density (Mg·m^{-3})	1.999
Absorption coefficient (mm^{-1})	3.936
F(000)	686
Theta range for collection(°)	2.08 to 26.37
Reflections collected	30446
Independent reflections	4895 (R_{int} = 0.0417)
Maximum/minimum transmission	0.6746/0.4932
Refinement method	Full-matrix least-squares on F^2
Data / restraints/ parameters	4895/ 0 /262
Goodness-of-fit on F^2	1.079
Final R indices [I>2σ(I)]	R1 = 0.0238, wR2 = 0.0587
R indices (all data)	R1 = 0.0264, wR2 = 0.0604
Maximum/minimum residual electron density (e·Å^{-3})	1.568 and -1.172

[PPh$_4$]$_2$[B_2I_6]$\cdot$2CH$_2$Cl$_2$ ([PPh$_4$]$_2$[4]$\cdot$2CH$_2$Cl$_2$)

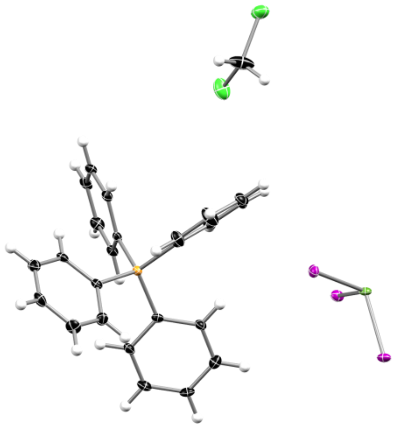

Figure S61: Solid-state structure of **$[PPh_4]_2[4]^{\cdot}2CH_2Cl_2$**, showing only the asymmetric unit. The $[B_2I_6]$ moiety lies on an inversion center and the full dianion is generated by symmetry.

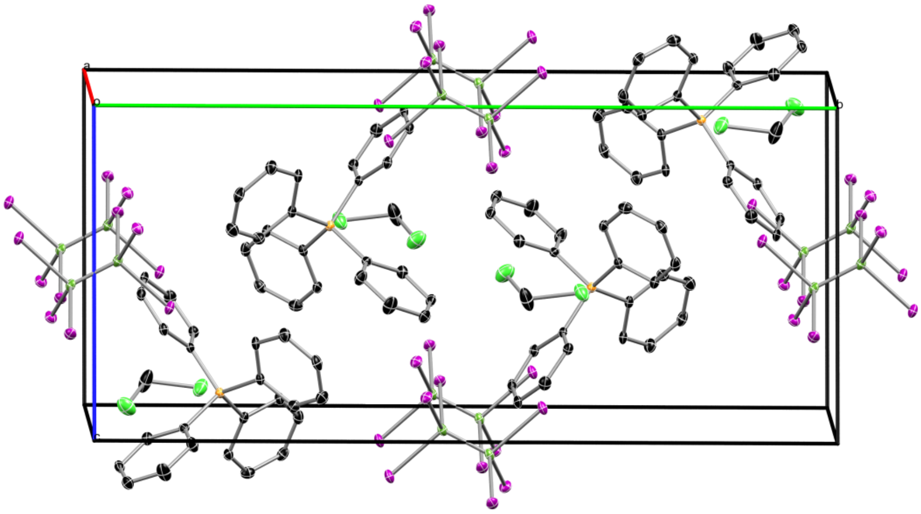

Figure S62: Packing in the solid-state structure of **$[PPh_4]_2[4]^{\cdot}2CH_2Cl_2$**, showing the symmetry-generated $[B_2I_6]$ moieties. The cell effectively "contains" eight quarter-molecules of $[B_2I_6]$, four $[PPh_4]$ ions and four DCM molecules. Selected bond distances (Å): B-B 1.700(5); B1-I3 2.275(3); B1-I1 2.291(3); B1-I2 2.260(4). I-B-B-I torsions angles (°): 58.7(2); –60.2(2); –180.0(1). Hydrogen atoms were omitted for clarity.

Table S15: Sample and crystal data for **$[PPh_4]_2[4]^{\cdot}2CH_2Cl_2$**.

Data	**GuBC200/final_a.res**
Empirical formula	$C_{25}H_{22}BCl_2I_3P$
Formula weight (g·mol^{-1})	815.80
Temperature (K)	100(2)
Radiation, λ (Å)	0.71073
Crystal system	Monoclinic
Space group	$P2_1/c$
Unit cell dimensions	
a (Å)	11.1381(5)
b (Å)	23.7609(8)
c (Å)	10.9057(5)
α (°)	90
β (°)	106.5410

γ (°)	90
Volume (Å^3)	2766.8(2)
Z	4
Calculated density (Mg·m^{-3})	1.958
Absorption coefficient (mm^{-1})	3.652
F(000)	1540
Theta range for collection(°)	1.71 to 26.43
Reflections collected	30222
Independent reflections	5679 (R_{int} = 0.0514)
Maximum/minimum transmission	0.4908/ 0.3383
Refinement method	Full-matrix least-squares on F^2
Data / restraints/ parameters	5679/ 0 /289
Goodness-of-fit on F^2	1.034
Final R indices [I>2σ(I)]	R1 = 0.0243, wR2 = 0.0527
R indices (all data)	R1 = 0.0308, wR2 = 0.0549
Maximum/minimum residual electron density (e·Å^{-3})	0.914 and −0.525

[PPh_4][BBr_4]

Crystals were obtained from the dichloromethane supernatant of the reaction of B_2Br_4 with [PPh_4]Br (yielding insoluble [PPh_4]$_2$[**3**].

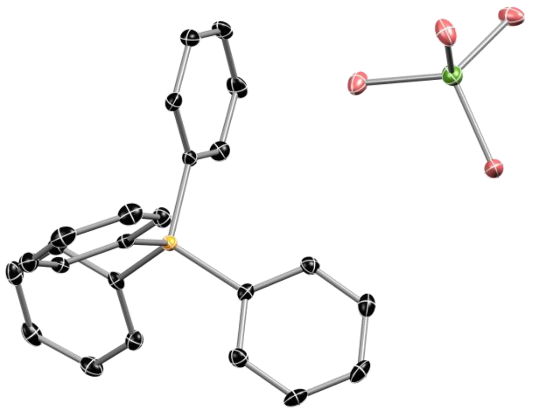

Figure S63: Solid-state structure of **[PPh_4][BBr_4]** showing the ion pair generated by symmetry (both ions lie on both four-fold rotoinversion and two-fold rotation axes). B-Br distance (Å): 2.0179(4). Hydrogen atoms were omitted for clarity.

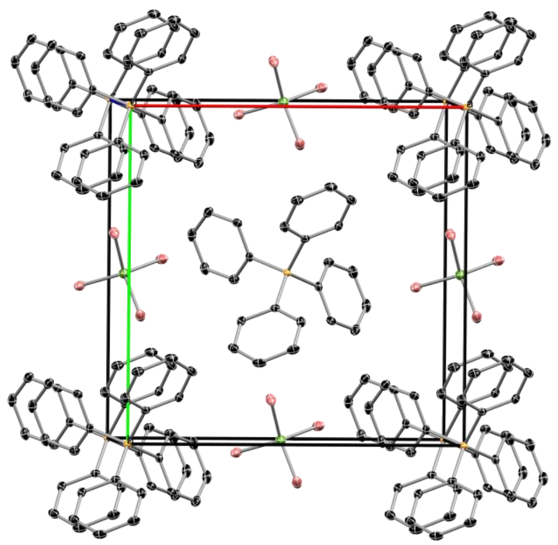

Figure S64: Packing in the solid-state structure of **[PPh$_4$][BBr$_4$]** from the c-axis perspective. Hydrogen atoms were omitted for clarity.

Table S16: Sample and crystal data for **[PPh$_4$][BBr$_4$]**

Data	**GuBC052_GuBC/final2_a.res**
Empirical formula	$C_{24}H_{20}BBr_4P$
Formula weight (g·mol^{-1})	669.82
Temperature (K)	100(2)
Radiation, λ (Å)	0.71073
Crystal system	Tetragonal
Space group	I-4
Unit cell dimensions	
a (Å)	12.9640(9)
b (Å)	12.9640(9)
c (Å)	7.0663(5)
α (°)	90
β (°)	90
γ (°)	90
Volume (Å^3)	1187.60(19)
Z	2
Calculated density (Mg·m^{-3})	1.873
Absorbtion coefficient (mm^{-1})	6.855
F(000)	648
Theta range for collection	2.22 to 26.34
Reflections collected	2865
Independent reflections	1202 Rint=0.0233
Minimum/maximum transmission	0.3633 and 0.2585
Refinement method	Full-matrix least-squares on F^2
Data / restraints/ parameters	1202 / 0 / 68
Goodness-of-fit on F^2	0.916

Final R indices [I>2σ(I)]	R1 = 0.0199, wR2 = 0.0412
R indices (all data)	R1 = 0.0218, wR2 = 0.0418
Maximum/minimum residual electron density (e·Å^{-3})	0.377 and -0.285

[PPh_4][BI_4]

Crystals were obtained from the toluene/DCM supernatant in the synthesis of bulk [PPh_4][**4**] by precipitation.

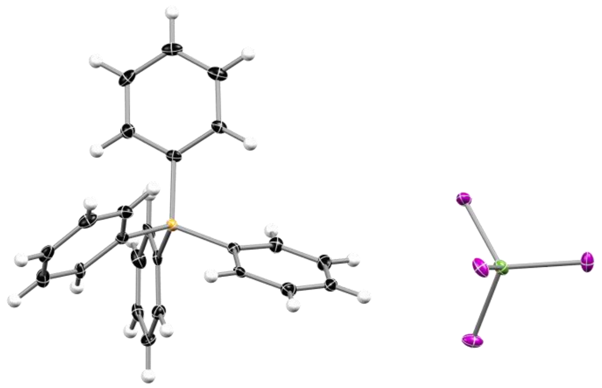

Figure S65: Solid-state structure of **[PPh_4][BI_4]** showing the ion pair generated by symmetry (both ions lie on both four-fold rotoinversion and two-fold rotation axes). B-I bond distance (Å): 2.2336(3).

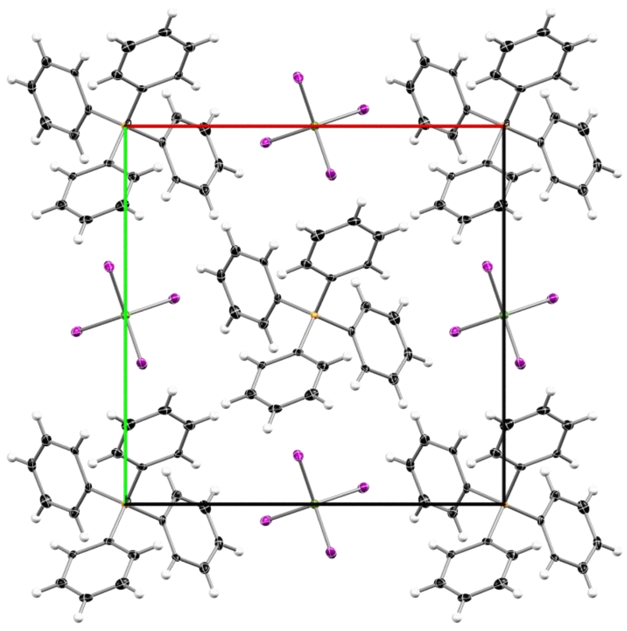

Figure S66: Packing in the solid-state structure of **[PPh_4][BI_4]** from the c-axis perspective.

Table S17: Sample and crystal data for **[PPh_4][BI_4]**

Data	**GuBC149_GuBC/sad_a.res**
Empirical formula	$C_{24}H_{20}BI_4P$
Formula weight (g·mol^{-1})	857.78
Temperature (K)	100(2)
Radiation, λ (Å)	0.71073
Crystal system	Tetragonal
Space group	$I\bar{4}$
Unit cell dimensions	
a (Å)	13.4868(13)
b (Å)	13.4868(13)
c (Å)	7.0506(7)
α (°)	90
β (°)	90
γ (°)	90
Volume (Å^3)	1282.5(3)
Z	2
Calculated density (Mg·m^{-3})	2.221
Absorption coefficient (mm^{-1})	4.932
F(000)	792
Theta range for collection(°)	3.02 to 26.37
Reflections collected	1795
Independent reflections	1183 (R_{int} =0.0189)
Maximum/minimum transmission	0.3494/0.2918
Refinement method	Full-matrix least-squares on F^2
Data / restraints/ parameters	1183/ 0 / 68
Goodness-of-fit on F^2	1.079
Final R indices [I>2σ(I)]	R1 =0.0170, wR2 = 0.0399
R indices (all data)	R1 = 0.0171, wR2 = 0.0400
Maximum/minimum residual electron density (e·Å^{-3})	0.359 and -0.438

Cartesian Coordinates of Optimized Structures

Cartesian coordinates (Å) and energy (a.u) of the stationary points calculated at the ωb97XD/6-311+g(d,p)-SMD-PCM level of theory (iodine-containing molecules at the ωb97xd/6-311+g(d,p)/lanl2dz-SMD-PCM level).

[B_2F_6]$^{2-}$ (1)

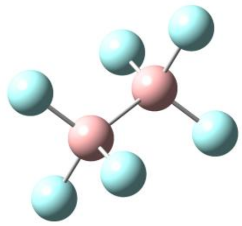

E= -649.3872767

B	-0.87167100	-0.00006500	-0.00030800
B	0.87167100	0.00006500	0.00030800
F	1.46351500	-1.03321700	0.84595100
F	1.46540400	-0.21576900	-1.31645300
F	1.46322400	1.24949300	0.47219300
F	-1.46540500	0.21576400	1.31645300
F	-1.46351500	1.03322100	-0.84594700
F	-1.46322300	-1.24949100	-0.47219800

$[B_2F_5]^-$

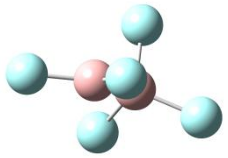

E= -549.3703963

B	1.01928500	0.00410200	-0.00845300
B	-0.71653600	0.00411200	-0.00474800
F	1.77110300	-1.11679100	-0.00324100
F	1.78425900	1.11521700	-0.00294000
F	-1.25410900	1.25104600	-0.45541600
F	-1.22469300	-0.22867800	1.31829300
F	-1.24475300	-1.02535800	-0.84936200

B_2F_4

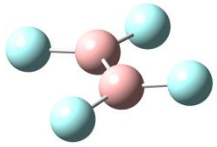

E=-449.3151123

B	0.00000000	0.00000000	0.85710100
B	0.00000000	0.00000000	-0.85710100
F	-0.06824000	1.12480600	1.55892700
F	0.06824000	-1.12480600	1.55892700
F	-0.06824000	-1.12480600	-1.55892700
F	0.06824000	1.12480600	-1.55892700

$[B_2F_5(NCCH_3)]^-$

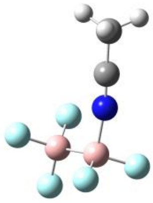

E= -682.12502

B	-0.28841500	0.88471500	-0.00033900
B	-1.26234300	-0.55816500	-0.00084400
F	-0.35656600	1.71873600	-1.15066600
F	-0.36239700	1.71985800	1.14930100
F	-1.10035000	-1.34451800	-1.20806500
F	-0.94363000	-1.45576100	1.09403800
N	1.30384400	0.40290800	0.00376500
C	2.33081500	-0.09895200	0.00171600
C	3.62627300	-0.73330600	0.00024500
H	3.49650400	-1.80592600	-0.15379200
H	4.23046800	-0.31313900	-0.80541000
H	4.11297300	-0.55346600	0.96029300
F	-2.67656800	-0.28131800	0.11169300

BF_3

E= -324.5700343

B	0.00000000	0.00000000	-0.00012500
F	0.00000000	0.00000000	1.31782400
F	0.00000000	1.14132400	-0.65887700
F	0.00000000	-1.14132400	-0.65887700

$[BF_4]^-$

E= -424.6366535

B	0.00000000	0.00000000	0.00000000
F	0.81513500	0.81513500	0.81513500
F	-0.81513500	-0.81513500	0.81513500
F	-0.81513500	0.81513500	-0.81513500
F	0.81513500	-0.81513500	-0.81513500

F^-

E= -99.9837649

$[B_2Cl_6]^{2-}$ (**2**)

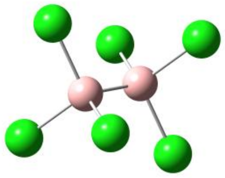

E=-2811.506198

B	0.86833800	0.00016000	0.00029200
B	-0.86833400	-0.00007800	-0.00015500
Cl	-1.59878900	0.15949700	1.76284400
Cl	-1.60050000	1.44795200	-1.01908800
Cl	1.59908800	1.60937000	0.74132700
Cl	1.60047200	-1.44737500	1.01998100
Cl	1.59854200	-0.16066500	-1.76265700
Cl	-1.59881400	-1.60880300	-0.74244600

$[B_2Cl_5]^-$

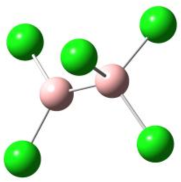

E= -2351.1169233

B	-1.00475300	-0.00000200	-0.11721300
B	0.70398100	0.00000000	-0.02801800
Cl	1.05161900	0.00002600	1.84534500
Cl	1.48583000	-1.52800600	-0.79864200
Cl	-1.96740600	-1.49617400	-0.10265300
Cl	-1.96740700	1.49616900	-0.10265100
Cl	1.48582600	1.52798600	-0.79868500

B_2Cl_4

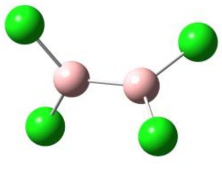

E= -1890.6961689

B	-0.84707500	-0.00000100	0.00000100
B	0.84707400	0.00000100	0.00000200
Cl	1.73631400	-1.12202000	1.01368300
Cl	1.73630900	1.12202400	-1.01368300
Cl	-1.73630700	-1.12202500	-1.01368300
Cl	-1.73631500	1.12202100	1.01368100

$[B_2Cl_5(NCCH_3)]^-$

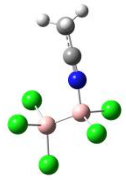

E= -2483.8907439

B	-0.27247400	0.77332400	0.00083500
Cl	-0.26686900	1.86261400	-1.54209800
Cl	0.72700300	-1.59389000	1.54950600
Cl	2.67986000	0.17028600	-0.03819600
Cl	0.67735900	-1.63704300	-1.51461400
B	0.91204500	-0.49393700	-0.00130800
N	-1.68899500	0.12145700	0.01485200
C	-2.67629800	-0.45223600	0.01431200
C	-3.91803300	-1.18079400	0.01093300
H	-4.40577500	-1.05570200	0.97937200
H	-4.55944100	-0.78921800	-0.78072100
H	-3.70930800	-2.23733600	-0.16679800

$B_2Cl_4(NCCH_3)_2$

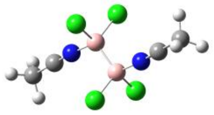

E= -2156. 2628108

B	-0.45788800	0.73763300	0.00864800
Cl	-0.17772200	1.78462500	1.54621600
Cl	0.16153100	-1.79070800	-1.52277700
Cl	0.17742300	-1.78469700	1.54610400
B	0.45788500	-0.73757700	0.00867500
N	1.96465800	-0.38239600	-0.00136800
C	3.05140500	-0.03144300	-0.01494300
C	4.42008200	0.41022300	-0.03594000

H	4.88468500	0.17293900	0.92294800
H	4.94558200	-0.10435100	-0.84259300
H	4.44235400	1.48843500	-0.20470700
N	-1.96465300	0.38240100	-0.00167200
C	-3.05137700	0.03137700	-0.01534500
C	-4.42002100	-0.41041700	-0.03607900
H	-4.88404300	-0.17424500	0.92338900
H	-4.94611000	0.10512800	-0.84171800
H	-4.44229300	-1.48845000	-0.20591100
Cl	-0.16127500	1.79088600	-1.52265400

BCl_3

E= -1405.6019293

B	0.00000000	0.00011800	0.00000000
Cl	0.00000000	-1.74831800	0.00000000
Cl	1.51404700	0.87414100	0.00000000
Cl	-1.51404700	0.87414200	0.00000000

$[BCl_4]^-$

E= -1866.0215305

B	0.00000000	0.00000000	0.00000000
Cl	1.07987300	1.07987300	1.07987300
Cl	-1.07987300	-1.07987300	1.07987300
Cl	-1.07987300	1.07987300	-1.07987300
Cl	1.07987300	-1.07987300	-1.07987300

Cl^-

E= -460.3792038

$[B_2Br_6]^{2-}$ (**3**)

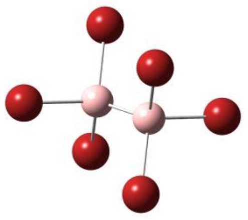

E= -15495.2616801

B	-0.87157600	-0.00024100	-0.00082500
B	0.87157800	0.00026100	0.00081500
Br	-1.67172500	1.82856300	0.59736000
Br	-1.67231300	-1.43305200	1.28267100
Br	-1.66819800	-0.39579400	-1.88442800
Br	1.66816900	0.39458500	1.88469200
Br	1.67236400	1.43386700	-1.28177300
Br	1.67170200	-1.82817300	-0.59852100

$[B_2Br_5]^-$

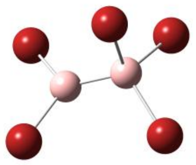

E= -12920.9208754

Br	-2.06054700	-1.61451300	-0.16268300
B	-0.99550700	0.00956900	-0.15030900
Br	-2.04485900	1.64301700	-0.17261600
Br	0.95954400	-0.05678200	2.02495400
B	0.70849800	-0.00161200	-0.03334300
Br	1.57964400	-1.64444700	-0.87957100
Br	1.60721900	1.67158800	-0.78384800

B_2Br_4

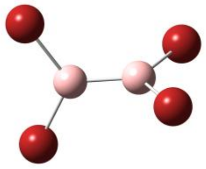

E= -10346.540473

Br	-1.78847300	-1.17266600	-1.17129300
B	-0.84123200	-0.00006500	0.00004400
Br	-1.78827100	1.17266100	1.17143600
Br	1.78849500	-1.17192800	1.17201700
B	0.84122900	-0.00004900	-0.00002500
Br	1.78825000	1.17194900	-1.17216200

BBr_3

E= -7747.4824665

B	0.00000000	0.00000000	0.00000000
Br	0.00000000	1.90654100	0.00000000
Br	-1.65111300	-0.95327000	0.00000000
Br	1.65111300	-0.95327000	0.00000000

$[BBr_4]^{-}$

E= -10321.8607496

B	0.00000000	0.00000000	0.00000000
Br	1.17668200	1.17668200	1.17668200
Br	-1.17668200	-1.17668200	1.17668200
Br	-1.17668200	1.17668200	-1.17668200
Br	1.17668200	-1.17668200	-1.17668200

Br^{-}
E= -2574.3401905

$[B_2I_6]^{2-}$ (4)

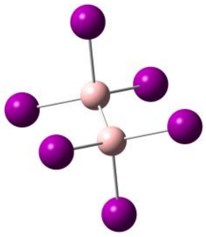

E= -118.5303432

B	0.86707700	-0.00266200	-0.00084500
B	-0.86712200	0.00252500	0.00093700
I	1.74601600	-2.10899600	-0.38879500
I	1.75576700	0.71254900	2.01525000
I	1.75546000	1.38506400	-1.62889400
I	-1.75581000	-0.71142000	-2.01580100
I	-1.74596600	2.10890400	0.39014600
I	-1.75546200	-1.38608800	1.62808600

$[B_2I_5]^{-}$

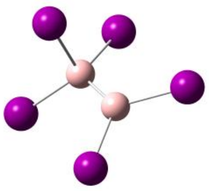

E= -106.9546136

B	0.98644800	0.00000200	-0.21046500
B	-0.70430100	-0.00000700	-0.04723100
I	-0.83259100	-0.00109200	2.26693400
I	-1.76051700	1.83816200	-0.86007200
I	2.16379200	-1.81934200	-0.26058500
I	2.16324300	1.81984200	-0.26039000
I	-1.76054500	-1.83756800	-0.86157600

B_2I_4

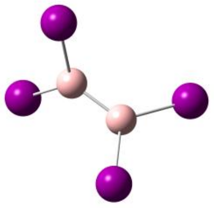

E= -95.3633037

B	0.00000000	0.00000000	-0.83238600
B	0.00000000	0.00000000	0.83238600
I	-1.31611700	-1.31621800	1.87113100
I	1.31611700	1.31621800	1.87113100
I	-1.31611700	1.31621800	-1.87113100
I	1.31611700	-1.31621800	-1.87113100

BI_3

E= -59.0941776

B	0.00000000	0.00000000	-0.00099200
I	0.00000000	0.00000000	2.13036200
I	0.00000000	1.84599200	-1.06513400
I	0.00000000	-1.84599200	-1.06513400

$[BI_4]^-$

E= -70.6852458

B	0.00000000	0.00000000	0.00000000
I	1.31227700	1.31227700	1.31227700
I	-1.31227700	-1.31227700	1.31227700
I	-1.31227700	1.31227700	-1.31227700
I	1.31227700	-1.31227700	-1.31227700

I^-

E= -11.5710274

CH_3CN

E= -132.7523594

N	1.42838300	0.00025600	-0.00001000
C	0.27772800	-0.00065500	0.00005400
C	-1.17364800	0.00011500	-0.00005600
H	-1.54006000	0.93936200	-0.41743900
H	-1.54177500	-0.83057900	-0.60414700
H	-1.54132500	-0.10733900	1.02167000

$BCl_3 \cdot SMe_2$

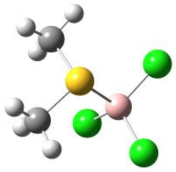

E= -1883.6558278

S	1.23977700	-0.00020600	-0.76848800
C	2.07552100	-1.39683500	0.01484000
H	1.56283500	-2.30630300	-0.29648500
H	3.10280300	-1.41097000	-0.34846500
H	2.04747600	-1.28930500	1.09858900
C	2.08313400	1.39076000	0.01683900
H	3.13307500	1.34154900	-0.27134700
H	1.63821500	2.30749200	-0.36774100
H	1.97454400	1.33150800	1.09941900
B	-0.59029900	0.00114600	0.03418800
Cl	-0.48115000	-0.04132000	1.87160300
Cl	-1.37141000	1.55405100	-0.57801900
Cl	-1.40013500	-1.50920000	-0.64530100

References

[1] J. Rohonczy, SOLA – Solid Lineshape Analysis Version 2.2.4, Bruker Biospin, Rheinstetten, Germany 2013.

[2] A. Finch, H. I. Schlesinger, *J. Am. Chem. Soc.* **1958**, *80*, 3573-3574.

[3] G. Urry, T. Wartik, R. E. Moore, H. I. Schlesinger, *J. Am. Chem. Soc.* **1954**, *76*, 5293-5298.

[4] H. Nöth, H. Pommerening, *Chem. Ber.* **1981**, *114*, 398-399.

[5] W. Haubold, P. Jacob, *Z. Anorg. Allg. Chem.* **1983**, *507*, 231-234.

[6] M. Arrowsmith, J. Böhnke, H. Braunschweig, A. Deißenberger, R. D. Dewhurst, W. C. Ewing, C. Hörl, J. Mies, J. H. Muessig, *Chem. Commun.* **2017**, *53*, 8265-8267.

[7] T. Shimanouchi, Tables of Molecular Vibrational Frequencies Consolidated Volume I, National Bureau of Standards, 1972, 1-160.

[8] J. A. Creighton, *J. Chem. Soc.* **1965**, 6589-6591.

[9] W. Haubold, U. Kraatz, W. Einholz, *Z. Anorg. Allg. Chem.* **1991**, *592*, 35-41.

[10] a) H. Binder, R. Kellner, K. Vaas, M. Hein, F. Baumann, M. Wanner, R. Winter, W. Kaim, W. Hönle, Y. Grin, U. Wedig, M. Schultheiss, R. K. Kremer, H. G. v. Schnering, O. Groeger, G. Engelhardt, *Z. Anorg. Allg. Chem.* **1999**, *625*, 1059-1072;

b) H. Binder, R. Kellner, K. Vaas, M. Hein, F. Baumann, M. Wanner, W. Kaim, U. Wedig, W. Hönle, H. G. von Schnering, O. Groeger, G. Engelhardt, *Z. Anorg. Allg. Chem.* **1999**, *625*, 1638-1646; c) W. Hönle, Y. Grin, A. Burkhardt, U. Wedig, M. Schultheiss, H. G. von Schnering, R. Kellner, H. Binder, *J. Solid State Chem.* **1997**, *133*, 59-67; d) W. Bowden, *J. Electrochem. Soc.* **1982**, *129*, 1249-1252; e) E. H. Wong, *Inorg. Chem.* **1981**, *20*, 1300-1302; f) W. H. Knoth, H. C. Miller, J. C. Sauer, J. H. Balthis, Y. T. Chia, E. L. Muetterties, *Inorg. Chem.* **1964**, *3*, 159-167.

[11] a) G. A. Olah, W. S. Tolgyesi, *J. Org. Chem.* **1961**, *26*, 2319-2323; b) T. C. Waddington, J. A. White, *Proc. Chem. Soc.* **1960**, 315.

[12] M. J. Frisch, G. W. Trucks, H. B. Schlegel, G. E. Scuseria, M. A. Robb, J. R. Cheeseman, G. Scalmani, V. Barone, B. Mennucci, G. A. Petersson, H. Nakatsuji, M. Caricato, X. Li, H. P. Hratchian, A. F. Izmaylov, J. Bloino, G. Zheng, J. L. Sonnenberg, M. Hada, M. Ehara, K. Toyota, R. Fukuda, J. Hasegawa, M. Ishida, T. Nakajima, Y. Honda, O. Kitao, H. Nakai, T. Vreven, J. J. A. Montgomery, J. E. Peralta, F. Ogliaro, M. Bearpark, J. J. Heyd, E. Brothers, K. N. Kudin, V. N. K. Staroverov, R., J. Normand, K. Raghavachari, A. Rendell, J. C. Burant, S. S. Iyengar, J. Tomasi, M. Cossi, N. Rega, N. J. Millam, M. Klene, J. E. Knox, J. B. Cross, V. Bakken, C. Adamo, J. Jaramillo, R. Gomperts, R. E. Stratmann, O. Yazyev, A. J. Austin, R. Cammi, C. Pomelli, J. W. Ochterski, R. L. Martin, K. Morokuma, V. G. Zakrzewski, G. A. Voth, P. Salvador, J. J. Dannenberg, S. Dapprich, A. D. Daniels, Ö. Farkas, J. B. Foresman, J. V. Ortiz, J. Cioslowski, D. J. Fox Gaussian 09,Revision A.02; Gaussian, Inc., Wallingford CT: **2009**

[13] a) S. Grimme, *J. Comput. Chem.* **2006**, *27*, 1787-1799; b) A. D. Becke, *J. Chem. Phys.* **1997**, *107*, 8554-8560; c) Q. Wu, W. Yang, *J. Chem. Phys.* **2002**, *116*, 515-524; d) Y. Minenkov, Å. Singstad, G. Occhipinti, V. R. Jensen, *Dalton Trans.* **2012**, *41*, 5526-5541.

[14] W. R. Wadt, P. J. Hay, *J. Chem. Phys.* **1985**, *82*, 284-298.

[15] A. V. Marenich, C. J. Cramer, D. G. Truhlar, *J. Phys. Chem. B* **2009**, *113*, 6378-6396.

[16] P. J. Stephens, F. J. Devlin, C. F. Chabalowski, M. J. Frisch, *J. Phys. Chem.* **1994**, *98*, 11623-11627.

[17] a) L. Trefonas, W. N. Lipscomb, *J. Chem. Phys.* **1958**, *28*, 54-55; b) J. R. Durig, J. W. Thompson, J. D. Witt, J. D. Odom, *J. Chem. Phys.* **1973**, *58*, 5339-5343.

[18] a) J. N. Gayles, J. Self, *J. Chem. Phys.* **1964**, *40*, 3530-3539; b) A. Szabó, A. Kovács, G. Frenking, *Z. Anorg. Allg. Chem.* **2005**, *631*, 1803-1809.

[19] A. Finch, I. Hyams, D. Steele, *Spectrochim. Acta* **1965**, *21*, 1423-1431.

[20] R. J. Thompson, J. C. Davis, *Inorg. Chem.* **1965**, *4*, 1464-1467.

[21] H. Bonadeo, E. Silberman, *J. Mol. Spectrosc.* **1969**, *32*, 214-221.

[22] C. D. Good, D. M. Ritter, *J. Am. Chem. Soc.* **1962**, *84*, 1162-1166.

[23] D. E. Young, G. E. McAchran, S. G. Shore, *J. Am. Chem. Soc.* **1966**, *88*, 4390-4396.

[24] G. Sheldrick, *Acta Crystallogr. Sect. A: Found. Crystallogr.* **2015**, *71*, 3-8.

[25] G. Sheldrick, *Acta Crystallogr. Sect. A: Found. Crystallogr.* **2008**, *64*, 112-122.
